# Supplementary material for: Parkin drives pS65‐Ub turnover independently of canonical autophagy in Drosophila
Source: EMBO Rep. 2022 Oct 17;23(12):e53552. doi: 10.15252/embr.202153552 (PMC9724668; doi:10.15252/embr.202153552)
Supplement: Supplementary file 1 — Expanded View Figures PDF [file EMBR-23-e53552-s004.pdf]

Expanded View Figures

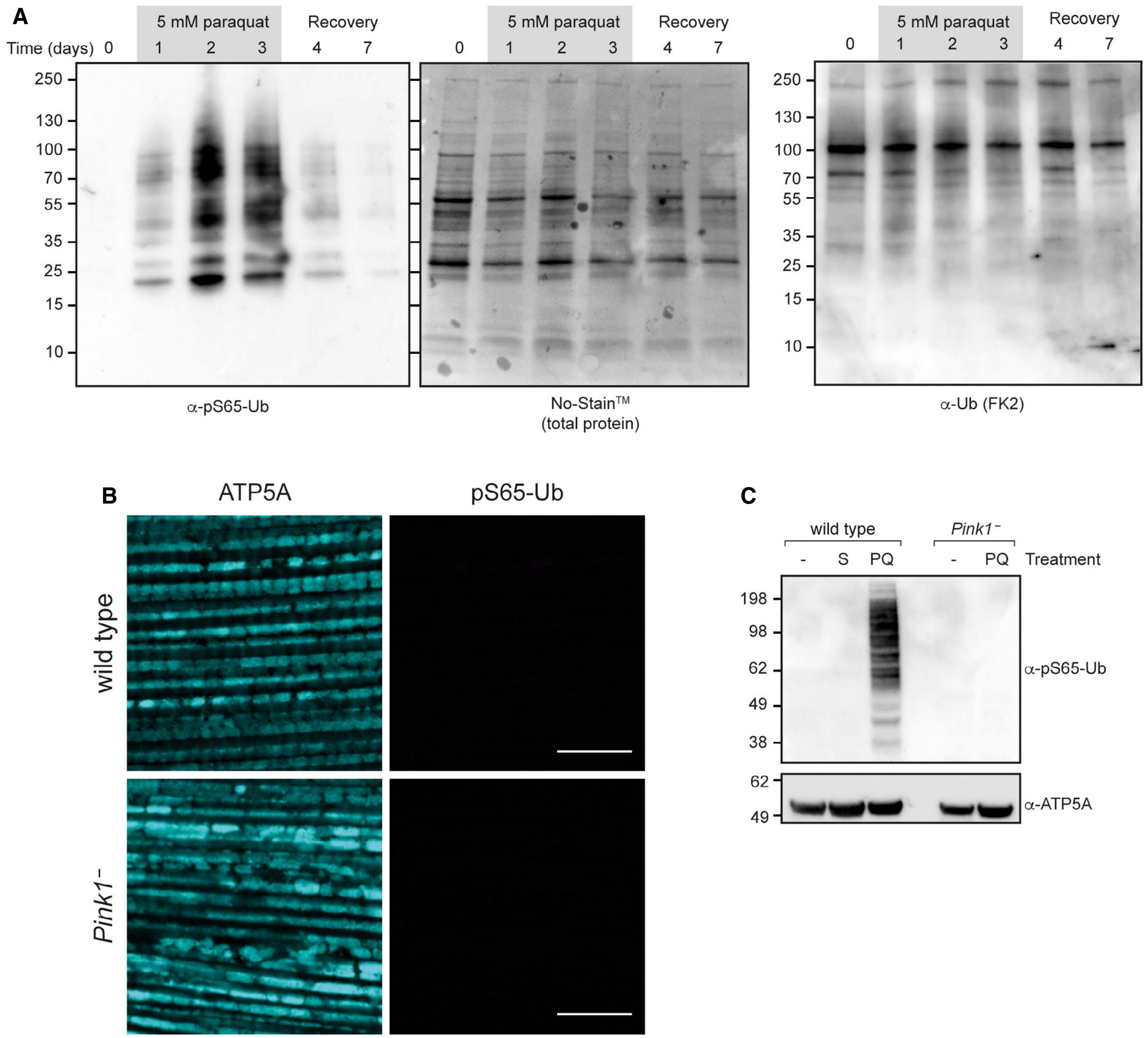

Figure EV1. Dynamics of pS65-Ub production.

A Total protein and immunoblots for the indicated antibodies of mitochondria-enriched fractions from wild-type flies treated for the indicated number of days with paraquat. Recovery, return to normal food.

B Immunostaining of flight muscles of *Pink1*⁻ and wild-type flies. Signal acquisition, brightness and contrast settings for pS65-Ub are identical to those presented in Fig 3D.

C pS65-Ub immunoblot of mitochondrial fractions from wild-type and *Pink1*⁻ flies following either no treatment (–) or after treatment for 3 days with sucrose (S) or paraquat (PQ).

© 2022 The Authors

Figure EV2. Loss of rdgC (PPEF2) does not affect pS65-Ub degradation *in vivo*.

Immunoblots for the indicated antibodies of whole-fly lysates treated with paraquat (PQ) followed by recovery on normal food. UT, untreated flies. *LacZ* RNAi serves as control for comparison with knockdown of *rdgC* (the fly homologue of PPEF2). Chart shows the mean +/− SEM of equivalent blots from three biological replicates.

 © 2022 The Authors

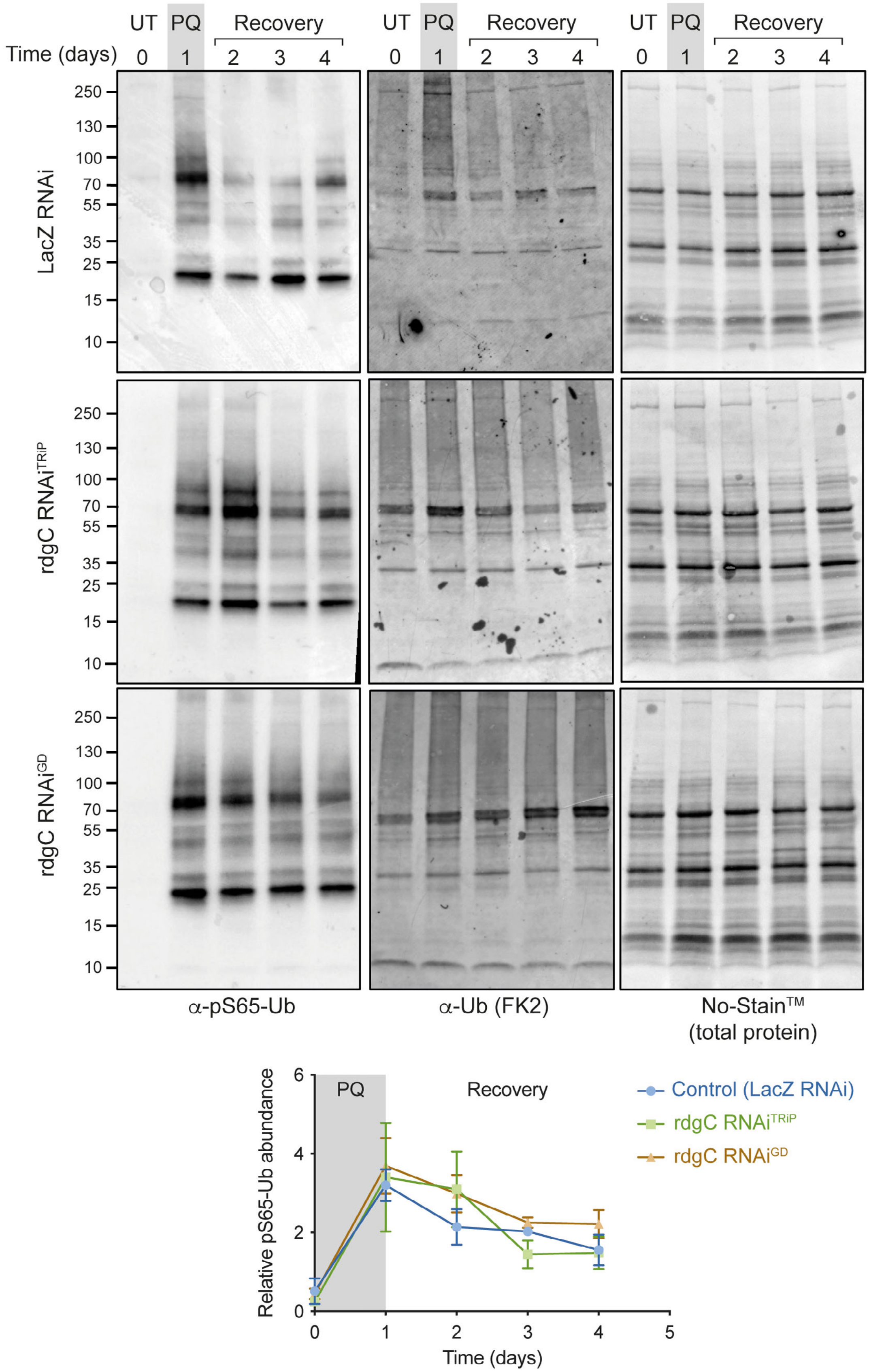
UT
PQ
Recovery
Time (days)
0 1 2 3 4
LacZ RNAi
rdgC RNAi^TRiP
rdgC RNAi^GD
250
130
100
70
55
35
25
15
10
α-pS65-Ub
α-Ub (FK2)
No-Stain™ (total protein)
Relative pS65-Ub abundance
Time (days)
Control (LacZ RNAi)
rdgC RNAi^TRiP
rdgC RNAi^GD

Figure EV2.

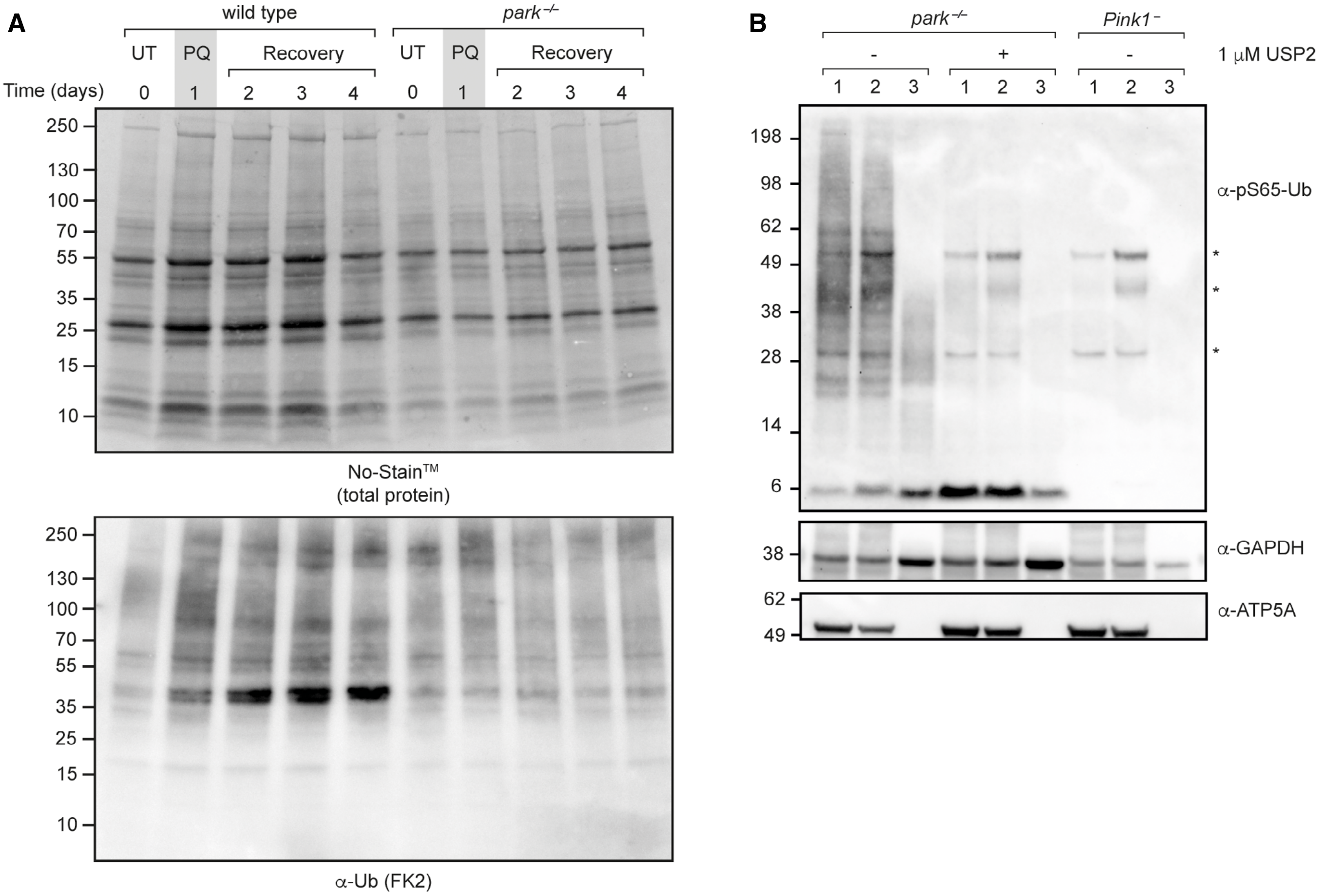

Figure EV3. pS65-Ub accumulates in *park*$^{-/-}$ flies.

A Immunoblots for the indicated antibodies as controls for samples analysed in Fig 2C.

B pS65-Ub immunoblot following subcellular fractionation and USP2 treatment as indicated. (1) 10,000 × *g* pellet; (2) 21,000 × *g* pellet; (3) 21,000 × *g* supernatant. Asterisk (*) denotes nonspecific bands.

 © 2022 The Authors

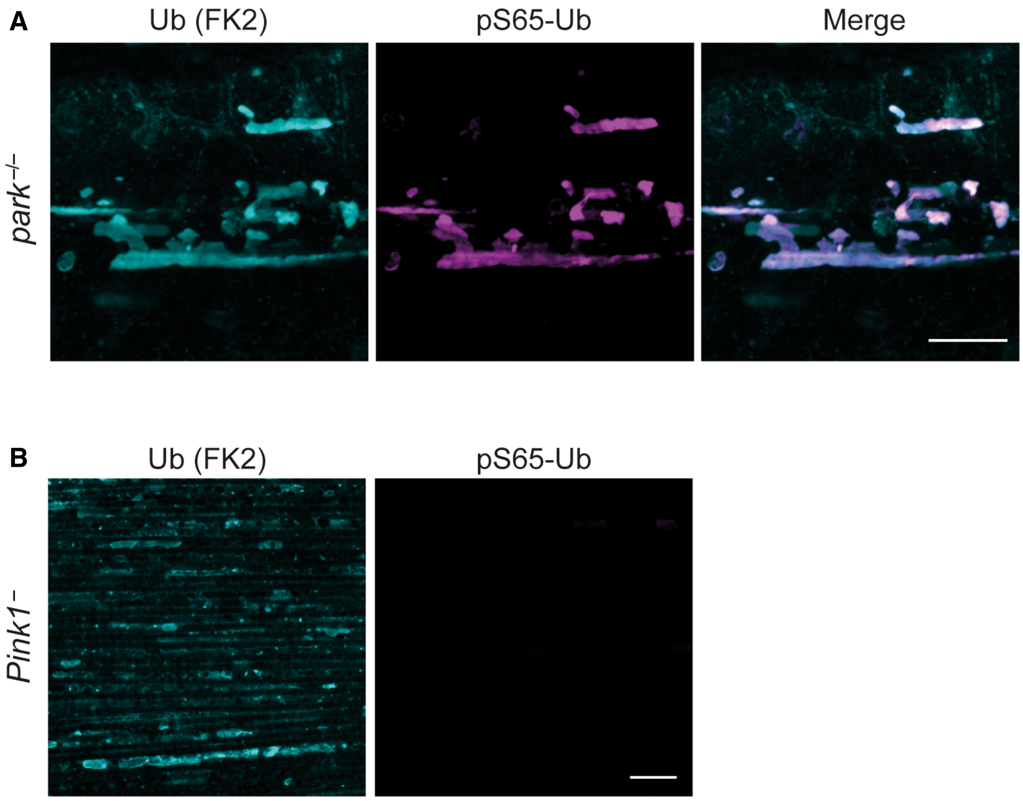

Figure EV4. Pink1-dependent pS65-Ub colocalises with Ub on *park*$^{-/-}$ mitochondria.

A, B Flight muscles from young, untreated (A) *park*$^{-/-}$ and (B) *Pink1*$^{-}$ flies immunostained for conjugated Ub (FK2) and pS65-Ub.

Data information: Scale bars = 10 μm.

Figure EV5. Dynamics of pS65-Ub production/turnover in autophagy mutants.

A pS65-Ub immunoblot of mitochondrial fractions from wild-type and *Atg5*$^{-}$ flies harvested at the indicated ages. Asterisk (*) denotes nonspecific band.
B pS65-Ub immunoblotting in whole-animal lysates from wandering L3 larvae of the indicated genotypes.
C pS65-Ub immunoblot of mitochondrial fractions from wild-type and *Atg5*$^{-}$ flies following a paraquat (PQ) pulse-chase assay. UT, untreated; Recovery, return to normal food.
D Quantification of pS65-Ub lane densitometry from n = 3 independent replicates of (B), expressed relative to the most intense band in each blot. Charts show mean ± SEM.

© 2022 The Authors

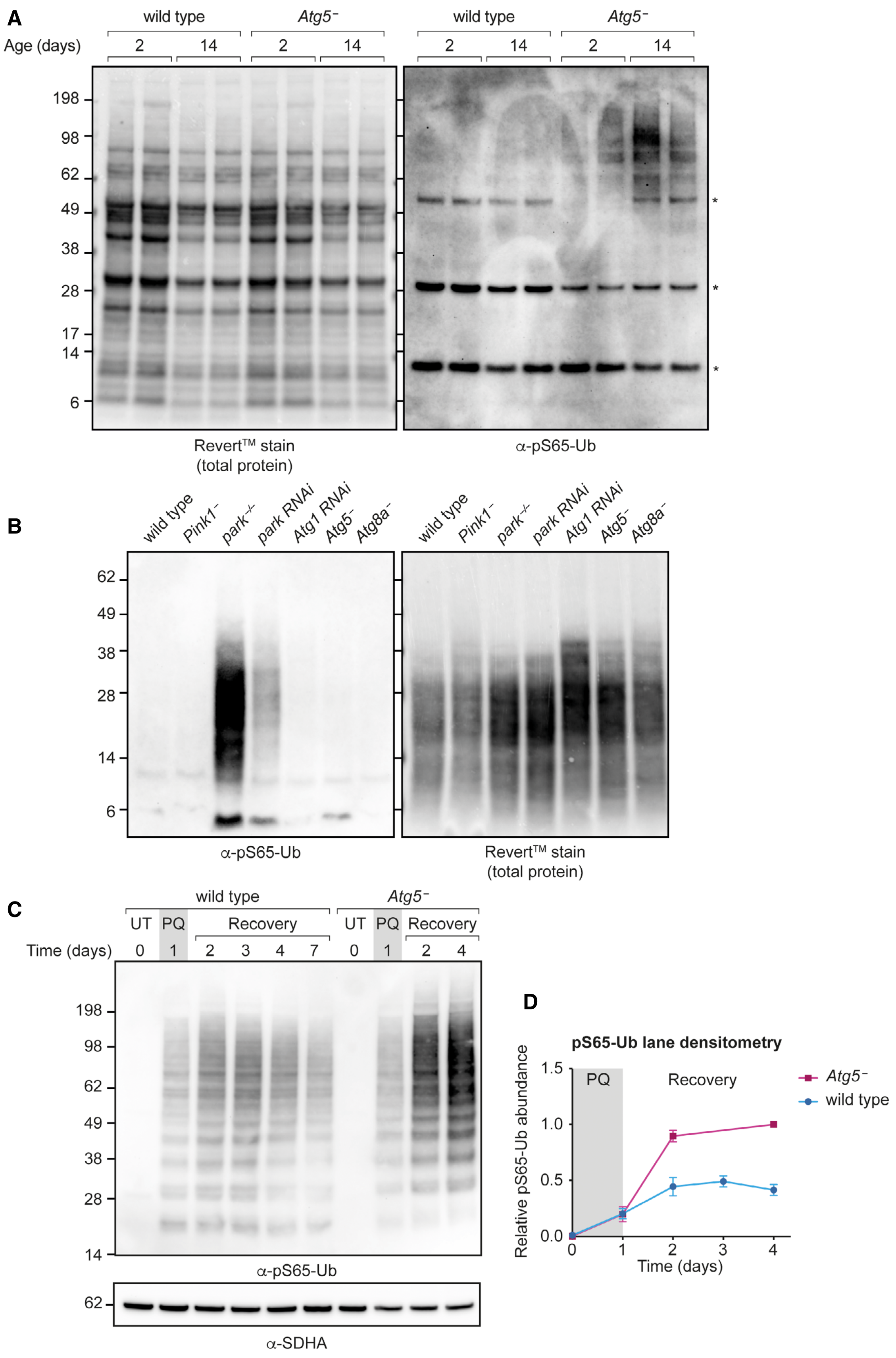

Figure EV5.

© 2022 The Authors

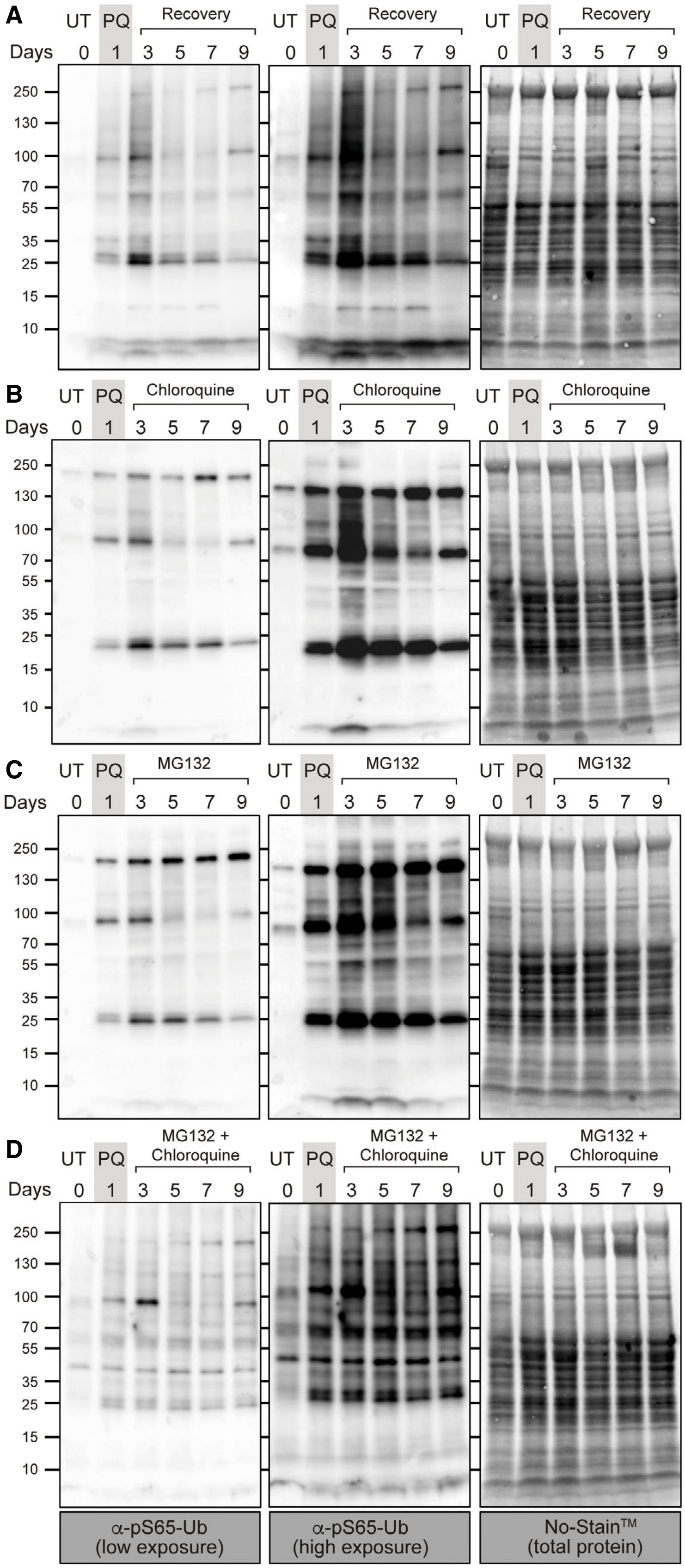

Figure EV6. Impact of lysosome and proteasome inhibitors on pS65-Ub degradation.

A–D pS65-Ub immunoblots of whole-fly lysates treated with paraquat (PQ) followed by recovery on filter papers with sucrose solution only (A) or dosed with (B) chloroquine (2 mM), (C) MG132 (50 μM) or both (D). Blots are representative of duplicate experiments.
