# Supplementary material for: Parkin drives pS65‐Ub turnover independently of canonical autophagy in Drosophila
Source: EMBO Rep. 2022 Oct 17;23(12):e53552. doi: 10.15252/embr.202153552 (PMC9724668; doi:10.15252/embr.202153552)
Supplement: Supplementary file 3 — PDF+ [file EMBR-23-e53552-s003.pdf]

Article

TRANSPARENT PROCESS | OPEN ACCESS

EMBO reports

Parkin drives pS65-Ub turnover independently of canonical autophagy in *Drosophila*

Joanne L Usher[1,2,†], Alvaro Sanchez-Martinez[1], Ana Terriente-Felix[1], Po-Lin Chen[3], Juliette J Lee[1], Chun-Hong Chen[3] & Alexander J Whitworth[1,*]

Abstract

Parkinson's disease-related proteins, PINK1 and Parkin, act in a common pathway to maintain mitochondrial quality control. While the PINK1-Parkin pathway can promote autophagic mitochondrial turnover (mitophagy) following mitochondrial toxification in cell culture, alternative quality control pathways are suggested. To analyse the mechanisms by which the PINK1–Parkin pathway operates *in vivo*, we developed methods to detect Ser65-phosphorylated ubiquitin (pS65-Ub) in *Drosophila*. Exposure to the oxidant paraquat led to robust, Pink1-dependent pS65-Ub production, while pS65-Ub accumulates in unstimulated *parkin*-null flies, consistent with blocked degradation. Additionally, we show that pS65-Ub specifically accumulates on disrupted mitochondria *in vivo*. Depletion of the core autophagy proteins Atg1, Atg5 and Atg8a did not cause pS65-Ub accumulation to the same extent as loss of parkin, and overexpression of parkin promoted turnover of both basal and paraquat-induced pS65-Ub in an *Atg5*-null background. Thus, we have established that pS65-Ub immunodetection can be used to analyse Pink1-Parkin function *in vivo* as an alternative to reporter constructs. Moreover, our findings suggest that the Pink1-Parkin pathway can promote mitochondrial turnover independently of canonical autophagy *in vivo*.

Keywords *in vivo*; mitochondria; mitophagy; Parkinson's disease; phospho-ubiquitin
Subject Categories Autophagy & Cell Death; Organelles; Post-translational Modifications & Proteolysis
DOI 10.15252/embr.202153552 | Received 30 June 2021 | Revised 5 September 2022 | Accepted 20 September 2022 | Published online 17 October 2022
EMBO Reports (2022) 23: e53552

Introduction

Parkinson's disease (PD) is the second most common neurodegenerative disease, with the global burden of disease having more than doubled between 1990 and 2016 (GBD, 2018). Autosomal recessive mutations in the genes encoding the mitochondria-targeted kinase PINK1 and the E3 ubiquitin (Ub) ligase Parkin are associated with parkinsonism (Kitada *et al*, 1998; Valente *et al*, 2004). Loss of either homologue in *Drosophila* (designated as Pink1 and parkin, respectively) results in strikingly similar phenotypes of severe mitochondrial dysfunction and degeneration of the indirect flight muscles, as well as the degeneration of a subset of dopaminergic neurons, thus mimicking a key hallmark of PD (Greene *et al*, 2003; Whitworth *et al*, 2005; Clark *et al*, 2006; Park *et al*, 2006). Genetic interaction studies subsequently placed *Pink1* and *parkin* in a common pathway, with *parkin* downstream of *Pink1* (Clark *et al*, 2006; Park *et al*, 2006).

PINK1 phosphorylates both Ub and Parkin, each at their respective Ser65 residues (Kondapalli *et al*, 2012; Shiba-Fukushima *et al*, 2012; Kane *et al*, 2014; Kazlauskaite *et al*, 2014; Koyano *et al*, 2014). PINK1 is partially imported into healthy, polarised mitochondria via its N-terminal mitochondrial targeting sequence where it is cleaved and subsequently degraded in the cytosol by the N-end rule pathway (Yamano & Youle, 2013). PINK1 is activated upon stalling on the outer mitochondrial membrane (OMM), where it phosphorylates Ub (pS65-Ub) that is conjugated at low abundance to OMM proteins (Okatsu *et al*, 2015a, 2015b). Parkin resides in the cytosol in an autoinhibited state and is recruited to mitochondria by binding pS65-Ub (Okatsu *et al*, 2015b). pS65-Ub binding partially displaces Parkin's Ubl domain, which allows it to be phosphorylated by PINK1 (Gladkova *et al*, 2018). This second phosphorylation event results in a dramatic domain rearrangement that relieves Parkin's autoinhibitory contacts and allows it to ubiquitinate proteins in close proximity (Gladkova *et al*, 2018; Sauvé *et al*, 2018). The Ub conjugated by Parkin provides the further substrate for phosphorylation by PINK1, which in turn promotes further Parkin recruitment, thus constituting a feed-forward mechanism of mitochondrial pS65-ubiquitination (Ordureau *et al*, 2014). Both the structure of active Parkin and cell-based studies suggest that Parkin has low substrate selectivity (Gladkova *et al*, 2018; Koyano *et al*, 2019), and it has been found to predominantly produce K6, K11, K48 and K63 chains *in vitro* (Ordureau *et al*, 2014).

Much of our understanding of the function of PINK1 and Parkin have been derived from utilising chemical depolarisation of mitochondria in cultured cells in conjunction with Parkin overexpression

1 MRC Mitochondrial Biology Unit, Cambridge, UK
2 PNAC Division, MRC Laboratory of Molecular Biology, Cambridge, UK
3 National Institute of Infectious Diseases and Vaccinology, National Health Research Institutes, Zhunan, Taiwan
*Corresponding author. Tel: +441223252910; E-mail: a.whitworth@mrc-mbu.cam.ac.uk
†Present address: MSD R&D Innovation Centre, London, UK

©2022 The Authors. Published under the terms of the CC BY 4.0 license.

(Narendra *et al*, 2008; Vives-Bauza *et al*, 2010). These experiments established a paradigm for studying the PINK1-Parkin pathway; upon depolarisation, PINK1- and Parkin-mediated ubiquitination of OMM proteins leads to the recruitment of the Ub-binding mitophagy receptors OPTN and NDP52 (Lazarou *et al*, 2015), which in turn promote autophagosome initiation (Boyle *et al*, 2019; Yamano *et al*, 2020), ultimately leading to degradation of the damaged mitochondria via the autophagy system. However, studies in animal models have provided mixed results as to the contribution of PINK1 and Parkin to mitophagy. While proteomic analysis of mitochondrial protein turnover in *Drosophila* supports a role for Pink1/parkin in promoting the degradation of mitochondrial membrane components (Vincow *et al*, 2013), imaging analysis of pH-sensitive fluorescent mitophagy reporters has yielded conflicting results (Cornelissen *et al*, 2018; Lee *et al*, 2018; McWilliams *et al*, 2018; Kim *et al*, 2019; Liu *et al*, 2021). It has also been shown in cell culture models that treatment with antimycin A or expression of an aggregation-prone matrix protein, ΔOTC, which does not abolish mitochondrial membrane potential, led to the production of mitochondria-derived vesicles (MDVs) in a PINK1- and Parkin-dependent manner (McLelland *et al*, 2014; Burman *et al*, 2017). Other studies have focussed on the role of PINK1 and Parkin in mitochondrial biogenesis, protein import and in the regulation of the fission and fusion machinery (Poole *et al*, 2008; Stevens *et al*, 2015; Jacoupy *et al*, 2019). However, many questions remain about the mechanisms of PINK1-Parkin-mediated mitochondrial quality control *in vivo*.

We sought to illuminate the physiological mechanisms of the PINK1-Parkin pathway by monitoring pS65-Ub levels as a direct measure of PINK1 activity. We developed complementary mass spectrometry, immunoblotting and immunostaining methods to detect pS65-Ub using *Drosophila* as a model system (hence, we adopt the FlyBase nomenclature "Pink1" and "parkin"). We confirm that pS65-Ub is produced by *Drosophila* Pink1 *in vivo* and can therefore be utilised to follow activation of the Pink1-parkin pathway and the downstream mechanisms of mitochondrial turnover. We identify exposure to the oxidant and parkinsonian toxin paraquat as a potent activator of Pink1 and establish this approach as a new paradigm to study the Pink1-parkin pathway *in vivo*.

Results

Development of methods to detect pS65-Ub *in vivo*

To understand the role of the Pink1-parkin pathway in maintaining mitochondrial quality control *in vivo*, we developed a mass spectrometry approach to detect low-abundance pS65-Ub. Adapting a recently described sample preparation pipeline (Swatek *et al*, 2019), we first determined the absolute abundance of total Ub and pS65-Ub in mitochondrial extracts from young (2–3 days) and aged (50–60 days) flies from a wild-type background (w^{1118}). In young flies, Ub was present on mitochondria, but pS65-Ub was not reliably detected with this method (Fig 1A, young). By contrast, aged flies displayed elevated total mitochondrial Ub, and pS65-Ub was robustly detected (Fig 1A, aged). To gain a clearer insight into the basal levels of pS65-Ub in young wild-type animals, we adjusted our protocol in order to optimise pS65-Ub detection (see Materials and Methods). Consequently, we were indeed able to detect pS65-Ub in mitochondrial fractions from young flies (Fig 1B), thus confirming that pS65-Ub is present in young flies, albeit in very low abundance.

We next sought to identify naturalistic stimuli of the Pink1-parkin pathway *in vivo*. We found that mitochondrial stress induced by an mtDNA mutator model (*daG4 > mito-APOBEC1*; Andreazza *et al*, 2019) was able to modestly induce pS65-Ub in young flies (Fig 1A, mutator). By contrast, 3-day exposure to paraquat, an oxidant that has been epidemiologically linked to PD (Tanner *et al*, 2011) and a potent inducer of reactive oxygen species (Filograna *et al*, 2016), led to a robust increase in both total and pS65-Ub in mitochondrial fractions in young flies (Fig 1A, paraquat). In paraquat-treated flies, pS65-Ub comprised approximately 6% of the total mitochondrial Ub, which is less than the 10–30% Ub phosphorylation that has been observed using similar methods in depolarised cells (Ordureau *et al*, 2014; Swatek *et al*, 2019).

As an orthogonal validation of our mass spectrometry results, we evaluated immunodetection methods using an antibody recently characterised to specifically detect pS65-Ub (Watzlawik *et al*, 2021). Immunoblotting confirmed the robust induction of pS65-Ub and total mitochondrial Ub upon paraquat treatment, while pS65-Ub was not detected in response to amino acid starvation from a sucrose-only diet (Fig 1C). The paraquat-induced pS65-Ub co-enriched with mitochondria in crude subcellular fractions (Fig 1D). Interestingly, pS65-Ub levels appeared to be similar between heads (with proboscises removed to eliminate muscle contamination) and bodies (thorax and abdomens) with a possible enrichment in heads (Fig 1E). We also determined that after removal of paraquat there was a progressive reduction in pS65-Ub levels, presumably due to mitochondrial turnover (Fig EV1A).

Flight muscles are a major tissue affected by the loss of *Pink1* and *parkin* and so are a key site of Pink1/parkin activity. Immunofluorescence microscopy of the flight muscles of untoxified, aged flies revealed low but consistent detection of mitochondria (ATP5A-positive) that were enveloped in pS65-Ub (Fig 1F), while pS65-Ub-positive structures were rarely observed in young flies (Fig EV1B). These results suggest that the Pink1-parkin pathway is basally active in *Drosophila*, but that pS65-Ub levels are kept very low in young animals, likely due to infrequent and transient induction and/or efficient turnover.

pS65-Ub production in response to paraquat requires Pink1 but not parkin

We next sought to confirm the requirements for Pink1 and parkin in the ubiquitination of mitochondria under basal and paraquat-induced conditions. To this end, we determined the abundance of total Ub and pS65-Ub in mitochondrial extracts from wild-type, $Pink1^{-}$ ($Pink1^{B9}$) and $park^{-/-}$ ($park^{25}$) mutant flies by mass spectrometry. Under basal conditions, loss of Pink1 resulted in elevated total Ub levels that did not further increase upon exposure to paraquat (Fig 2A). Importantly, pS65-Ub was not detectable above background in $Pink1^{-}$ flies even upon exposure to paraquat (Figs 2B and EV1C), confirming the conserved and essential role of Pink1 in the phosphorylation of Ub at S65 in *Drosophila*. $park^{-/-}$ flies displayed modestly elevated total mitochondrial Ub that did not significantly increase further in response to paraquat (Fig 2A). By contrast, the

©2022 The Authors

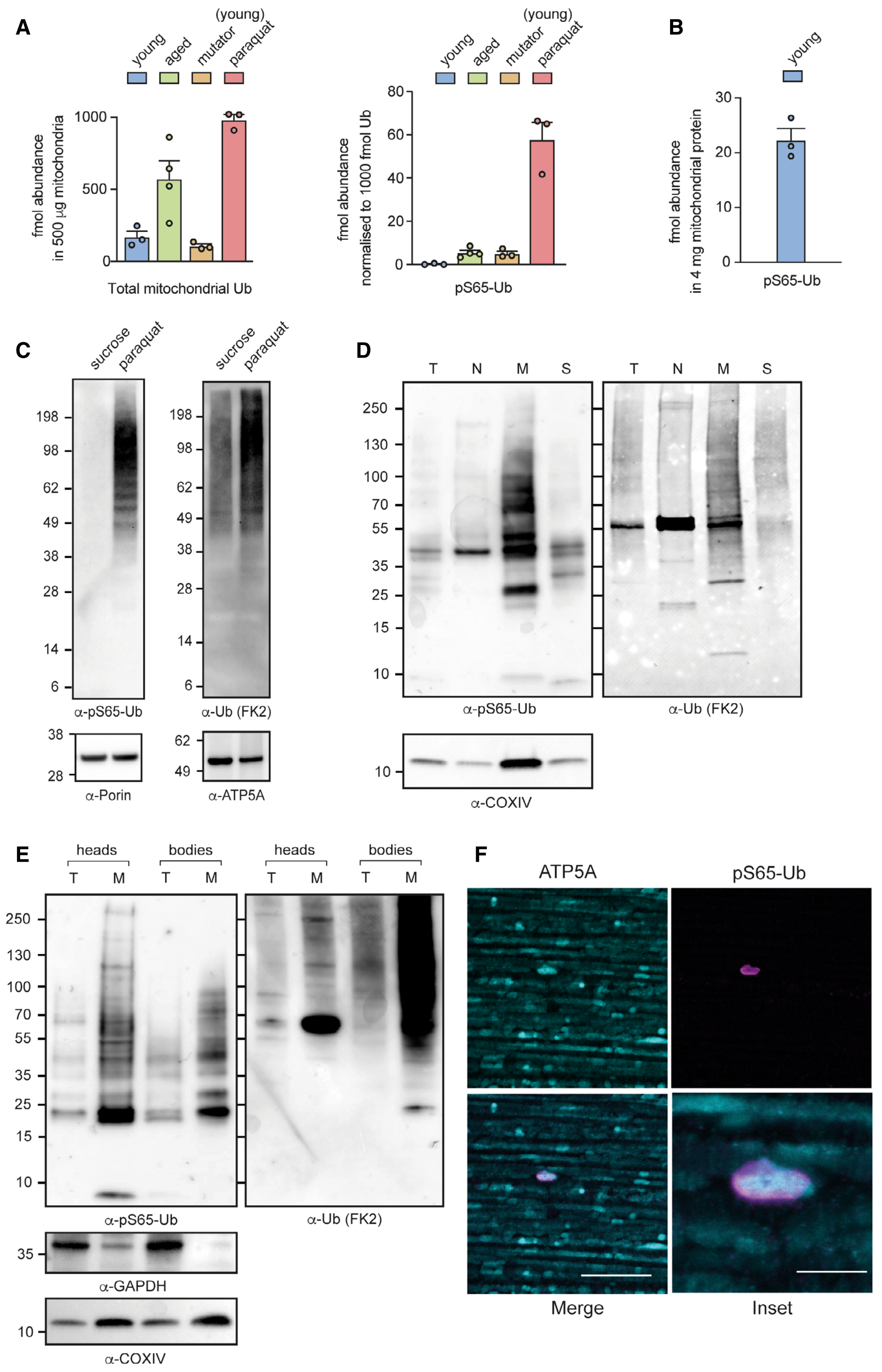
A
(young)
young
aged
mutator
paraquat
fmol abundance
in 500 μg mitochondria
1000
500
0
Total mitochondrial Ub
fmol abundance
normalised to 1000 fmol Ub
80
60
40
20
0
pS65-Ub
B
young
fmol abundance
in 4 mg mitochondrial protein
30
20
10
0
pS65-Ub
C
sucrose
paraquat
198
98
62
49
38
28
14
6
α-pS65-Ub
α-Ub (FK2)
α-Porin
α-ATP5A
D
T N M S
250
130
100
70
55
35
25
15
10
α-pS65-Ub
α-Ub (FK2)
α-COXIV
E
heads
bodies
T M
α-GAPDH
F
ATP5A
pS65-Ub
Merge
Inset

Figure 1.

Figure 1. Detection of pS65-Ub *in vivo*.

A Total Ub (left) and normalised pS65-Ub abundance (right) in 500 μg Ub-Clippase-treated, TUBE-enriched mitochondrial fractions from young (2–3 days) and aged (50–60 days) wild-type flies, an mtDNA mutator model (*daG4 > UAS-mito-APOBEC1*), and wild-type flies that had been exposed to paraquat (5 mM) for 3 days.

B Absolute abundance of pS65-Ub in 4 mg mitochondria from young (2–3 days) wild-type flies following sodium carbonate extraction, protease treatment and phospho-peptide enrichment.

C pS65-Ub (left) and total Ub (right) immunoblots of mitochondria-enriched fractions from wild-type flies treated for 3 days with either paraquat or vehicle (sucrose).

D Immunoblots for the indicated antibodies following subcellular fractionation of flies treated with paraquat for 3 days. T, total lysate; N, nuclear-enriched fraction; M, mitochondria-enriched fraction; S, postmitochondrial supernatant.

E Immunoblots for the indicated antibodies of (T) total lysates or (M) mitochondria-enriched fractions from fly heads (with proboscises removed) and bodies (thoraces and abdomens).

F Representative image of flight muscle from aged (50-day-old) wild-type animals immunostained with the indicated antibodies. Scale bars = 20 μm (inset, 5 μm).

Data information: Charts show mean ± SEM, n = 3–4 independent biological replicates as shown.

increase in pS65-Ub levels observed upon exposure to paraquat was largely unaffected by the loss of parkin (Fig 2B).

As an intermediate product in the Pink1-parkin pathway, pS65-Ub levels will be affected by the kinetics of both its production and downstream turnover, both of which could be impacted by the loss of parkin. We therefore devised a paraquat pulse-chase assay to probe the effect of loss of parkin on pS65-Ub dynamics. Flies were exposed to paraquat for 1 day (pulse) and then kept under normal conditions without paraquat (chase) before being analysed for pS65-Ub levels by immunoblotting. pS65-Ub accumulated upon paraquat exposure then declined over several days post-treatment, which we took to indicate turnover of the conjugated substrates (Fig 2C and D). Importantly, the loss of pS65-Ub was not due to the known pS65-Ub phosphatase PPEF2 (Wall *et al*, 2019), since the loss of the *Drosophila* homologue *rdgC* did not affect pS65-Ub degradation (Fig EV2). Another pS65-Ub phosphatase, PTEN-L (Wang *et al*, 2018), is not conserved in flies thus precluding its assessment.

In *park*$^{-/-}$ mitochondria, pS65-Ub levels were reduced at early time points compared with mitochondria from wild-type animals (Figs 2C and D, and EV3A), consistent with a diminished feed-forward cycle of Pink1-parkin-dependent pS65-Ub production in response to mitochondrial damage as previously described (Ordureau *et al*, 2014). By contrast, at later time points, pS65-Ub levels were elevated in *park*$^{-/-}$ mitochondria compared with wild-type flies, which likely reflects a defect in the turnover of damaged mitochondria (Figs 2C and D, and EV3A). It is therefore likely that parkin participates in the feed-forward cycle to promote further parkin recruitment to damaged mitochondria but is not strictly required for the production of pS65-Ub on mitochondria in response to paraquat.

To identify the turnover signal produced by parkin on damaged mitochondria, we next determined the pattern of paraquat-stimulated mitochondrial ubiquitination in the presence and absence of Pink1 or parkin. Analysing the four Ub chain types (linked at K6, K11, K48 and K63) that are most abundant on depolarised mitochondria and produced by Parkin *in vitro* (Ordureau *et al*, 2014), the relative proportions of all four chain types were unchanged in mitochondria from *Pink1*$^{-}$ and *park*$^{-/-}$ flies compared with wild-type animals in basal conditions (Fig 2E–H). In response to paraquat, only K6 chains increased in abundance on wild-type mitochondria, while K11 chains remained unchanged. Surprisingly, K48 and K63 chains decreased as a proportion of the total mitochondrial Ub, presumably due to a more substantial increase in monoubiquitination as previously described (Swatek *et al*, 2019). The paraquat-induced increase in K6 chains appeared to depend on Pink1 and parkin (Fig 2E). Our results are therefore consistent with other reports that the molecular function of parkin, rather than to amplify pS65-Ub, may be to produce either K6 chains or another Ub signal on the OMM following recruitment to damaged mitochondria by binding to pS65-Ub (Ordureau *et al*, 2015; Gersch *et al*, 2017).

pS65-Ub accumulates on disrupted mitochondria in *parkin* mutants

In our initial pipeline for the detection of pS65-Ub, we enriched mitochondria by relatively crude differential centrifugation. The pS65-Ub levels of untreated *park*$^{-/-}$ flies were not substantially elevated in these fractions, as determined by mass spectrometry (Fig 2B) and immunoblotting (Fig 2C, UT). However, Shiba-Fukushima *et al* (2017) had previously reported that pS65-Ub accumulates in *park*$^{-/-}$ flies by immunoblotting. When we analysed whole cell lysates, untreated *park*$^{-/-}$ animals displayed a striking abundance of pS65-Ub that was readily detectable by immunoblotting (Fig 3A), verifying the previous observation. We confirmed that this signal represented pS65-Ub as it was sensitive to treatment with the deubiquitinase USP2 (Fig EV3B). The effect was also observed upon ubiquitous knockdown of *parkin* (*daG4 > UAS-park RNAi*), confirming the specificity of the effect for loss of parkin (Fig 3B). The inducible RNAi line allowed us to assess the tissue distribution of the pS65-Ub in these flies using tissue-specific knockdowns. Interestingly, we found that the majority of the pS65-Ub originated from the muscle rather than neurons (Fig 3B). However, when we enriched for neural tissues by harvesting heads with proboscis muscle removed, some pS65-Ub was detectable in *park*$^{-/-}$ flies (Fig 3C). The higher relative abundance in muscle likely reflects that flight muscles have a very high mitochondrial content and are highly energetic, making them more prone to mitochondrial stress. This is consistent with a major requirement for Pink1-parkin in this tissue in flies and the occurrence of major phenotypes here (Greene *et al*, 2003; Clark *et al*, 2006; Park *et al*, 2006).

To better understand the subcellular localisation of pS65-Ub in *park*$^{-/-}$ flies, we first confirmed by immunoblot of biochemical fractions that pS65-Ub localises to cellular membranes as opposed to the cytosol (Fig EV1D). We then turned to an immunohistochemistry approach and saw numerous pS65-Ub-positive structures in the flight muscles of *park*$^{-/-}$ flies that were absent in wild-type and *Pink1*$^{-}$ flight muscles (Figs 3D and EV1B). These structures were

 ©2022 The Authors

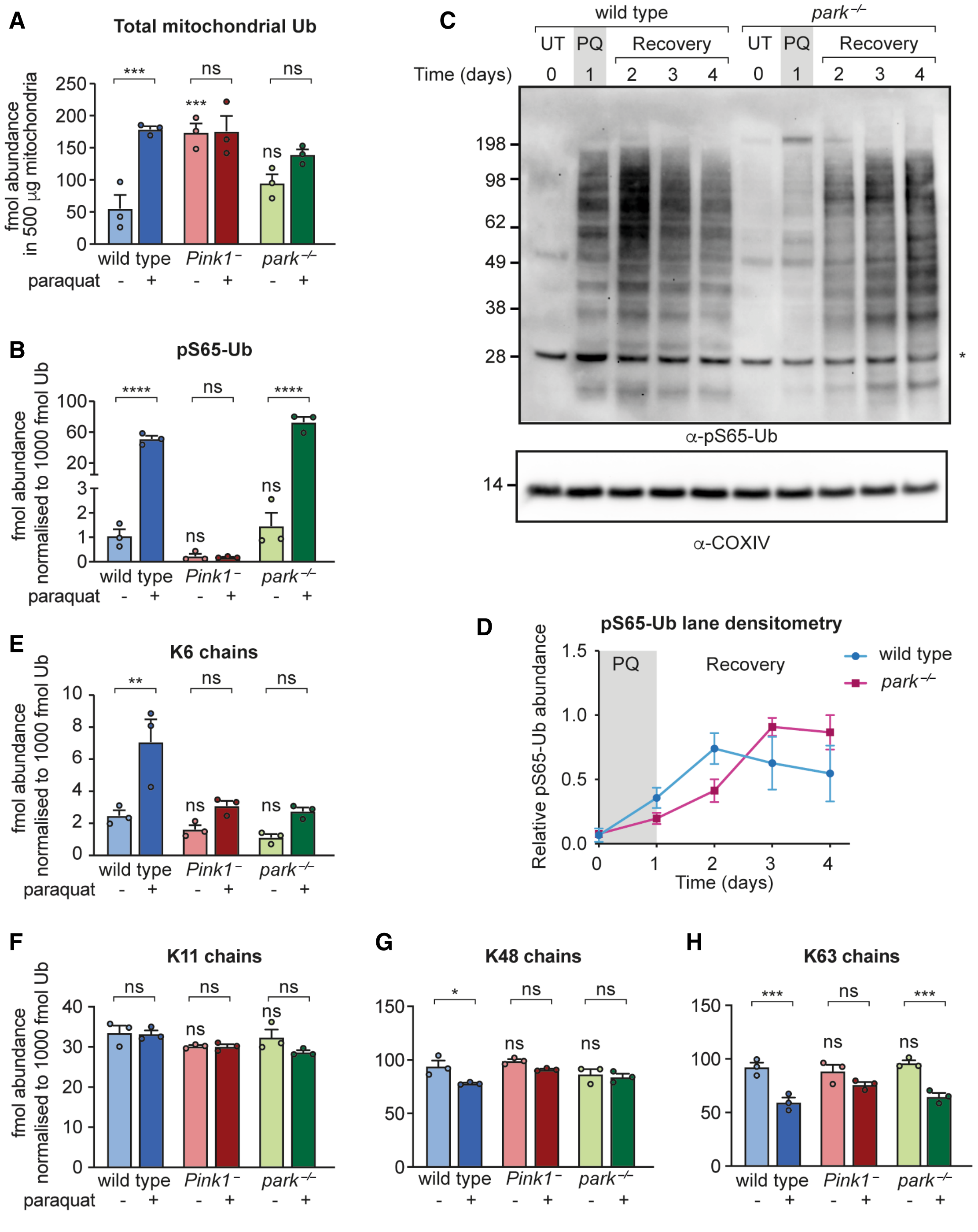

Figure 2. Analysis of the paraquat-induced mitochondrial ubiquitome from *Pink1*⁻ and *park*⁻/⁻ flies.

A, B (A) total Ub and (B) normalised pS65-Ub abundance in 500 μg TUBE-enriched, protease-treated mitochondrial fractions from wild-type, *Pink1*⁻ and *park*⁻/⁻ flies, either untreated young flies (2–3 days) or exposed to paraquat for 3 days.

C pS65-Ub immunoblot of mitochondria-enriched fractions following the paraquat pulse-chase assay in wild-type and *park*⁻/⁻ flies. UT, untreated; PQ, 1-day paraquat treatment; Recovery, flies removed from paraquat and returned to normal food. Asterisk (*) denotes nonspecific band. See Fig EV1C for related control blots.

D pS65-Ub lane densitometry analysis of $n = 3$ independent replicates from (C), expressed relative to the most intense lane signal in each blot. Charts show mean ± SEM.

E–H Relative abundance of (E) K6 chains, (F) K11 chains, (G) K48 chains and (H) K63 chains in mitochondrial fractions treated as in A, normalised to the total mitochondrial Ub in each sample. Charts show mean ± SEM from $n = 3$ independent biological replicates.

Data information: Statistical analysis used one-way ANOVA with the Šidák's correction for multiple comparisons. *$P < 0.05$; **$P < 0.01$; ***$P < 0.001$; ****$P < 0.0001$, ns = nonsignificant. The full list of multiplicity-adjusted P-values is presented in Dataset EV1.

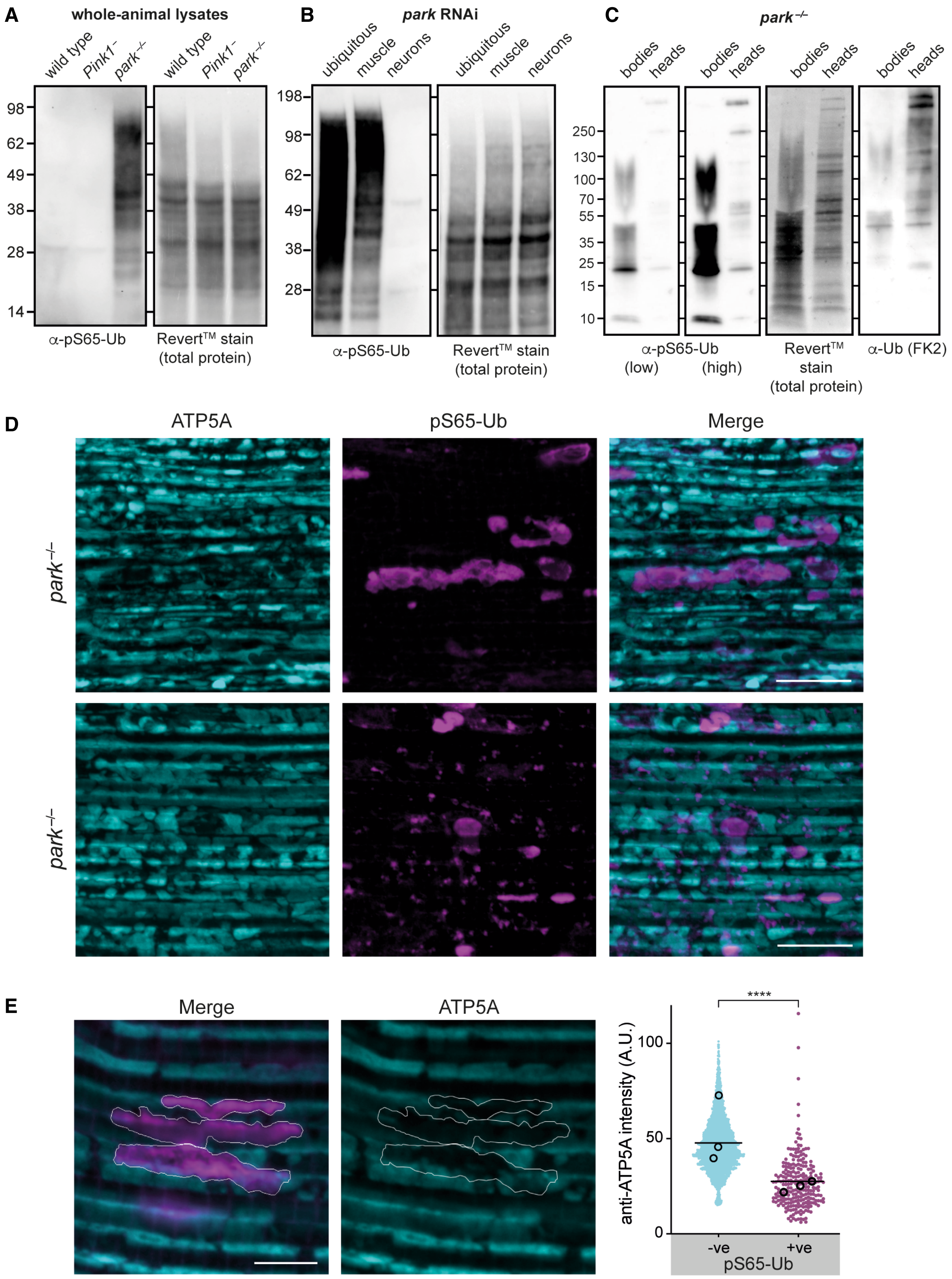

Figure 3.

 ©2022 The Authors

Figure 3. Tissue level analysis of pS65-Ub accumulation in *park*$^{-/-}$ flies.

A, B pS65-Ub immunoblot of whole-animal lysates from untreated flies of the indicated genotypes. *Park* RNAi was induced by *da-GAL4* (ubiquitous), *Mef2-GAL4* (muscle) or *nSyb-GAL4* (neurons).

C pS65-Ub immunoblot of lysates from bodies or heads (without proboscises), as indicated, from *park*$^{-/-}$ animals.

D Flight muscles from approximately 3-day-old untreated *park*$^{-/-}$ flies immunostained for ATP5A and pS65-Ub. Scale bars = 10 μm.

E Higher magnification image of pS65-Ub-positive structures in flight muscle counterstained for ATP5A. Scale bar = 5 μm. Chart shows quantification of ATP5A levels of pS65-Ub-positive (+ve) versus -negative (−ve) mitochondrial structures from three biological replicates (4–7 images per animal; average per animal shown in open circles). Line indicates mean. A.U., arbitrary units. ****$P < 0.0001$, Mann–Whitney test.

highly heterogeneous and ranged from small punctate structures (< 1 μm^3) to, more commonly, very large objects that resembled hyperfused or swollen mitochondria. Notably, mitochondria positive for pS65-Ub showed a consistently reduced immunostaining of ATP5A, an essential mitochondrial component (Fig 3E). Importantly, pS65-Ub structures clearly colocalised with a total Ub marker (Fig EV4A) and were absent in *Pink1*$^{-}$ flight muscles, despite the presence of similar structures that stained for total Ub (Fig EV4B), confirming them as *bone fide* pS65-Ub. Overall, these results are consistent with pS65-Ub accumulating on the OMM of dysfunctional mitochondria in the absence of parkin.

Since there is only very limited data on the ultrastructural distribution of pS65-Ub (Hou *et al*, 2018), and none to our knowledge *in vivo*, we further explored this by immuno-electron microscopy. As expected, wild-type and *Pink1*$^{-}$ flight muscle showed very little background pS65-Ub staining, distributed randomly with cytosol or myofibrils (Fig 4A and B; arrowheads). By contrast, *park*$^{-/-}$ flies showed abundant pS65-Ub immunostaining around mitochondrial cristae (Fig 4C). Interestingly, pS65-Ub was only observed on electrolucent mitochondria and was absent from more intact, electron-dense mitochondria (Fig 4Ci and ii). This neatly mirrors our observation of ATP5A depletion on pS65-Ub-positive structures by immunofluorescence. Together, these data indicate that pS65-Ub accumulates selectively on disrupted mitochondria *in vivo* in the absence of parkin.

Loss of core autophagy genes minimally affects pS65-Ub accumulation

The striking increase in pS65-Ub levels in *park*$^{-/-}$ flies indicated that the turnover of pS65-Ub was disrupted, which presents a paradigm to investigate the turnover mechanisms downstream of Pink1 and parkin as a more physiological alternative to genetic reporter constructs. Given the abundant evidence in cell culture models that PINK1-Parkin-mediated turnover occurs via the canonical autophagy machinery (Lazarou *et al*, 2007; Nguyen *et al*, 2016), we analysed pS65-Ub levels in mutants of core autophagy genes, *Atg1* (homologue of ULK1), *Atg5* and *Atg8a* (homologue of LC3/GABARAP). We saw a modest age-related increase in pS65-Ub levels in *Atg5*$^{-}$ (*Atg5*^{5cc5}) flies compared with wild-type animals (Fig EV5A), but surprisingly, this was very low compared with the increase in pS65-Ub levels observed in *park*$^{-/-}$ flies (Fig 5A). Consistent with this, neither loss of *Atg1* (*daG4 > Atg1 RNAi*) nor *Atg8a*$^{-}$ (*Atg8a*^{KG07569}) led to the same dramatic increase in pS65-Ub levels as loss of *park* (Figs 5B and EV5B).

Loss of parkin led to pS65-Ub production that was readily detectable by immunoblotting as early as the larval stage of development (Fig EV5B). To further probe whether the canonical autophagy machinery affects pS65-Ub production, we quantified the size and number of pS65-Ub-positive puncta in larval muscle. Wild-type and *Pink1*$^{-}$ larvae displayed no pS65-Ub puncta (Fig 6A, B and H), consistent with the absence of pS65-Ub observed by immunoblotting (Fig EV4B). By contrast, *park*$^{-/-}$ tissues displayed abundant pS65-Ub puncta (Fig 6C and H). *Atg5*$^{-}$ and *Atg8a*$^{-}$ larvae also displayed pS65-Ub puncta, although they were markedly fewer and generally smaller than those present in *park*$^{-/-}$ mutants (Fig 6D, E, H and I), while *Atg5*$^{-}$; *park*$^{-/-}$ and *Atg8a*$^{-}$; *park*$^{-/-}$ double mutants displayed puncta similar in number and size to *park*$^{-/-}$ alone (Fig 6F–I). These results suggest that canonical autophagy is not the major route for the turnover of pS65-Ub-positive structures under basal conditions. Notably, while *park*$^{-/-}$, *Atg5*$^{-}$ and *Atg8a*$^{-}$ animals are viable to the adult stage, *Atg5*$^{-}$; *park*$^{-/-}$ and *Atg8a*$^{-}$; *park*$^{-/-}$ double mutants are generally nonviable past the pupal stage, with only a few rare escapers, indicating synthetic lethality from the combined effect of independent pathways.

Parkin overexpression reduces pS65-Ub levels in the absence of Atg5

Although the loss of the core autophagy components Atg1, Atg5 and Atg8a did not result in the same extent of pS65-Ub accumulation as loss of parkin, loss of Atg5 or Atg8a did lead to modestly increased pS65-Ub levels compared with wild-type animals (Figs EV5A and 6H). In addition, in the paraquat pulse-chase assay *Atg5*$^{-}$ flies had elevated pS65-Ub at later time points relative to wild-type flies, suggestive of a block in turnover (Fig EV5C and D). These results are consistent with the autophagy machinery contributing at some level to basal turnover of damaged mitochondria and more substantially upon induction of mitochondrial damage using paraquat.

In order to further dissect whether the parkin-mediated pS65-Ub turnover is autophagy-dependent, we investigated the effect of parkin overexpression in an *Atg5*-null background (*Atg5*^{5cc5}; *daG4 > UAS-park*). We hypothesised that if the Pink1-parkin pathway proceeds primarily via autophagy, then parkin overexpression should either not affect or perhaps even further increase pS65-Ub levels in an *Atg5*$^{-}$ background. By contrast, if parkin drives autophagy-independent turnover, its overexpression should reduce pS65-Ub levels even in the absence of Atg5. We found in the paraquat pulse-chase assay that parkin overexpression substantially reduced pS65-Ub levels in an *Atg5*$^{-}$ background relative to an *Atg5*$^{-}$ mutant control (*Atg5*^{5cc5}; *daG4 > UAS-mito-HA-GFP*; Fig 7A and B). We further confirmed by mass spectrometry that while mitochondria from *Atg5*$^{-}$ flies displayed modestly elevated pS65-Ub levels, this could be reduced upon overexpression of parkin (Fig 7C). Taken together, these results indicate that parkin is able to drive pS65-Ub turnover independently of the canonical autophagy machinery in *Drosophila*.

©2022 The Authors

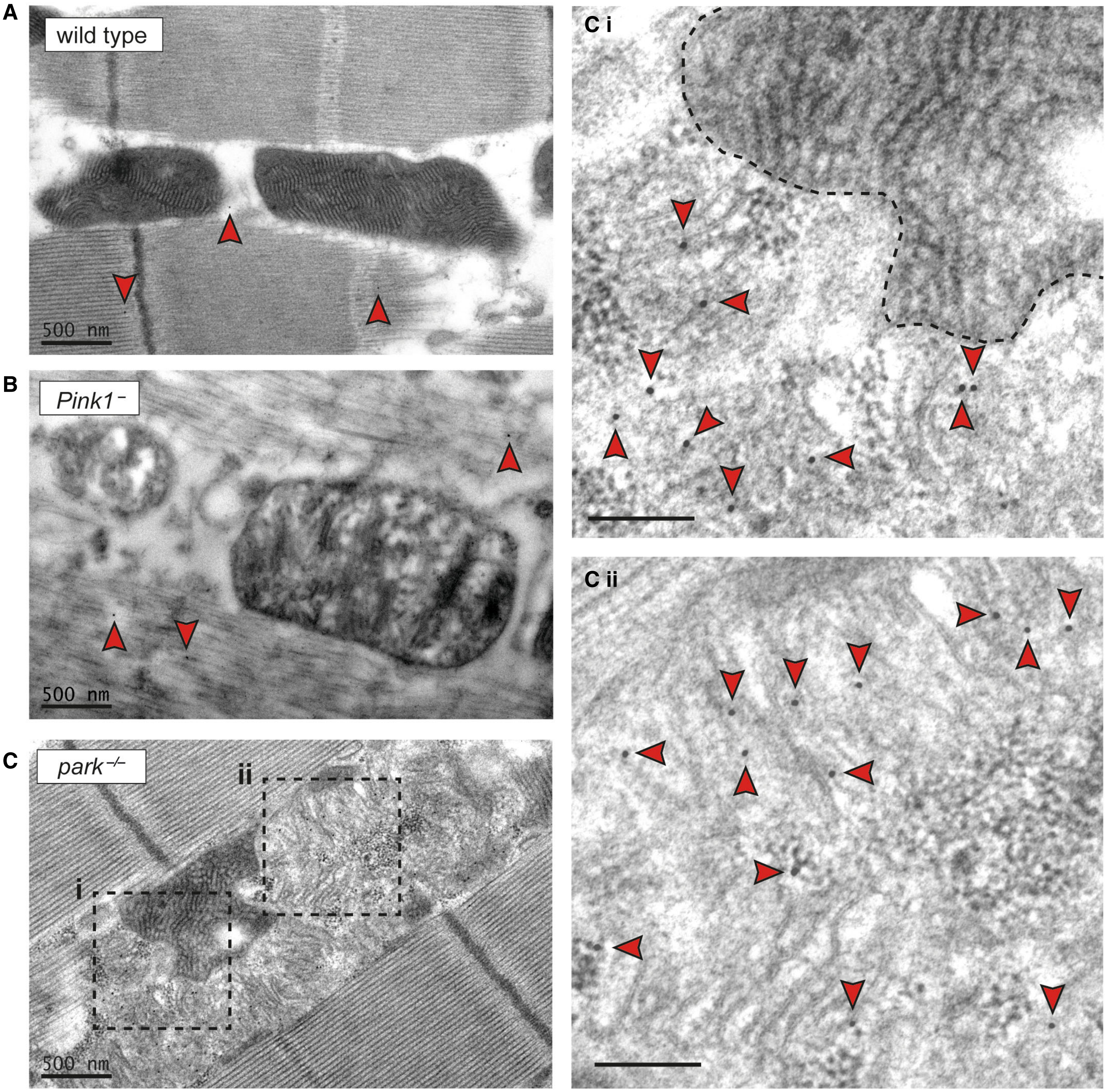

Figure 4. pS65-Ub accumulates on disrupted mitochondria *in vivo*.

A–C Immuno-electron microscopy of flight muscle from (A) wild-type, (B) *Pink1*$^{-}$ or (C) *park*$^{-/-}$ flies stained for pS65-Ub. Immuno-gold particles are highlighted (arrowheads). Scale bar = 500 nm. The numerous pS65-Ub immuno-gold particles are not labelled in (C), to avoid obscuring the image, but dashed boxes are magnified in the insets (Ci and Cii). Scale bar (insets) = 100 nm.

Finally, in an effort to determine the major degradation route of pS65-Ub, whether by lysosome or proteasome mechanisms, we treated wild-type flies with classic inhibitors of lysosome (chloroquine) or proteasome (MG132) during the recovery phase of the paraquat pulse-chase assay. Surprisingly, chemical inhibition of either lysosome or proteasome alone was not sufficient to substantially affect pS65-Ub degradation (Fig EV6A–C), while the combination of both inhibitors substantially blocked pS65-Ub degradation (Fig EV6D). These results suggest that both proteasome and lysosome mechanisms partially contribute to pS65-Ub turnover; however, the mechanism remains to be elucidated.

 ©2022 The Authors

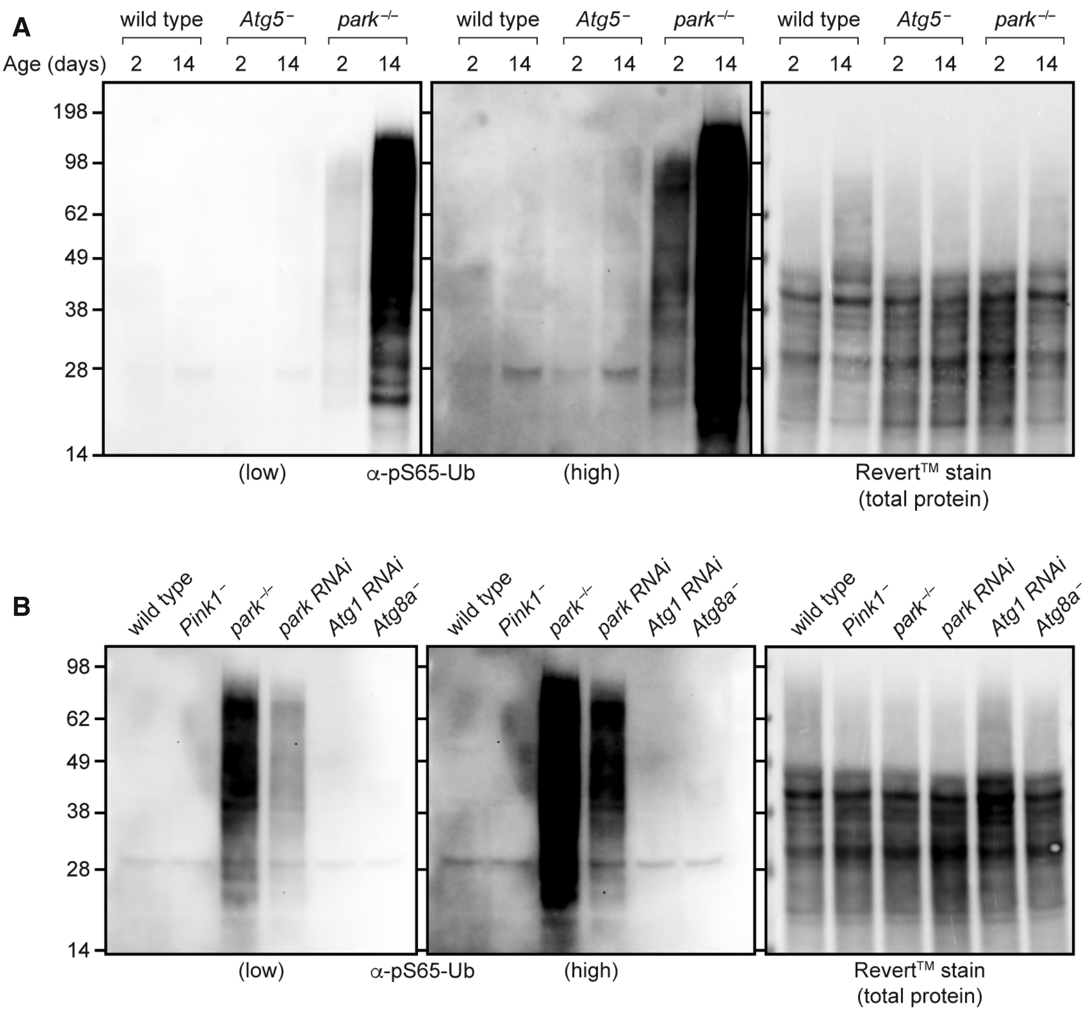

Figure 5. Loss of Atgs does not lead to the same degree of pS65-Ub accumulation as loss of parkin.

A pS65-Ub immunoblot of whole-animal lysates from wild-type, *Atg5*$^{-}$ and *park*$^{-/-}$ animals harvested at the indicated ages.

B pS65-Ub immunoblot of whole-animal lysates from young animals of the following genotypes: wild-type, *Pink1*$^{-}$, *park*$^{-/-}$, *park RNAi* (*daG4 > UAS-park RNAi*), *Atg1 RNAi* (*daG4 > UAS-Atg1 RNAi*) and *Atg8a*$^{-}$.

Discussion

We have developed mass spectrometry and immunodetection methods to monitor physiological levels of pS65-Ub as a direct and specific readout of Pink1 activity in *Drosophila*, a preeminent model for dissecting the conserved functions of Pink1 and parkin. We have found that pS65-Ub is produced by Pink1 under basal conditions, albeit at very low levels. Our methods revealed that ~0.5% of total Ub on mitochondria from aged flies is Ser65-phosphorylated, and we further observed individual mitochondria that were enveloped in pS65-Ub. Although we could not reliably detect pS65-Ub in young flies without additional enrichment, we surmise that it is likely less than 0.1% of mitochondrial Ub. This suggests that under normal healthy conditions, in the absence of exogenous or accumulated endogenous stresses, Pink1 activation is either an extremely infrequent event or the pS65-Ub is very quickly degraded.

Importantly, in an effort to understand the physiological stimulus of PINK1, we have found that pS65-Ub deposition is strongly induced by transient exposure to the oxidant and PD-linked toxin paraquat. Elevated oxidative stress is a long-standing culprit in the pathogenesis of PD and an intuitive trigger for mitochondrial quality control systems. Interestingly, recent work has elucidated a likely mechanism for PINK1 regulation by Cys oxidation (Gan *et al*, 2022; Rasool *et al*, 2022). While these *in vitro* and oxidant-induced observations are compelling, it will be interesting to determine the endogenous origin of the Cys oxidation, and particularly, how this signal is relayed from the matrix to PINK1 on the outer mitochondrial surface. Nevertheless, our data support that oxidative stress is a key trigger for Pink1/parkin activity *in vivo*.

We observed that pS65-Ub accumulates to high levels in *Drosophila* parkin mutants in the absence of exogenous stimulation of the pathway, confirming a previous observation (Shiba-Fukushima *et al*, 2017). Further, we have explored the spatiotemporal dynamics of pS65-Ub deposition, which occurs specifically on disrupted, electrolucent mitochondrial structures depleted for the OXPHOS subunit ATP5A. In response to paraquat, *park*$^{-/-}$ flies displayed

©2022 The Authors

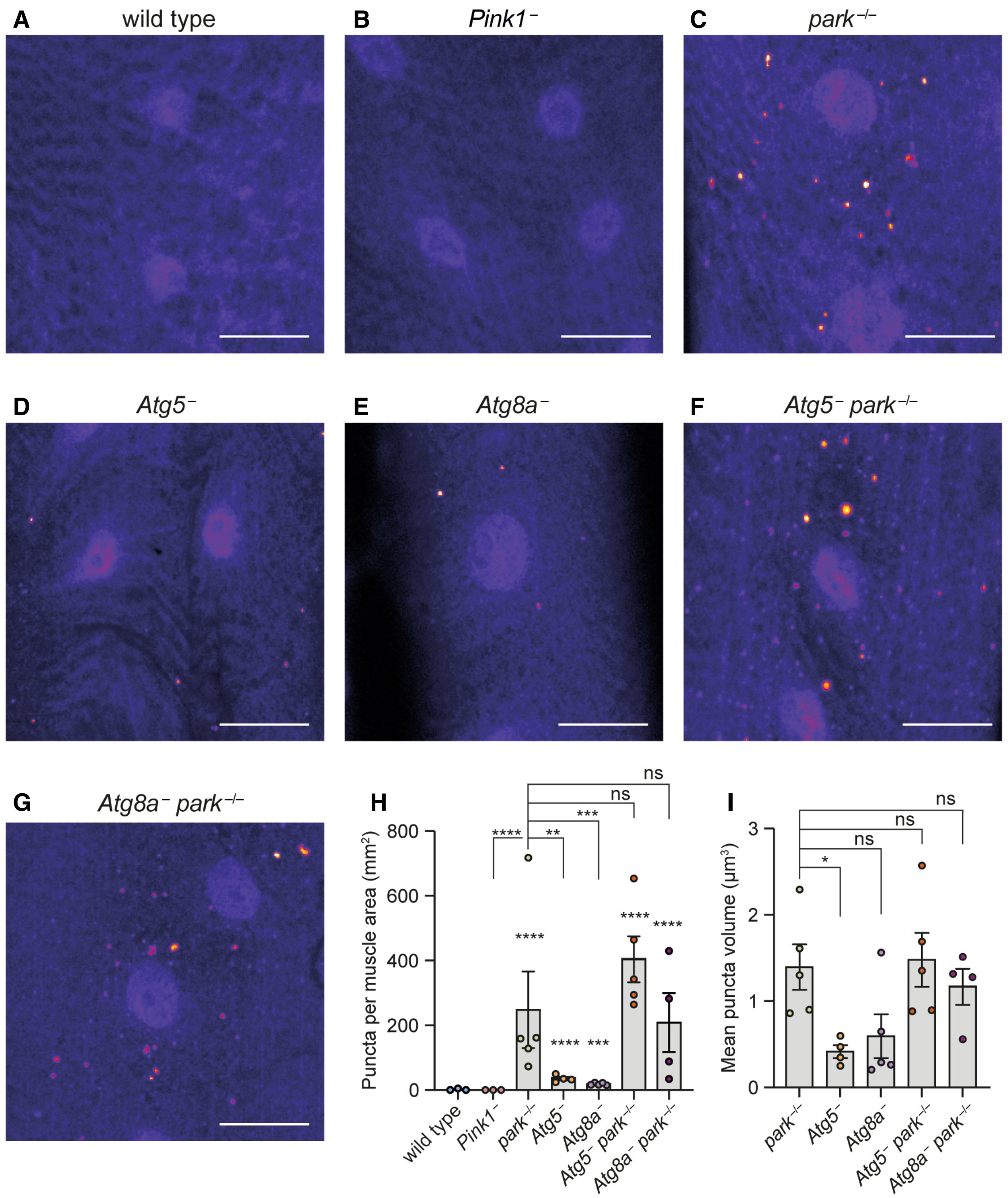

Figure 6. pS65-Ub immunostaining of larval muscle.

A–G Representative images of pS65-Ub immunostaining false-coloured for intensity (Fire LUT) in muscle segments 6/7 from wandering L3 larvae of the following genotypes: (A) wild-type, (B) *Pink1*$^{-}$, (C) *park*$^{-/-}$, (D) *Atg5*$^{-}$, (E) *Atg8a*$^{-}$, (F) *Atg5*$^{-}$; *park*$^{-/-}$ and (G) *Atg8a*$^{-}$; *park*$^{-/-}$. Scale bar = 20 μm.

H Quantification of the number of pS65-Ub puncta from (A–G) displaying mean ± SEM from the indicated number of animals (n = 3–5 as indicated).

I Puncta volume from (C–G) expressed as mean ± SEM (n = 4–5 animals).

Data information: Statistical analysis used one-way ANOVA with the Dunnett's correction for multiple comparisons. Comparisons are against wild-type unless indicated. *$P < 0.05$; **$P < 0.01$; ***$P < 0.001$; ****$P < 0.0001$, ns = nonsignificant.

low pS65-Ub levels relative to wild-type flies at early time points and elevated pS65-Ub at later time points, suggestive of a defect in mitochondrial turnover. These observations indicate that pS65-Ub alone is insufficient to elicit mitochondrial turnover and therefore that parkin's molecular function is not simply to amplify the pS65-Ub signal produced by Pink1. Several earlier studies also support this conclusion: increased stoichiometry of mitochondrial Ub phosphorylation was found to be inhibitory to mitophagy receptor

 ©2022 The Authors

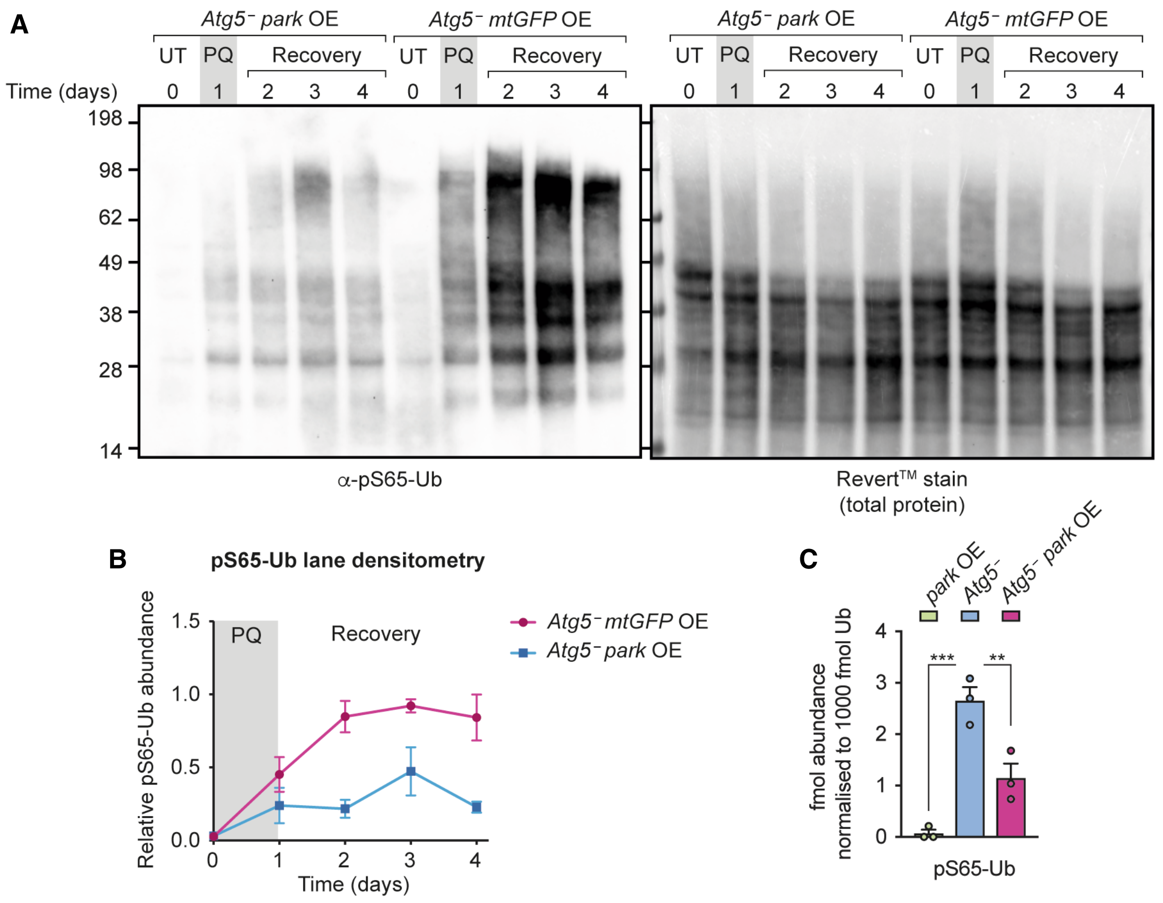

Figure 7. Parkin overexpression reduces pS65-Ub levels in an *Atg5⁻* background.

A pS65-Ub immunoblot of whole-animal lysates following paraquat pulse-chase assay of *Atg5⁻* flies with parkin overexpression (OE) (*Atg5[5cc5] daG4 > UAS-park*) compared with *Atg5⁻* flies overexpressing mitochondrially targeted GFP (*Atg5[5cc5] daG4 > UAS-mito-HA-GFP*). UT, untreated flies; PQ, 1-day paraquat treatment; Recovery, flies removed from paraquat and returned to normal food.

B pS65-Ub lane densitometry, normalised to total protein (Revert™ stain), of $n = 3$ independent replicates of (A), expressed as pS65-Ub intensity relative to the most intense lane in each blot. Charts show mean ± SEM.

C Mass spectrometry analysis of normalised pS65-Ub levels in Ub-Clippase-treated, TUBE-enriched mitochondrial fractions from flies overexpressing parkin (*daG4 > UAS-park*), *Atg5⁻* flies and *Atg5⁻* flies overexpressing parkin (*Atg5[5cc5] daG4 > UAS-park*). Charts show mean ± SEM ($n = 3$). Statistical analysis used one-way ANOVA with the Dunnett's correction for multiple comparisons. $^{**}P < 0.01$; $^{***}P < 0.001$.

recruitment in cell culture studies (Ordureau *et al*, 2018), and *in vitro* binding studies using Ser65-phosphorylated Ub chains have found that Ub phosphorylation does not promote autophagy receptor binding (Heo *et al*, 2015; Ordureau *et al*, 2015). Indeed, to our knowledge, Parkin itself is the only protein that has been shown to bind preferentially to pS65-Ub (Wauer *et al*, 2015). Our findings therefore suggest that the function of pS65-Ub is primarily to recruit Parkin to damaged mitochondria, rather than to promote downstream organelle turnover.

Our investigation of Pink1- and parkin-dependent Ub chain types showed that K6 chains (and not K11, K48, K63 or bulk ubiquitination) were upregulated in response to paraquat in wild-type but not Pink1- or parkin-deficient animals. These results corroborate previous *in vitro* data showing that K6 chains are produced on damaged mitochondria by these enzymes and suggest that this signal could be important for parkin-dependent turnover of damaged mitochondria *in vivo*. We did not investigate the specific substrates that are ubiquitinated by parkin, but previous studies have found a wealth of OMM substrates ubiquitinated *in vivo* and in cell culture studies in a Parkin-dependent manner, some of which could be important signals for mitochondrial turnover (Martinez *et al*, 2017; Ordureau *et al*, 2018, 2020). Regardless, the increase in K6 chains that we observed is particularly interesting given that the only mitochondria-resident deubiquitinase, USP30, preferentially hydrolyses K6 chains (Cunningham *et al*, 2015; Wauer *et al*, 2015; Gersch *et al*, 2017; Sato *et al*, 2017). The functions of K6 chains are not well understood, especially *in vivo* (Swatek & Komander, 2016), and further work is warranted to elucidate the precise contribution of this atypical Ub chain type to mitochondrial quality control.

The dramatic increase in pS65-Ub levels upon loss of parkin allowed us to assess the machinery responsible for the downstream turnover of pS65-ubiquitinated mitochondria. Analysing the autophagy machinery, we observed that upon loss of the core autophagy components Atg1, Atg5 or Atg8a, pS65-Ub levels were not affected

nearly to the extent observed in $park^{-/-}$ flies, which suggests that pS65-Ub is not primarily turned over via canonical autophagy under basal conditions. Moreover, parkin overexpression was able to reduce both basal and paraquat-induced pS65-Ub levels in an *Atg5*⁻ background. These results add to the growing evidence that the Pink1-parkin pathway may, under more physiological conditions, promote the turnover of damaged mitochondrial components in an autophagy-independent manner. For instance, Vincow *et al* (2013) analysed turnover rates of mitochondrial proteins in *Drosophila* and found that Pink1 and parkin were required for the turnover of a subset of IMM proteins that were distinct from those turned over by the core autophagy protein Atg7. Vincow *et al* also found some overlap between the proteins turned over by parkin and Atg7; similarly, we observed a slight accumulation of pS65-Ub in autophagy-deficient flies, consistent with some mitochondrial degradation occurring via a classic nonselective autophagy route. However, the Pink1-parkin pathway clearly has roles that are divergent from canonical autophagy, as $Atg5^{-}$; $park^{-/-}$ and $Atg8a^{-}$; $park^{-/-}$ double mutants showed synthetic lethality, and parkin overexpression was able to reduce both basal and paraquat-induced pS65-Ub levels in an $Atg5^{-}$ background. Consistent with this, the ability of parkin overexpression to rescue *Pink1* phenotypes has been shown to be unaffected by the loss of Atg1 or Atg18 (Liu & Lu, 2010).

Alternative mechanisms of parkin-mediated pS65-Ub turnover could be via direct proteasomal degradation of pS65-ubiquitinated OMM proteins (Tanaka *et al*, 2010; McLelland *et al*, 2018), or via MDV delivery directly to the lysosome (McLelland *et al*, 2014; Sugiura *et al*, 2014; Ryan *et al*, 2020). However, *in vivo* evidence for the existence of MDVs is limited, due in part to technical constraints in observing such small structures in complex tissues. Our preliminary data using chemical inhibitors of proteasome and lysosome suggest some contribution from both of these mechanisms, suggesting that there is plasticity and redundancy in the mechanisms of mitochondrial turnover *in vivo* as has been previously discussed in other contexts (Kocaturk & Gozuacik, 2018). Although the mechanistic details remain to be elucidated, we anticipate that the methods described herein could aid in analysing the presence of MDVs and their dependence on Pink1 and parkin *in vivo*, and to dissect the relative contribution of proteasome- and lysosome-dependent mechanisms of parkin-dependent mitochondrial turnover.

pS65-Ub as a biomarker for neurodegeneration

pS65-Ub has been proposed as a potential biomarker for neurodegenerative disease, with a recent study finding elevated pS65-Ub levels in blood samples from a cohort of Alzheimer's Disease patients compared with age-matched controls (Watzlawik *et al*, 2021). The cellular pathology underlying PD precedes classical symptom onset and therefore clinical diagnosis by many years, which is likely to hamper the success of clinical trials of potentially disease-modifying drugs as they may be given too late to halt disease progression (Stern *et al*, 2012). We found that pS65-Ub was readily detectable in $park^{-/-}$ animals in developmental stages prior to overt neurodegeneration, thereby suggesting that pS65-Ub accumulation is an early event that, if replicated in mammals, could have potential as an early-stage diagnostic biomarker. We also note the 100% sequence identity of Ub and conservation of the S65 phosphorylation site in *Drosophila* compared with mammals, meaning that the tools described herein can be applied to various species. However, pS65-Ub levels increased with healthy ageing in flies and were absent upon loss of Pink1, and we show a complex time-dependent effect on pS65-Ub levels in paraquat-treated $park^{-/-}$ flies. We therefore posit that a meaningful healthy range of pS65-Ub abundance would need to be established in order to use pS65-Ub levels as a clinical or preclinical biomarker.

In conclusion, we have developed methods to detect pS65-Ub at physiological levels in *Drosophila* and delivered the unanticipated finding that loss of parkin results in a striking elevation in pS65-Ub levels that is not recapitulated upon loss of the canonical autophagy genes *Atg5*, *Atg8a* or *Atg1*. We expect that the tools described herein will greatly aid in future studies dissecting the downstream mechanisms of mitochondrial turnover that are promoted by Pink1 and parkin *in vivo*.

Materials and Methods

Drosophila stocks and husbandry

Flies were raised under standard conditions in a temperature-controlled incubator with a 12 h:12 h light:dark cycle at 25°C and 65% relative humidity, on food consisting of agar, cornmeal, molasses, propionic acid and yeast. The following strains were obtained from the Bloomington *Drosophila* Stock Centre (RRID: SCR_006457): w^{1118} (RRID: BDSC_6326), *da-GAL4* (RRID: BDSC_55850), *Mef2-GAL4* (RRID: BDSC_27390), *nSyb-GAL4* (RRID: BDSC_51941), $Atg8a^{KG07569}$ (RRID: BDSC_14639), *UAS-mito-HA-GFP* (RRID: BDSC_8443), $UAS\text{-}rdgC^{HMC05070}$ *RNAi* (RRID: BDSC_60076) and $UAS\text{-}park^{HMS01800}$ *RNAi* (RRID: BDSC_38333). The $UAS\text{-}Atg1^{GD7149}$ *RNAi* (VDRC_16133) and $UAS\text{-}rdgC^{GD11966}$ RNAi (VDRC_35105) lines were obtained from the Vienna *Drosophila* Resource Center. $Pink1^{B9}$ flies were a kind gift from J. Chung (Park *et al*, 2006), and the $Atg5^{5cc5}$ stock was a kind gift from G. Juhasz (Kim *et al*, 2016). $UAS\text{-}park_{C2}$, $park^{25}$ and *UAS-mito-APOBEC1* lines have been described previously (Greene *et al*, 2003; Lee *et al*, 2018; Andreazza *et al*, 2019). Male flies only were used for experiments with adults, while experiments with larvae used both males and females except for animals with X chromosome balancers ($Pink1^{B9}$, $Atg5^{5cc5}$), for which only male animals were used. For ageing experiments, flies were maintained in bottles (MS experiments) or tubes (immunostaining experiments), transferred to fresh food 2–3 times per week and harvested after 50–60 days. Unless otherwise specified, young flies (2–3 days old) were used for all experiments.

Paraquat exposure assays

For MS experiments, flies were maintained in bottles (100–200 flies per replicate) containing 9 semi-circular pieces of filter paper (90 mm diameter, Cat#1001-090) saturated with 5% (w/v) sucrose solution containing 5 mM paraquat. Sucrose-only starvation experiments were performed as above, with the omission of paraquat. After 3 days, the flies were anaesthetised with mild CO_2 and live flies only were harvested. For pulse-chase experiments, 5–25 flies were harvested per replicate. The day 0 control was taken prior to paraquat treatment, and the remaining flies were incubated

©2022 The Authors

overnight in bottles containing paraquat as above. The next day, flies were anaesthetised with CO_2, dead flies were removed, a day 1 timepoint was taken and the remaining flies were divided among tubes of food with no more than 20 flies per tube. On day 2, all flies were flipped onto fresh food and then flipped every 2–3 days thereafter until harvest. For the lysosome and proteasome inhibitor pulse-chase experiments, w^{1118} flies were collected and treated with 10 mM paraquat as described above. After 1 day, flies were placed in tubes containing filter papers saturated with 5% (w/v) sucrose solution dosed with chloroquine (2 mM, Merck Life Science Limited, Cat#C6628) and/or MG132 (50 μM, Sigma Aldrich, Cat#C2211). Fresh solutions were applied every 2–3 days thereafter until harvest.

Mitochondrial enrichment by differential centrifugation

All steps were performed on ice or at 4°C. For mass spectrometry analysis, mitochondria were harvested from fresh (i.e. not frozen) flies according to (Lazarou *et al*, 2007), with modifications. Whole flies (60–200 per replicate) were placed in a Dounce homogeniser. Solution A (70 mM sucrose, 20 mM HEPES pH 7.6, 220 mM mannitol, 1 mM EDTA) containing cOmplete protease inhibitors (Roche) and PhosSTOP phosphatase inhibitors (Roche) was added (approximately 10 μl per fly), and the flies were homogenised with 35 strokes of a drill-fitted pestle. The homogenate was transferred to a 50 ml tube and incubated for 30 min, then centrifuged for 5 min at 1,000 × *g*. The supernatant was transferred to microcentrifuge tubes and centrifuged for 15 min at 10,000 × *g* to pellet mitochondria. The postmitochondrial supernatant was removed and the pellet was resuspended in Solution A. The homogenate was then clarified by centrifugation for 5 min at 800 × *g*, and the supernatant was transferred to a fresh tube. This clarification step was repeated once more to ensure all cuticle was removed from the sample. The supernatant was then centrifuged 10 min at 10,000 × *g*, and the postmitochondrial supernatant was discarded. The pellet was resuspended in Solution A and centrifuged for 10 min at 10,000 × *g*, and this wash step was repeated for a total of three times. The washed pellet was resuspended in Sucrose Storage Buffer (500 mM sucrose, 10 mM HEPES pH 7.6) and stored at −80°C until needed.

For immunoblotting analysis and biochemical fractionation from small numbers of flies (10–30), a modified mitochondrial enrichment procedure was performed. Flies were prepared either fresh or after flash-freezing in liquid nitrogen, with all direct comparisons performed with flies that were prepared in the same manner. Flies were transferred into a Dounce homogeniser containing 700 μl Solution A containing protease and phosphatase inhibitors as above and manually homogenised with 50 strokes of a pestle. The homogenate was transferred to an Eppendorf tube, a further 500 μl of Solution A was added to the homogeniser and the sample was homogenised with a further 10 strokes. The homogenates were pooled and incubated for 30 min, and then centrifuged for 5 min at 800 × *g*. The supernatant (containing mitochondria) was transferred to a new tube and clarified twice by centrifugation for 5 min at 1,000 × *g*. The clarified supernatant was then centrifuged for 10 min at 10,000 × *g* and the postmitochondrial supernatant was discarded or, in the case of biochemical fractionation experiments, further centrifuged for 30 min at 21,000 × *g*, and the pellet and supernatant retained for analysis. The mitochondrial pellet was washed once in Solution A containing only protease inhibitors, and then once in Solution A without inhibitors. The washed mitochondrial pellet was resuspended in 50–200 μl sucrose storage buffer, the protein content determined by BCA assay (Thermo Pierce) and stored at −80°C until needed.

USP2 treatment

For the validation of the pS65-Ub signal in *park*$^{-/-}$ samples, 30 μg protein per subcellular fraction was treated with the pan-specific deubiquitinase USP2 (BostonBiochem, E-506). The USP2 enzyme was diluted in buffer (50 mM Tris–pH 7.5, 50 mM NaCl, 10 mM DTT; Hospenthal *et al*, 2015) and then added to the subcellular fractions to a final USP2 concentration of 1 μM. The mixture was incubated for 45 min at 37°C prior to analysis by immunoblotting.

Mass spectrometry sample preparation and analysis

Absolute quantification (AQUA) analysis of Ub modifications was performed as previously described (Swatek *et al*, 2019), with modifications. Five hundred microgram mitochondria (from approximately 100 flies) prepared as above were resuspended in 250 μl TUBE lysis buffer (PBS containing 1% (v/v) NP-40, 2 mM EDTA, 10 mM chloroacetamide, cOmplete EDTA-free protease inhibitor cocktail (Roche)) supplemented with 8 μg/ml GST-Ubiquilin-UBA (Hjerpe *et al*, 2009; Fiil *et al*, 2013; Hrdinka *et al*, 2016). The lysate was incubated on ice for 20 min and then centrifuged for 15 min at 21,000 × *g*, 4°C. The clarified lysate was added to 20 μl Glutathione Sepharose 4B resin (GE Healthcare) that had been washed three times in TUBE lysis buffer and was incubated for 2 h at 4°C with gentle rotation. The lysate was removed and the beads were washed twice with PBS containing 0.1% (v/v) Tween 20, then twice with PBS. Eighty microliter Lb^{pro} reaction buffer (50 mM NaCl, 50 mM Tris–pH 7.4, 10 mM DTT) containing 20 μM Ub-clippase Lb^{pro} construct containing residues 29–195 with L102W mutation, purified as described previously (Guarne *et al*, 2000; Swatek *et al*, 2019) was added and Ub was cleaved from the beads for 16 h at 37°C. The supernatant was removed, the beads were washed with Lb^{pro} reaction buffer, and the supernatants pooled and acidified to pH < 4 using formic acid (FA), prior to fractionation using StageTips (Rappsilber *et al*, 2007). StageTips were assembled using 4 plugs that were cut using a gauge 16 needle (Hamilton) from C_4 substrate (SPE-Disks-Bio-C4-300.47.20, AffiniSEP) and assembled into a P200 pipette tip using a plunger (Hamilton). The matrix was activated by the addition of 30 μl methanol and the tip was centrifuged inside a 2 ml Eppendorf tube at 800 × *g* for 30 s at room temperature to allow the liquid to pass through. The tip was then equilibrated by passing through 30 μl 80% (v/v) acetonitrile (ACN), 0.1% (v/v) FA, followed by 30 μl 0.1% (v/v) FA. The acidified sample was loaded and centrifuged as above until almost all the liquid had passed through. The tip was then desalted by passing through 40 μl 0.1% (v/v) FA, twice. The StageTip was then washed twice with 30 μl 20% (v/v) ACN, 0.1% (v/v) FA, then the ubiquitin was eluted into a clean tube with two elutions of 30 μl 45% (v/v) ACN, 0.1% (v/v) FA. The eluate was lyophilised and resuspended in Trypsin Resuspension Buffer (Promega) supplemented with Tris–pH 8.0 to ensure a final pH above 6. Sequencing grade modified Trypsin (Promega) was added at a concentration of 1 μg per 250 μg initial

©2022 The Authors

mitochondrial protein, and the samples were incubated for 16 h at 37°C. For StageTip purification after trypsin treatment, the sample was acidified to pH < 4 using FA. AQUA peptides, supplied by Cambridge Research Biochemicals (pS65-Ub peptide) and Cell Signalling Technologies (all other peptides), were spiked at concentrations as indicated in Dataset EV1 and the sample was loaded into a StageTip containing 4 plugs of C_{18} substrate (SPE-Disks-Bio-C18-100.47.20, AffiniSEP) that had been assembled, activated and pre-equilibrated as above. The tip was washed 3 times in 0.1% (v/v) FA, then elution was performed twice with 30 µl 80% (v/v) ACN, 0.1% (v/v) FA. Samples were lyophilised and resuspended in 5% (v/v) ACN, 0.1% (v/v) FA, and 10 µl was injected onto a Dionex Ultimate 3000 HPLC system (Thermo Fisher Scientific) and trapped on a C18Acclaim PepMap100 (5 µm, 100 µm × 20 mm nanoViper; Thermo Scientific). Peptides were eluted with a 60-min acetonitrile gradient (2–40%) at a flow rate of 0.3 µl/min. The analytical column outlet was directly interfaced via an EASY-Spray electrospray ionisation source to a Q Exactive mass spectrometer (Thermo Fisher Scientific). The following settings were used: resolution, 140,000; AGC target, 3E6; maximum injection time, 200 ms; scan range, 150–2,000 m/z. Absolute abundances of Ub peptides were calculated by peak integration using Xcalibur Qual Browser (Version 2.2, Thermo Fisher Scientific). Layouts were applied according to Dataset EV1, and abundances were calculated relative to the known amount of added AQUA reference peptide using Microsoft Excel.

For the detection of pS65-Ub in young flies (Fig 1B), the following modifications to the method were performed. Instead of TUBE-mediated Ub pulldown, mitochondrial fractions were sodium carbonate-extracted to enrich ubiquitinated integral membrane proteins as previously described (Swatek *et al*, 2019). In brief, 4 mg mitochondria were resuspended in 4 ml 100 mM Na_2CO_3. The mixture was incubated for 30 min on ice with occasional vortexing and then centrifuged for 30 min, 21,000 × *g*, 4°C. The supernatant, containing soluble and peripheral membrane proteins, was discarded, and the pellet, containing integral membrane proteins, was then resuspended in Lb^{pro} reaction buffer (1 µl per 10 µg mitochondria). An equal volume of 20 µM Lb^{pro} was added (10 µM final concentration) and the mixture was incubated overnight at 37°C. The samples were centrifuged for 30 min, 21,000 × *g*, 4°C, and the supernatant was acidified and purified using StageTips as above (1 StageTip per 1 mg starting material). Trypsin treatment was performed as above and AQUA peptides were spiked in according to Dataset EV1. Phospho-peptide enrichment was then performed using the High-Select™ TiO_2 Phospho-peptide Enrichment kit (Thermo Fisher Scientific). Each replicate was divided between two TiO_2 columns and prepared according to the manufacturer's instructions. The eluates were pooled, lyophilised and analysed by LC–MS as above.

Antibodies and dyes

The following mouse antibodies were used for immunoblotting (WB) and/or immunofluorescence (IF) in this study: ATP5A (Abcam, ab14748, 1:10,000 (WB), 1:300 (IF, adult muscle)), Ubiquitin (clone FK2, MBL, D058-3, 1:2,000 (WB), 1:250 (IF, adult muscle)), Actin (Millipore, MAB1501, 1:1,000 (WB)), GAPDH (GeneTex, GTX627408, 1:1,000 (WB)). The following rabbit antibodies were used in this study: pS65-Ub (Cell Signalling Technologies, 62802S, 1:750 (WB), 1:200 (IF, larval muscle), 1:250 (IF, adult muscle)), COXIV and SDHA (both kind gifts from Edward Owusu-Ansah (Murari *et al*, 2020), both 1:2,000 (WB)), Porin (Calbiochem, PC548, 1:5,000 (WB)). The following secondary antibodies were used: sheep anti-mouse (HRP-conjugated, GE Healthcare, NXA931V, 1:10,000 (WB)), donkey anti-rabbit (HRP-conjugated, GE Healthcare, NA934V, 1:10,000 (WB)), goat anti-mouse (AlexaFluor 488, Invitrogen, A11001, 1:200 (IF)), goat anti-rabbit (AlexaFluor 594, Invitrogen, A11012, 1:200 (IF)).

Whole-animal lysis and immunoblotting

For the analysis of pS65-Ub levels in whole cell lysates by immunoblot, 180 µl cold RIPA buffer (150 mM NaCl, 1% (v/v) NP-40, 0.5% (w/v) sodium deoxycholate, 0.1% (w/v) SDS, 50 mM Tris–pH 7.4), supplemented with cOmplete and PhosSTOP inhibitors, was added to 2 ml tubes containing 1.4 mm ceramic beads (Fisherbrand 15555799). Animals (5 to 20 per replicate) were harvested and stored on ice or flash-frozen in liquid N_2, with all direct comparisons performed with flies that were harvested in the same manner. The flies were added to the tubes containing RIPA buffer and lysed using a Minilys homogeniser (Bertin Instruments) with the following settings: maximum speed, 10 s on, 10 s on ice, for a total of three cycles. After lysis, samples were returned to ice for 10 min and then centrifuged for 5 min at 21,000 × *g*, 4°C. Ninety microliter supernatant was transferred to a fresh Eppendorf tube and centrifuged a further 10 min at 21,000 × *g*. Fifty microliter supernatant was then transferred to a fresh Eppendorf tube and the protein content determined by BCA assay as above. Thirty microgram total protein was then diluted in NuPAGE LDS loading dye (Invitrogen) and analysed by SDS–PAGE using Novex 4–12% Bis–Tris NuPAGE gels (Invitrogen). For the analysis of mitochondria-enriched fractions, 30–50 µg mitochondrial protein was aliquoted into a tube, centrifuged for 10 min at 16,000 × *g*, the supernatant removed and the pellet resuspended in LDS loading dye prior to SDS–PAGE analysis as above. Gels were transferred onto precut and -soaked PVDF membranes (1704157, BioRad) using the BioRad Transblot Turbo transfer system, and blots were immediately stained with Revert total protein stain (926-11011, LiCOR) or No-Stain total protein (A44449, Invitrogen) where indicated, according to the manufacturer's instructions. Fluorescence intensity was measured using a BioRad Chemidoc MP using the IR680 setting. Blots were then washed by gentle shaking 3 times for 5 min in PBS containing 0.1% (v/v) Tween-20 (PBST) and blocked by incubation with PBST containing 3% (w/v) BSA for 1 h. Blots were washed a further 3 times as above and then incubated at 4°C overnight with primary antibodies in PBST containing 3% (w/v) BSA. Further three washes were performed, then the blots were incubated for 1 h in secondary antibodies made up of PBST containing 3% (w/v) BSA. Blots were then washed twice in PBST and once in PBS (twice in the case of pS65-Ub blots) prior to incubation with the ECL reagent. For pS65-Ub blots, SuperSignal Femto reagent (Thermo Scientific) was used, while other blots used Clarity ECL reagent (BioRad). Blots were imaged using the BioRad Chemidoc MP using exposure settings to minimise overexposure, except where high exposure is indicated. Image analysis was performed using Image Lab (Version 5.2.1 build 11, BioRad) and images were exported as TIFF files for presentation.

©2022 The Authors

Immunostaining of *Drosophila* tissues

Larval fillet and adult thoraces dissections were performed in PBS and fixed in 4% formaldehyde, pH 7.0, for 20 (larval fillet) or 30 (adult flight muscles) minutes, respectively. Permeabilisation was performed for 30 min in PBS containing 0.3% (v/v) Triton X-100 (PBS-TX), then tissues were blocked for 1 h in PBS-TX containing 1% (w/v) BSA (larval fillet) or 4% Horse Serum (adult flight muscles). Primary antibody incubation was performed overnight at 4°C in PBS-TX containing 1% (w/v) BSA. The tissues were washed 3 times for 10 min each in PBS-TX prior to incubation with secondary antibodies for 2 h at room temperature (larval fillet) or overnight at 4°C (adult thoraces). The tissues were washed three times for 10 min in PBS-TX, then once for 10 min in PBS, and rinsed once in water prior to mounting in Prolong Diamond Antifade mounting media with DAPI (Thermo Fisher Scientific).

Microscopy and image analysis

Fluorescence microscopy imaging was performed using a Zeiss LSM 880 confocal microscope equipped with a 20× Plan Apochromat (air, NA 0.8), 63× Plan Apochromat (oil immersion, NA 1.4) and 100× Plan Apochromat (oil immersion, NA 1.4), objective lenses. Laser power and gain settings were adjusted depending on the fluorophore but were maintained across samples for the purpose of comparing pS65-Ub levels among genotypes.

For the quantification of pS65-Ub puncta in adult indirect flight muscles (Fig 3), images were processed using FIJI (ImageJ, Version: 2.3.0/1.53q): A ROI was generated for each of the large pS65-Ub puncta using the threshold tool (Huang method) to generate a mask of the pS65-Ub signal. Puncta smaller than 0.5 μm^2 were removed. The pS65-Ub mask was then applied to the ATP5A staining. The ATP5 immunostaining intensity was extracted and represented for inside and outside pS65-Ub structures.

For the quantification of pS65-Ub puncta in larval muscle 6–7 (Fig 6), images were processed using FIJI (ImageJ, Version 2.1.0/1.53c): z-stacks (5 per image, 0.28 μm step size) were cropped to remove extraneous tissue, axons and neuromuscular junctions, which we observed contained high background signal with the anti-pS65-Ub antibody. The retained area was measured, local background subtraction was performed, and the number of puncta was quantified using the 3D Object Counter v2.0 programme on FIJI using the same threshold value for all samples. Puncta with a size smaller than 0.1 μm^3 were excluded, and the remaining puncta were considered to be true pS65-Ub puncta. The data were imported into Prism, and a frequency distribution analysis was performed to obtain the number and mean volume of puncta.

Immuno-electron microscopy

Thoraces were fixed in 0.1% glutaraldehyde and 4% paraformaldehyde in 0.1 M cacodylate buffer overnight at 4°C. After washing with 0.1 M cacodylate buffer, the samples were stained with 1% uranyl acetate for 1 h at 4°C. After washing with ddH_2O, the samples were dehydrated in a gradient series using 30, 50, 70, 95 and 100% ethanol solutions at −20°C. The samples were then infiltrated with an LR-gold series and mounted in pure LR-gold (Agar Scientific, AGR1284). After polymerisation by UV crosslinking, 90 nm sections were prepared and transformed into a block with 5% normal goat serum (NGS) for 1 h, before incubation with primary antibodies overnight at 4°C. After washing with 1% NGS, samples were incubated with 12 nm colloidal gold anti-rabbit secondary antibodies for 30 min at room temperature (Jackson ImmunoResearch, 111-205-144). Samples were again washed with 1% NGS, and the sections were stained with 4% uranyl acetate and lead citrate before examination by TEM (Tecnai G2 Spirit TWIN transmission electron microscope, Thermo, USA).

Statistical analysis

Statistical analyses were performed using Prism (Version 9.1.0 (216)). For the analysis of mass spectrometry data presented in Fig 2, each Ub modification was analysed by ordinary one-way ANOVA with the Šidák's correction for multiple comparisons (wild-type untreated compared with *Pink1*$^{-}$ and *park*$^{-/-}$ untreated and +/− paraquat comparison within each genotype, five comparisons total). The pS65-Ub abundance presented in Fig 7C was analysed by ordinary one-way ANOVA with the Dunnett's correction for multiple comparisons (each genotype compared with *Atg5*$^{-}$, two comparisons total). For the analysis of the number of pS65-Ub puncta in Fig 6H, data were first log-transformed ($Y = \mathrm{Ln}(y + 1)$) to account for heteroscedasticity in the raw data. The transformed data, as well as the raw data in Fig 6I, were analysed by one-way ANOVA with the Dunnett's multiple comparisons.

Data availability

This study includes no data deposited in external repositories. All data generated or analysed during this study are included in the manuscript and supporting files.

Expanded View for this article is available online.

Acknowledgements

This work is supported by Medical Research Council core funding (MC_UU_00015/6 and MC_UU_00028/6) and MOST-110-2311-B-400-002-MY3. JLU was supported by a Gates Cambridge Scholarship. The funders had no role in study design, data collection and analysis, decision to publish or preparation of the manuscript. Stocks were obtained from the Vienna *Drosophila* Stock Centre and the Bloomington *Drosophila* Stock Center, which is supported by grant National Institutes of Health P40OD018537. We thank Prof. D. Komander for reagents and for support in the early phase of this project, Prof. G Juhasz for generously sharing fly stocks and Whitworth lab members for discussions and critical reading of the manuscript.

Author contributions

Joanne L Usher: Conceptualization; data curation; formal analysis; validation; methodology; writing – original draft; writing – review and editing. **Alvaro Sanchez-Martinez:** Formal analysis; visualization; methodology; writing – review and editing. **Ana Terriente-Felix:** Formal analysis; methodology; writing – review and editing. **Po-Lin Chen:** Formal analysis; visualization; methodology. **Juliette J Lee:** Formal analysis; methodology; writing – review and editing. **Chun-Hong Chen:** Supervision; funding acquisition; investigation; writing – review and editing. **Alexander J Whitworth:** Conceptualization;

©2022 The Authors

formal analysis; supervision; funding acquisition; investigation; writing – original draft; project administration; writing – review and editing.

Disclosure and competing interests statement

The authors declare that they have no conflict of interest.

References

Andreazza S, Samstag CL, Sanchez-Martinez A, Fernandez-Vizarra E, Gomez-Duran A, Lee JJ, Tufi R, Hipp MJ, Schmidt EK, Nicholls TJ *et al* (2019) Mitochondrially-targeted APOBEC1 is a potent mtDNA mutator affecting mitochondrial function and organismal fitness in *Drosophila*. *Nat Commun* 10: 3280

Boyle KB, Ravenhill BJ, Randow F (2019) CALCOCO2/NDP52 initiates selective autophagy through recruitment of ULK and TBK1 kinase complexes. *Autophagy* 15: 1655 – 1656

Burman JL, Pickles S, Wang C, Sekine S, Vargas JNS, Zhang Z, Youle AM, Nezich CL, Wu X, Hammer JA *et al* (2017) Mitochondrial fission facilitates the selective mitophagy of protein aggregates. *J Cell Biol* 216: 3231 – 3247

Clark IE, Dodson MW, Jiang C, Cao JH, Huh JR, Seol JH, Yoo SJ, Hay BA, Guo M (2006) *Drosophila* pink1 is required for mitochondrial function and interacts genetically with parkin. *Nature* 441: 1162 – 1166

Cornelissen T, Vilain S, Vints K, Gounko N, Verstreken P, Vandenberghe W (2018) Deficiency of parkin and PINK1 impairs age-dependent mitophagy in *Drosophila*. *Elife* 7: e35878

Cunningham CN, Baughman JM, Phu L, Tea JS, Yu C, Coons M, Kirkpatrick DS, Bingol B, Corn JE (2015) USP30 and parkin homeostatically regulate atypical ubiquitin chains on mitochondria. *Nat Cell Biol* 17: 160 – 169

Fiil BK, Damgaard RB, Wagner SA, Keusekotten K, Fritsch M, Bekker-Jensen S, Mailand N, Choudhary C, Komander D, Gyrd-Hansen M (2013) OTULIN restricts Met1-linked ubiquitination to control innate immune signaling. *Mol Cell* 50: 818 – 830

Filograna R, Godena VK, Sanchez-Martinez A, Ferrari E, Casella L, Beltramini M, Bubacco L, Whitworth AJ, Bisaglia M (2016) Superoxide dismutase (SOD)-mimetic M40403 is protective in cell and Fly models of Paraquat toxicity: implications for Parkinson disease. *J Biol Chem* 291: 9257 – 9267

Gan ZY, Callegari S, Cobbold SA, Cotton TR, Mlodzianoski MJ, Schubert AF, Geoghegan ND, Rogers KL, Leis A, Dewson G *et al* (2022) Activation mechanism of PINK1. *Nature* 602: 328 – 335

GBD (2018) Global, regional, and national burden of Parkinson's disease, 1990–2016: a systematic analysis for the global burden of disease study 2016. *Lancet Neurol* 17: 939 – 953

Gersch M, Gladkova C, Schubert AF, Michel MA, Maslen S, Komander D (2017) Mechanism and regulation of the Lys6-selective deubiquitinase USP30. *Nat Struct Mol Biol* 24: 920 – 930

Gladkova C, Maslen SL, Skehel JM, Komander D (2018) Mechanism of parkin activation by PINK1. *Nature* 559: 410 – 414

Greene JC, Whitworth AJ, Kuo I, Andrews LA, Feany MB, Pallanck LJ (2003) Mitochondrial pathology and apoptotic muscle degeneration in drosophila parkin mutants. *Proc Natl Acad Sci USA* 100: 4078 – 4083

Guarne A, Hampoelz B, Glaser W, Carpena X, Tormo J, Fita I, Skern T (2000) Structural and biochemical features distinguish the foot-and-mouth disease virus leader proteinase from other papain-like enzymes. *J Mol Biol* 302: 1227 – 1240

Heo JM, Ordureau A, Paulo JA, Rinehart J, Harper JW (2015) The PINK1-PARKIN mitochondrial Ubiquitylation pathway drives a program of OPTN/NDP52 recruitment and TBK1 activation to promote Mitophagy. *Mol Cell* 60: 7 – 20

Hjerpe R, Aillet F, Lopitz-Otsoa F, Lang V, England P, Rodriguez MS (2009) Efficient protection and isolation of ubiquitylated proteins using tandem ubiquitin-binding entities. *EMBO Rep* 10: 1250 – 1258

Hospenthal MK, Mevissen TET, Komander D (2015) Deubiquitinase-based analysis of ubiquitin chain architecture using ubiquitin chain restriction (UbiCRest). *Nat Protoc* 10: 349 – 361

Hou X, Fiesel FC, Truban D, Castanedes Casey M, Lin WL, Soto AI, Tacik P, Rousseau LG, Diehl NN, Heckman MG *et al* (2018) Age- and disease-dependent increase of the mitophagy marker phospho-ubiquitin in normal aging and Lewy body disease. *Autophagy* 14: 1404 – 1418

Hrdinka M, Fiil BK, Zucca M, Leske D, Bagola K, Yabal M, Elliott PR, Damgaard RB, Komander D, Jost PJ *et al* (2016) CYLD limits Lys63- and Met1-linked ubiquitin at receptor complexes to regulate innate immune signaling. *Cell Rep* 14: 2846 – 2858

Jacoupy M, Hamon-Keromen E, Ordureau A, Erpapazoglou Z, Coge F, Corvol JC, Nosjean O, Mannoury la Cour C, Millan MJ, Boutin JA *et al* (2019) The PINK1 kinase-driven ubiquitin ligase Parkin promotes mitochondrial protein import through the presequence pathway in living cells. *Sci Rep* 9: 11829

Kane LA, Lazarou M, Fogel AI, Li Y, Yamano K, Sarraf SA, Banerjee S, Youle RJ (2014) PINK1 phosphorylates ubiquitin to activate Parkin E3 ubiquitin ligase activity. *J Cell Biol* 205: 143 – 153

Kazlauskaite A, Kondapalli C, Gourlay R, Campbell DG, Ritorto MS, Hofmann K, Alessi DR, Knebel A, Trost M, Muqit MM (2014) Parkin is activated by PINK1-dependent phosphorylation of ubiquitin at Ser65. *Biochem J* 460: 127 – 139

Kim M, Sandford E, Gatica D, Qiu Y, Liu X, Zheng Y, Schulman BA, Xu J, Semple I, Ro SH *et al* (2016) Mutation in ATG5 reduces autophagy and leads to ataxia with developmental delay. *Elife* 5: e12245

Kim YY, Um JH, Yoon JH, Kim H, Lee DY, Lee YJ, Jee HJ, Kim YM, Jang JS, Jang YG *et al* (2019) Assessment of mitophagy in mt-Keima drosophila revealed an essential role of the PINK1-Parkin pathway in mitophagy induction in vivo. *FASEB J* 33: 9742 – 9751

Kitada T, Asakawa S, Hattori N, Matsumine H, Yamamura Y, Minoshima S, Yokochi M, Mizuno Y, Shimizu N (1998) Mutations in the parkin gene cause autosomal recessive juvenile parkinsonism. *Nature* 392: 605 – 608

Kocaturk NM, Gozuacik D (2018) Crosstalk between mammalian autophagy and the ubiquitin-proteasome system. *Front Cell Dev Biol* 6: 128

Kondapalli C, Kazlauskaite A, Zhang N, Woodroof HI, Campbell DG, Gourlay R, Burchell L, Walden H, Macartney TJ, Deak M *et al* (2012) PINK1 is activated by mitochondrial membrane potential depolarization and stimulates Parkin E3 ligase activity by phosphorylating serine 65. *Open Biol* 2: 120080

Koyano F, Okatsu K, Kosako H, Tamura Y, Go E, Kimura M, Kimura Y, Tsuchiya H, Yoshihara H, Hirokawa T *et al* (2014) Ubiquitin is phosphorylated by PINK1 to activate parkin. *Nature* 510: 162 – 166

Koyano F, Yamano K, Kosako H, Tanaka K, Matsuda N (2019) Parkin recruitment to impaired mitochondria for nonselective ubiquitylation is facilitated by MITOL. *J Biol Chem* 294: 10300 – 10314

Lazarou M, McKenzie M, Ohtake A, Thorburn DR, Ryan MT (2007) Analysis of the assembly profiles for mitochondrial- and nuclear-DNA-encoded subunits into complex I. *Mol Cell Biol* 27: 4228 – 4237

Lazarou M, Sliter DA, Kane LA, Sarraf SA, Wang CX, Burman JL, Sideris DP, Fogel AI, Youle RJ (2015) The ubiquitin kinase PINK1 recruits autophagy receptors to induce mitophagy. *Nature* 524: 309 – 314

 ©2022 The Authors

Lee JJ, Sanchez-Martinez A, Zarate AM, Beninca C, Mayor U, Clague MJ, Whitworth AJ (2018) Basal mitophagy is widespread in *Drosophila* but minimally affected by loss of Pink1 or parkin. *J Cell Biol* 217: 1613 – 1622

Liu S, Lu B (2010) Reduction of protein translation and activation of autophagy protect against PINK1 pathogenesis in *Drosophila melanogaster*. *PLoS Genet* 6: e1001237

Liu YT, Sliter DA, Shammas MK, Huang X, Wang C, Calvelli H, Maric DS, Narendra DP (2021) Mt-Keima detects PINK1-PRKN mitophagy in vivo with greater sensitivity than Mito-QC. *Autophagy* 17: 3753 – 3762

Martinez A, Lectez B, Ramirez J, Popp O, Sutherland JD, Urbe S, Dittmar G, Clague MJ, Mayor U (2017) Quantitative proteomic analysis of Parkin substrates in *Drosophila* neurons. *Mol Neurodegener* 12: 29

McLelland G-L, Soubannier V, Chen CX, McBride HM, Fon EA (2014) Parkin and PINK1 function in a vesicular trafficking pathway regulating mitochondrial quality control. *EMBO J* 33: 282 – 295

McLelland GL, Goiran T, Yi W, Dorval G, Chen CX, Lauinger ND, Krahn AI, Valimehr S, Rakovic A, Rouiller I *et al* (2018) Mfn2 ubiquitination by PINK1/parkin gates the p97-dependent release of ER from mitochondria to drive mitophagy. *Elife* 7: e32866

McWilliams TG, Prescott AR, Montava-Garriga L, Ball G, Singh F, Barini E, Muqit MMK, Brooks SP, Ganley IG (2018) Basal Mitophagy occurs independently of PINK1 in mouse tissues of high metabolic demand. *Cell Metab* 27: 439 – 449

Murari A, Rhooms SK, Goparaju NS, Villanueva M, Owusu-Ansah E (2020) An antibody toolbox to track complex I assembly defines AIF's mitochondrial function. *J Cell Biol* 219: e202001071

Narendra D, Tanaka A, Suen DF, Youle RJ (2008) Parkin is recruited selectively to impaired mitochondria and promotes their autophagy. *J Cell Biol* 183: 795 – 803

Nguyen TN, Padman BS, Usher J, Oorschot V, Ramm G, Lazarou M (2016) Atg8 family LC3/GABARAP proteins are crucial for autophagosome-lysosome fusion but not autophagosome formation during PINK1/Parkin mitophagy and starvation. *J Cell Biol* 215: 857 – 874

Okatsu K, Kimura M, Oka T, Tanaka K, Matsuda N (2015a) Unconventional PINK1 localization to the outer membrane of depolarized mitochondria drives Parkin recruitment. *J Cell Sci* 128: 964 – 978

Okatsu K, Koyano F, Kimura M, Kosako H, Saeki Y, Tanaka K, Matsuda N (2015b) Phosphorylated ubiquitin chain is the genuine Parkin receptor. *J Cell Biol* 209: 111 – 128

Ordureau A, Heo JM, Duda DM, Paulo JA, Olszewski JL, Yanishevski D, Rinehart J, Schulman BA, Harper JW (2015) Defining roles of PARKIN and ubiquitin phosphorylation by PINK1 in mitochondrial quality control using a ubiquitin replacement strategy. *Proc Natl Acad Sci USA* 112: 6637 – 6642

Ordureau A, Paulo JA, Zhang J, An H, Swatek KN, Cannon JR, Wan Q, Komander D, Harper JW (2020) Global landscape and dynamics of Parkin and USP30-dependent Ubiquitylomes in iNeurons during Mitophagic signaling. *Mol Cell* 77: 1124 – 1142

Ordureau A, Paulo JA, Zhang W, Ahfeldt T, Zhang J, Cohn EF, Hou Z, Heo JM, Rubin LL, Sidhu SS *et al* (2018) Dynamics of PARKIN-dependent mitochondrial Ubiquitylation in induced neurons and model systems revealed by digital snapshot proteomics. *Mol Cell* 70: 211 – 227

Ordureau A, Sarraf SA, Duda DM, Heo JM, Jedrychowski MP, Sviderskiy VO, Olszewski JL, Koerber JT, Xie T, Beausoleil SA *et al* (2014) Quantitative proteomics reveal a feedforward mechanism for mitochondrial PARKIN translocation and ubiquitin chain synthesis. *Mol Cell* 56: 360 – 375

Park J, Lee SB, Lee S, Kim Y, Song S, Kim S, Bae E, Kim J, Shong M, Kim JM *et al* (2006) Mitochondrial dysfunction in *Drosophila* PINK1 mutants is complemented by parkin. *Nature* 441: 1157 – 1161

Poole AC, Thomas RE, Andrews LA, McBride HM, Whitworth AJ, Pallanck LJ (2008) The PINK1/Parkin pathway regulates mitochondrial morphology. *Proc Natl Acad Sci USA* 105: 1638 – 1643

Rappsilber J, Mann M, Ishihama Y (2007) Protocol for micro-purification, enrichment, pre-fractionation and storage of peptides for proteomics using StageTips. *Nat Protoc* 2: 1896 – 1906

Rasool S, Veyron S, Soya N, Eldeeb MA, Lukacs GL, Fon EA, Trempe JF (2022) Mechanism of PINK1 activation by autophosphorylation and insights into assembly on the TOM complex. *Mol Cell* 82: 44 – 59

Ryan TA, Phillips EO, Collier CL, Jb Robinson A, Routledge D, Wood RE, Assar EA, Tumbarello DA (2020) Tollip coordinates Parkin-dependent trafficking of mitochondrial-derived vesicles. *EMBO J* 39: e102539

Sato Y, Okatsu K, Saeki Y, Yamano K, Matsuda N, Kaiho A, Yamagata A, Goto-Ito S, Ishikawa M, Hashimoto Y *et al* (2017) Structural basis for specific cleavage of Lys6-linked polyubiquitin chains by USP30. *Nat Struct Mol Biol* 24: 911 – 919

Sauvé V, Sung G, Soya N, Kozlov G, Blaimschein N, Miotto LS, Trempe JF, Lukacs GL, Gehring K (2018) Mechanism of parkin activation by phosphorylation. *Nat Struct Mol Biol* 25: 623 – 630

Shiba-Fukushima K, Imai Y, Yoshida S, Ishihama Y, Kanao T, Sato S, Hattori N (2012) PINK1-mediated phosphorylation of the Parkin ubiquitin-like domain primes mitochondrial translocation of Parkin and regulates mitophagy. *Sci Rep* 2: 1002

Shiba-Fukushima K, Ishikawa KI, Inoshita T, Izawa N, Takanashi M, Sato S, Onodera O, Akamatsu W, Okano H, Imai Y *et al* (2017) Evidence that phosphorylated ubiquitin signaling is involved in the etiology of Parkinson's disease. *Hum Mol Genet* 26: 3172 – 3185

Stern MB, Lang A, Poewe W (2012) Toward a redefinition of Parkinson's disease. *Mov Disord* 27: 54 – 60

Stevens DA, Lee Y, Kang HC, Lee BD, Lee YI, Bower A, Jiang H, Kang SU, Andrabi SA, Dawson VL *et al* (2015) Parkin loss leads to PARIS-dependent declines in mitochondrial mass and respiration. *Proc Natl Acad Sci USA* 112: 11696 – 11701

Sugiura A, McLelland GL, Fon EA, McBride HM (2014) A new pathway for mitochondrial quality control: mitochondrial-derived vesicles. *EMBO J* 33: 2142 – 2156

Swatek KN, Komander D (2016) Ubiquitin modifications. *Cell Res* 26: 399 – 422

Swatek KN, Usher JL, Kueck AF, Gladkova C, Mevissen TET, Pruneda JN, Skern T, Komander D (2019) Insights into ubiquitin chain architecture using Ub-clipping. *Nature* 572: 533 – 537

Tanaka A, Cleland MM, Xu S, Narendra DP, Suen DF, Karbowski M, Youle RJ (2010) Proteasome and p97 mediate mitophagy and degradation of mitofusins induced by Parkin. *J Cell Biol* 191: 1367 – 1380

Tanner CM, Kamel F, Ross GW, Hoppin JA, Goldman SM, Korell M, Marras C, Bhudhikanok GS, Kasten M, Chade AR *et al* (2011) Rotenone, paraquat, and Parkinson's disease. *Environ Health Perspect* 119: 866 – 872

Valente EM, Abou-Sleiman PM, Caputo V, Muqit MM, Harvey K, Gispert S, Ali Z, Del Turco D, Bentivoglio AR, Healy DG *et al* (2004) Hereditary early-onset Parkinson's disease caused by mutations in PINK1. *Science* 304: 1158 – 1160

Vincow ES, Merrihew G, Thomas RE, Shulman NJ, Beyer RP, MacCoss MJ, Pallanck LJ (2013) The PINK1-Parkin pathway promotes both mitophagy and selective respiratory chain turnover in vivo. *Proc Natl Acad Sci USA* 110: 6400 – 6405

Vives-Bauza C, Zhou C, Huang Y, Cui M, de Vries RL, Kim J, May J, Tocilescu MA, Liu W, Ko HS *et al* (2010) PINK1-dependent recruitment of Parkin to mitochondria in mitophagy. *Proc Natl Acad Sci USA* 107: 378 – 383

Wall CE, Rose CM, Adrian M, Zeng YJ, Kirkpatrick DS, Bingol B (2019) PPEF2 opposes PINK1-mediated mitochondrial quality control by dephosphorylating ubiquitin. *Cell Rep* 29: 3280 – 3292

Wang L, Cho YL, Tang Y, Wang J, Park JE, Wu Y, Wang C, Tong Y, Chawla R, Zhang J *et al* (2018) PTEN-L is a novel protein phosphatase for ubiquitin dephosphorylation to inhibit PINK1-Parkin-mediated mitophagy. *Cell Res* 28: 787 – 802

Watzlawik JO, Hou X, Fricova D, Ramnarine C, Barodia SK, Gendron TF, Heckman MG, DeTure M, Siuda J, Wszolek ZK *et al* (2021) Sensitive ELISA-based detection method for the mitophagy marker p-S65-Ub in human cells, autopsy brain, and blood samples. *Autophagy* 17: 2613 – 2628

Wauer T, Swatek KN, Wagstaff JL, Gladkova C, Pruneda JN, Michel MA, Gersch M, Johnson CM, Freund SM, Komander D (2015) Ubiquitin Ser65 phosphorylation affects ubiquitin structure, chain assembly and hydrolysis. *EMBO J* 34: 307 – 325

Whitworth AJ, Theodore DA, Greene JC, Benes H, Wes PD, Pallanck LJ (2005) Increased glutathione S-transferase activity rescues dopaminergic neuron loss in a drosophila model of Parkinson's disease. *Proc Natl Acad Sci USA* 102: 8024 – 8029

Yamano K, Kikuchi R, Kojima W, Hayashida R, Koyano F, Kawawaki J, Shoda T, Demizu Y, Naito M, Tanaka K *et al* (2020) Critical role of mitochondrial ubiquitination and the OPTN-ATG9A axis in mitophagy. *J Cell Biol* 219: e201912144

Yamano K, Youle RJ (2013) PINK1 is degraded through the N-end rule pathway. *Autophagy* 9: 1758 – 1769

License: This is an open access article under the terms of the Creative Commons Attribution License, which permits use, distribution and reproduction in any medium, provided the original work is properly cited.

Expanded View Figures

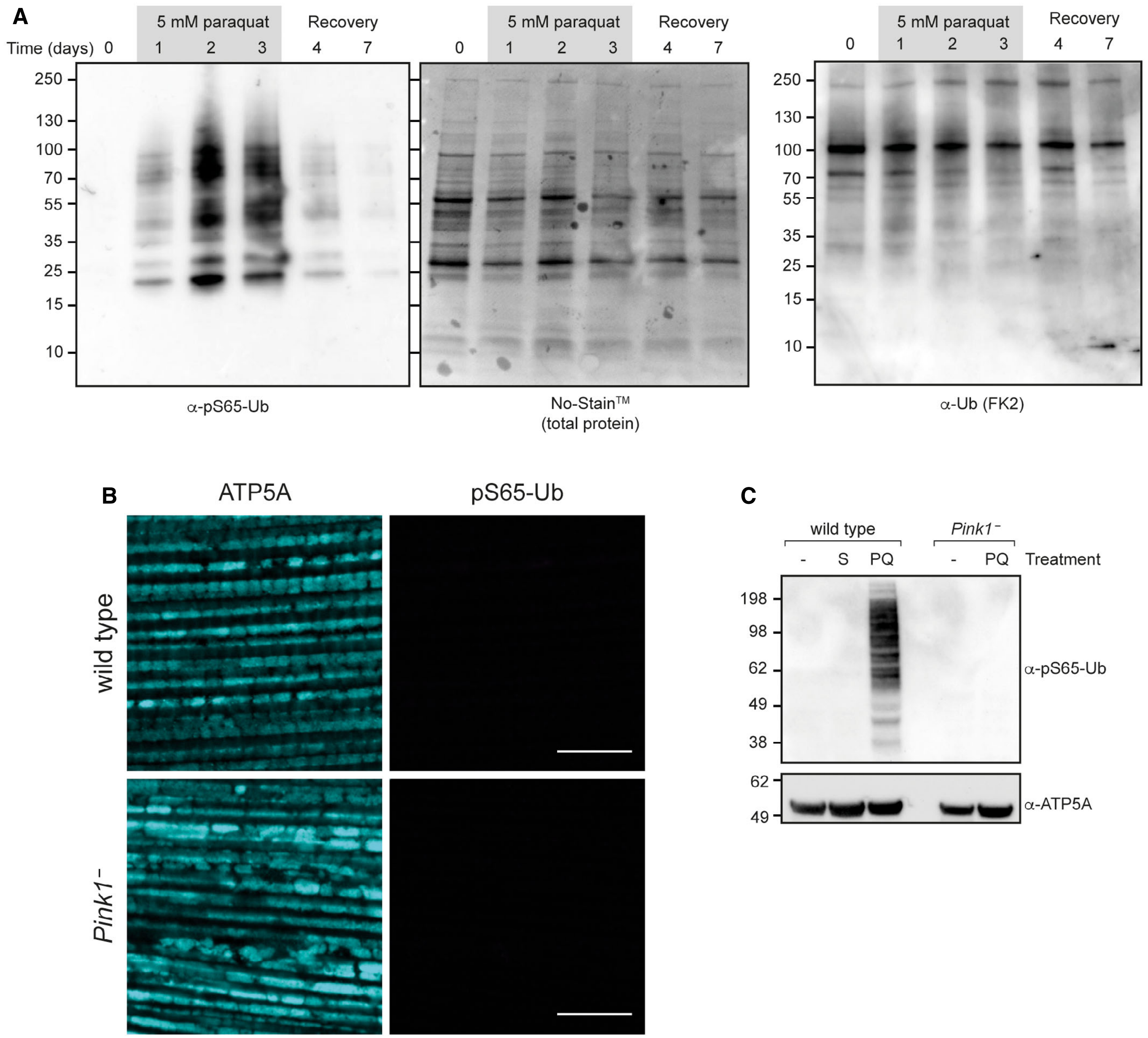

Figure EV1. Dynamics of pS65-Ub production.

A Total protein and immunoblots for the indicated antibodies of mitochondria-enriched fractions from wild-type flies treated for the indicated number of days with paraquat. Recovery, return to normal food.

B Immunostaining of flight muscles of *Pink1*⁻ and wild-type flies. Signal acquisition, brightness and contrast settings for pS65-Ub are identical to those presented in Fig 3D.

C pS65-Ub immunoblot of mitochondrial fractions from wild-type and *Pink1*⁻ flies following either no treatment (–) or after treatment for 3 days with sucrose (S) or paraquat (PQ).

Figure EV2. Loss of rdgC (PPEF2) does not affect pS65-Ub degradation *in vivo*.

Immunoblots for the indicated antibodies of whole-fly lysates treated with paraquat (PQ) followed by recovery on normal food. UT, untreated flies. *LacZ* RNAi serves as control for comparison with knockdown of *rdgC* (the fly homologue of PPEF2). Chart shows the mean +/− SEM of equivalent blots from three biological replicates.

 © 2022 The Authors

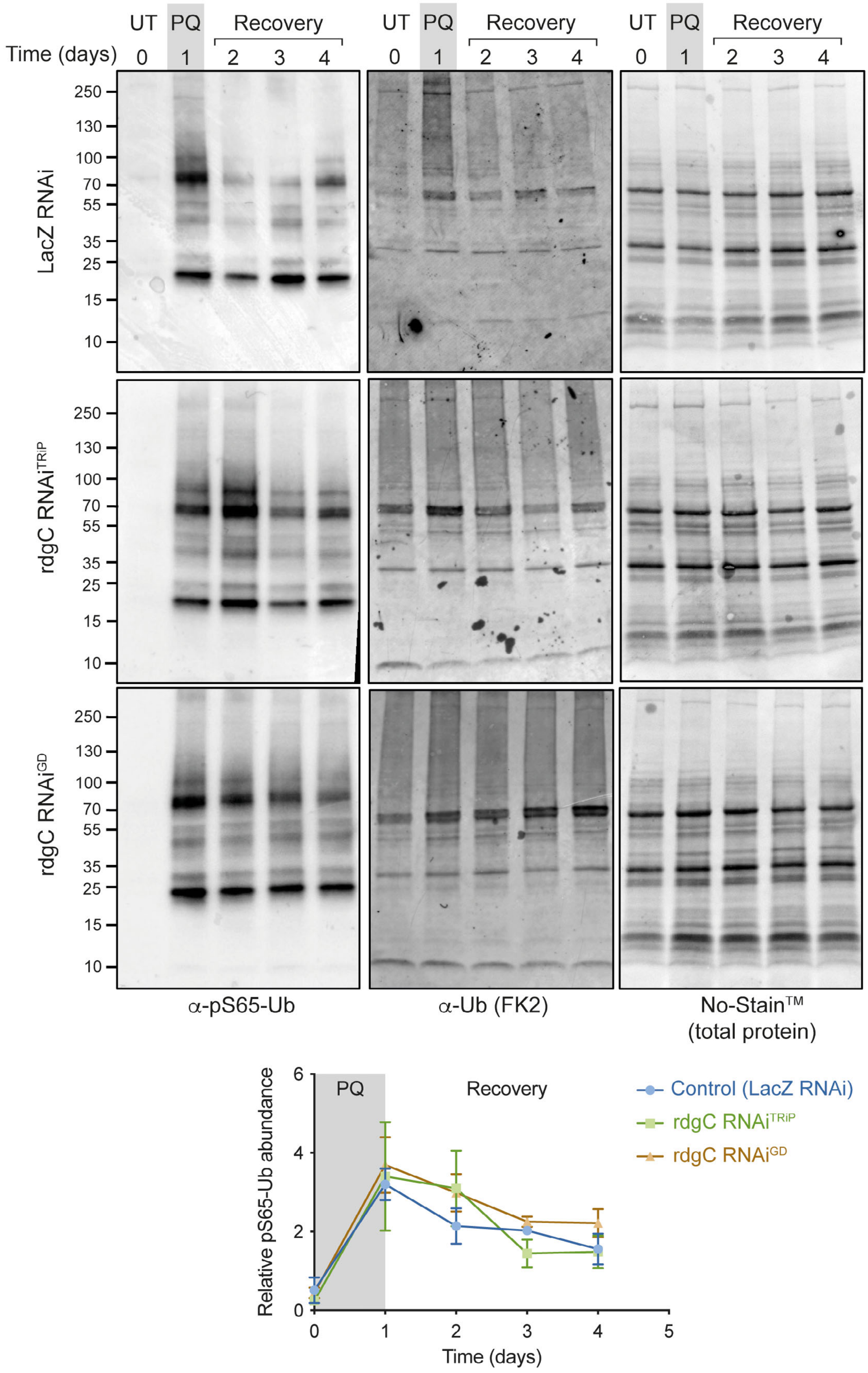
UT
PQ
Recovery
Time (days)
0
1
2
3
4
LacZ RNAi
rdgC RNAi^TRiP
rdgC RNAi^GD
250
130
100
70
55
35
25
15
10
α-pS65-Ub
α-Ub (FK2)
No-Stain™ (total protein)
Relative pS65-Ub abundance
PQ
Recovery
Control (LacZ RNAi)
rdgC RNAi^TRiP
rdgC RNAi^GD
Time (days)

Figure EV2.

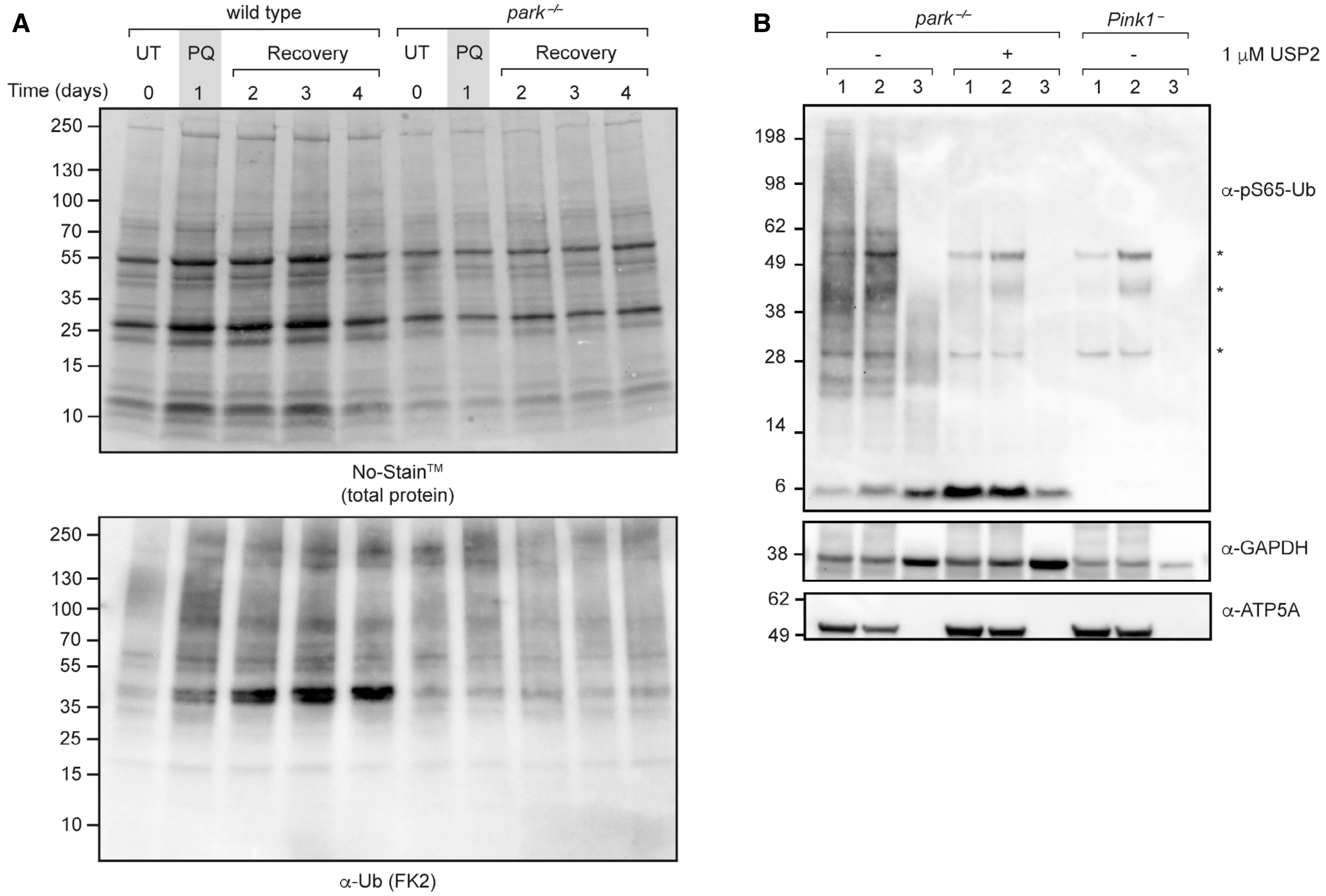

Figure EV3. pS65-Ub accumulates in *park*$^{-/-}$ flies.

A Immunoblots for the indicated antibodies as controls for samples analysed in Fig 2C.

B pS65-Ub immunoblot following subcellular fractionation and USP2 treatment as indicated. (1) 10,000 × *g* pellet; (2) 21,000 × *g* pellet; (3) 21,000 × *g* supernatant. Asterisk (*) denotes nonspecific bands.

 © 2022 The Authors

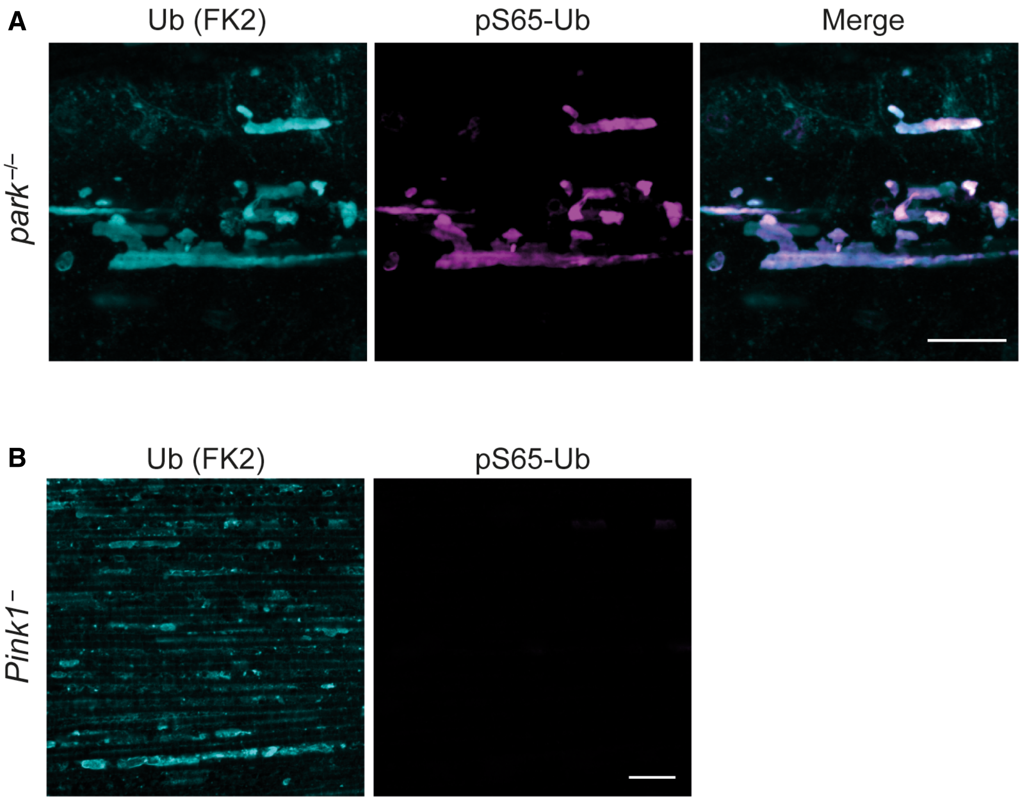

Figure EV4. Pink1-dependent pS65-Ub colocalises with Ub on *park*$^{-/-}$ mitochondria.

A, B Flight muscles from young, untreated (A) *park*$^{-/-}$ and (B) *Pink1*$^{-}$ flies immunostained for conjugated Ub (FK2) and pS65-Ub.

Data information: Scale bars = 10 µm.

Figure EV5. Dynamics of pS65-Ub production/turnover in autophagy mutants.

- A pS65-Ub immunoblot of mitochondrial fractions from wild-type and *Atg5*$^{-}$ flies harvested at the indicated ages. Asterisk (*) denotes nonspecific band.
- B pS65-Ub immunoblotting in whole-animal lysates from wandering L3 larvae of the indicated genotypes.
- C pS65-Ub immunoblot of mitochondrial fractions from wild-type and *Atg5*$^{-}$ flies following a paraquat (PQ) pulse-chase assay. UT, untreated; Recovery, return to normal food.
- D Quantification of pS65-Ub lane densitometry from n = 3 independent replicates of (B), expressed relative to the most intense band in each blot. Charts show mean ± SEM.

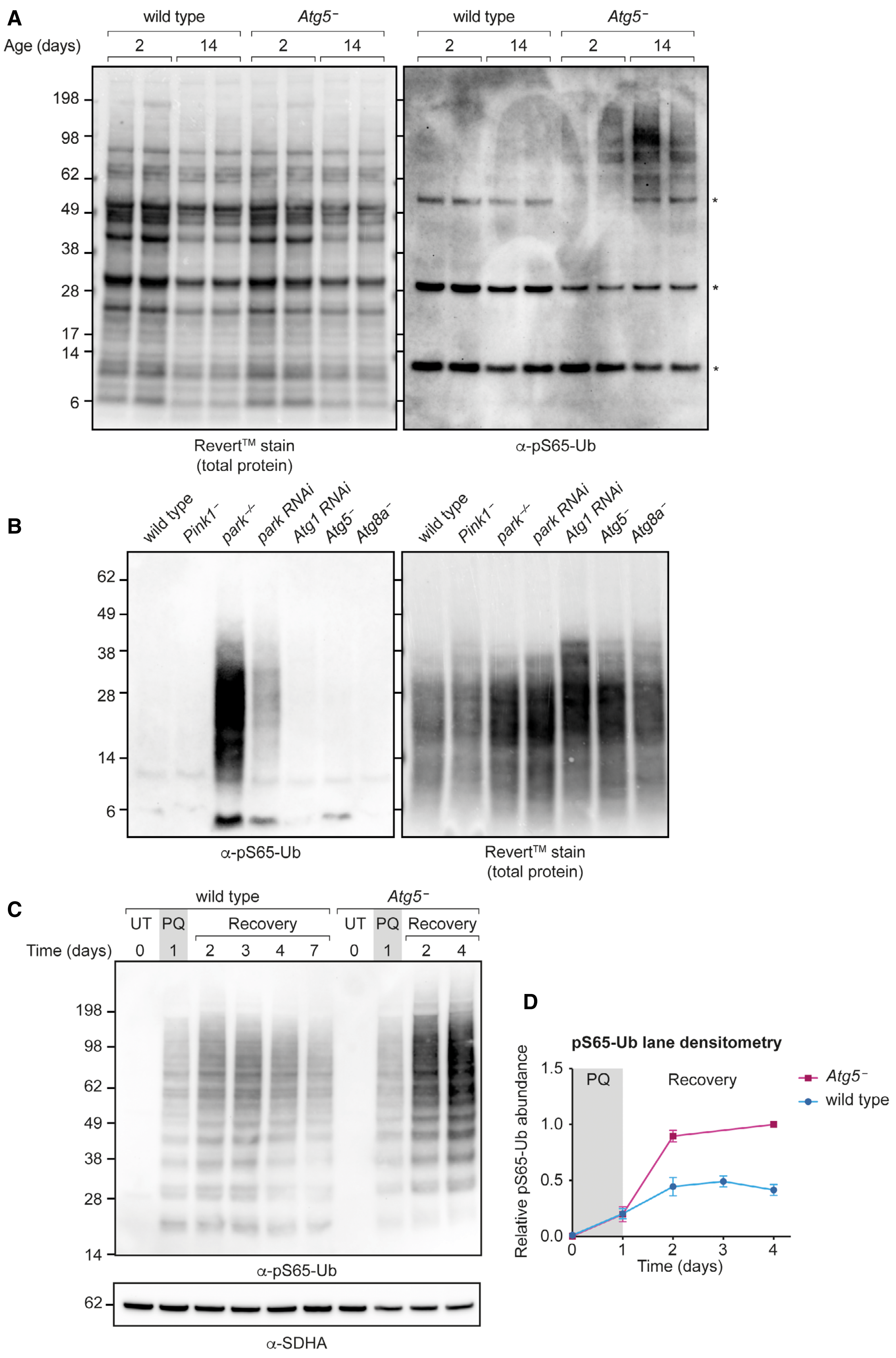

Figure EV5.

© 2022 The Authors

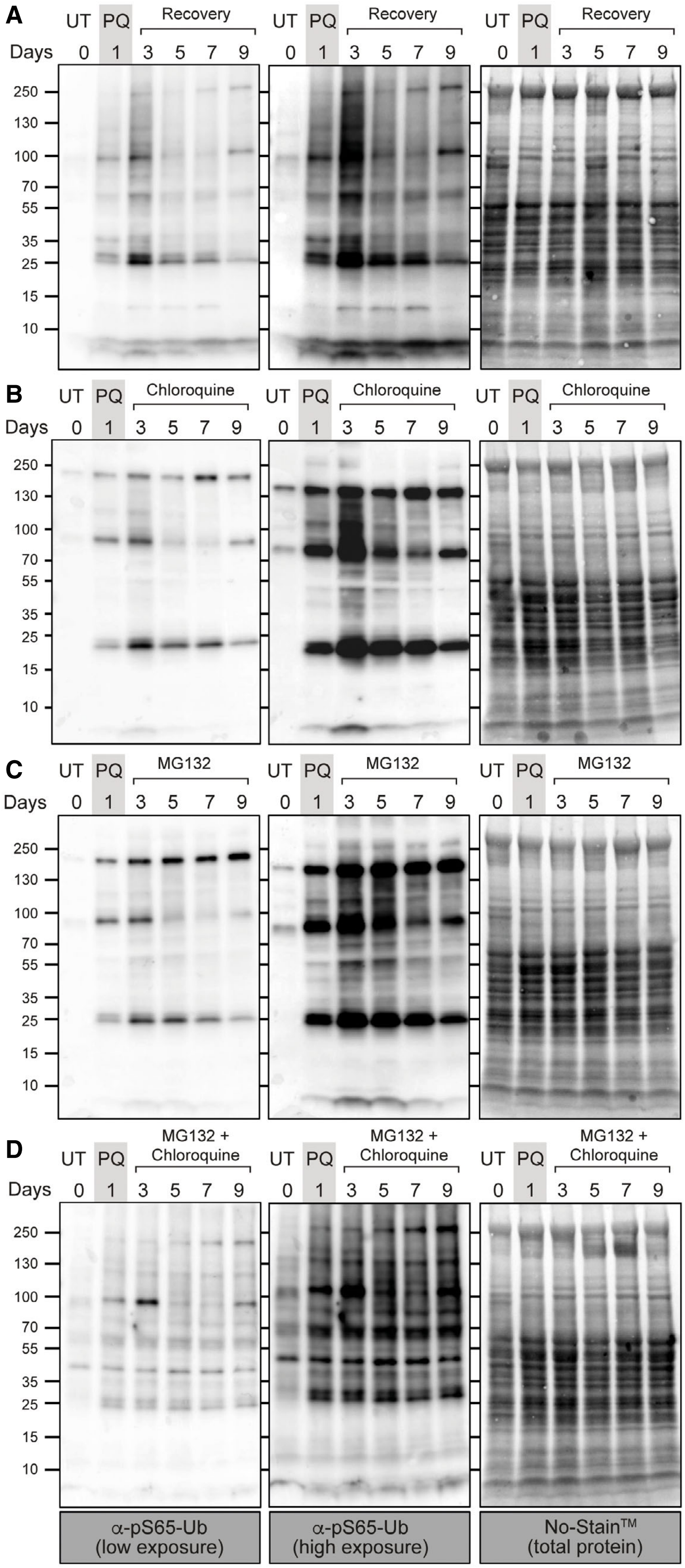

Figure EV6. Impact of lysosome and proteasome inhibitors on pS65-Ub degradation.

A–D pS65-Ub immunoblots of whole-fly lysates treated with paraquat (PQ) followed by recovery on filter papers with sucrose solution only (A) or dosed with (B) chloroquine (2 mM), (C) MG132 (50 μM) or both (D). Blots are representative of duplicate experiments.

© 2022 The Authors
