# Supplementary material for: Parkin drives pS65‐Ub turnover independently of canonical autophagy in Drosophila
Source: EMBO Rep. 2022 Oct 17;23(12):e53552. doi: 10.15252/embr.202153552 (PMC9724668; doi:10.15252/embr.202153552)
Supplement: Supplementary file 4 [file EMBR-23-e53552-s001.pdf]

EMBO reports

Parkin drives pS65-Ub turnover independently of canonical autophagy in Drosophila

Joanne Usher, Alvaro Sanchez-Martinez, Ana Terriente-Felix, Po-Lin Chen, Juliette Lee, Chun-Hong CHEN, and Alexander Whitworth

DOI: 10.15252/embr.202153552

Corresponding author(s): Alexander Whitworth (a.whitworth@mrc-mbu.cam.ac.uk)

Review Timeline:

Submission Date:	30th Jun 21
Editorial Decision:	3rd Aug 21
Revision Received:	7th Jul 22
Editorial Decision:	26th Aug 22
Revision Received:	5th Sep 22
Accepted:	20th Sep 22

Editor: Martina Rembold

Transaction Report:

(Note: With the exception of the correction of typographical or spelling errors that could be a source of ambiguity, letters and reports are not edited. Depending on transfer agreements, referee reports obtained elsewhere may or may not be included in this compilation. Referee reports are anonymous unless the Referee chooses to sign their reports.)

1st Editorial Decision **3rd Aug 2021**

Dear Alex,

Thank you for the submission of your research manuscript to our journal and thank you for your patience while we have further discussed the referee reports (copied below) with the referees and the editorial team.

As you will see, the referee's opinions on the novelty and advance provided are not in agreement. Referee 1 points out that it has been shown before that the Pink1-Parkin pathway promotes other forms of mitochondrial degradation in addition to canonical autophagy. Referee 2 and 3 on the other hand are rather positive. While referee 3 agrees in his/her further feedback that aspects of the study have been shown before, this referee still considers the findings reported of interest and value for the field.

On balance, we have decided to give you the chance to respond to the referee concerns and to revise your study for potential publication in EMBO reports.

Please address the concerns from all three referees, also the first point from referee #2, which I think is feasible and will give a bit more insight into the autophagy-independent mechanisms at play. Moreover, we note that the pS65-Ub antibody has been published before but has not been characterized in depth, as also pointed out by the advisor who commented on your study when it was editorially assessed. The advisor noted: "From a technical point of view, they present a great new tool, but this lacks characterization: there are a number of labeling experiments and westerns, but a thorough analysis of pS65-Ub labeled structures (eg by EM) is not there and thus we don't know what types of structures are detected. " We think that a more detailed characterization of the antibody along the lines suggested is essential for publication here.

Taken together, we would like to invite you to revise your manuscript with the understanding that the referee concerns (as detailed above and in their reports) must be fully addressed and their suggestions taken on board. Please address all referee concerns in a complete point-by-point response. Acceptance of the manuscript will depend on a positive outcome of a second round of review. It is EMBO reports policy to allow a single round of revision only and acceptance or rejection of the manuscript will therefore depend on the completeness of your responses included in the next, final version of the manuscript.

We invite you to submit your manuscript within three months of a request for revision. This would be November 3rd in your case. However, we are aware of the fact that many laboratories are not fully functional due to COVID-19 related shutdowns and we have therefore extended the revision time for all research manuscripts under our scooping protection to allow for the extra time required to address essential experimental issues. Please contact us to discuss the time needed and the revisions further.

***IMPORTANT NOTE: we perform an initial quality control of all revised manuscripts before re-review. Your manuscript will FAIL this control and the handling will be DELAYED if the following APPLIES:

1) A data availability section is missing.
2) Your manuscript contains error bars based on n=2. Please use scatter blots showing the individual datapoints in these cases. The use of statistical tests needs to be justified.

When submitting your revised manuscript, please carefully review the instructions that follow below. Failure to include requested items will delay the evaluation of your revision.***

When submitting your revised manuscript, we will require:

1) a .docx formatted version of the manuscript text (including legends for main figures, EV figures and tables). Please make sure that the changes are highlighted to be clearly visible.

2) individual production quality figure files as .eps, .tif, .jpg (one file per figure).
Please download our Figure Preparation Guidelines (figure preparation pdf) from our Author Guidelines pages https://www.embopress.org/page/journal/14693178/authorguide for more info on how to prepare your figures.

3) a .docx formatted letter INCLUDING the reviewers' reports and your detailed point-by-point responses to their comments. As part of the EMBO Press transparent editorial process, the point-by-point response is part of the Review Process File (RPF), which will be published alongside your paper.

4) a complete author checklist, which you can download from our author guidelines (). Please insert information in the checklist that is also reflected in the manuscript. The completed author checklist will also be part of the RPF.

5) Please note that all corresponding authors are required to supply an ORCID ID for their name upon submission of a revised

manuscript (). Please find instructions on how to link your ORCID ID to your account in our manuscript tracking system in our Author guidelines
()

6) We replaced Supplementary Information with Expanded View (EV) Figures and Tables that are collapsible/expandable online. A maximum of 5 EV Figures can be typeset. EV Figures should be cited as 'Figure EV1, Figure EV2" etc... in the text and their respective legends should be included in the main text after the legends of regular figures.

- For the figures that you do NOT wish to display as Expanded View figures, they should be bundled together with their legends in a single PDF file called *Appendix*, which should start with a short Table of Content. Appendix figures should be referred to in the main text as: "Appendix Figure S1, Appendix Figure S2" etc. See detailed instructions regarding expanded view here:

- Additional Tables/Datasets should be labeled and referred to as Table EV1, Dataset EV1, etc. Legends have to be provided in a separate tab in case of .xls files. Alternatively, the legend can be supplied as a separate text file (README) and zipped together with the Table/Dataset file.

7) Please note that a Data Availability section at the end of Materials and Methods is now mandatory. In case you have no data that requires deposition in a public database, please state so instead of refereeing to the database.
See also < https://www.embopress.org/page/journal/14693178/authorguide#dataavailability>). Please note that the Data Availability Section is restricted to new primary data that are part of this study.

8) We would also encourage you to include the source data for figure panels that show essential data. Numerical data should be provided as individual .xls or .csv files (including a tab describing the data). For blots or microscopy, uncropped images should be submitted (using a zip archive if multiple images need to be supplied for one panel). Additional information on source data and instruction on how to label the files are available .

9) Regarding data quantification
The following points must be specified in each figure legend:
- the name of the statistical test used to generate error bars and P values,
- the number (n) of independent experiments (please specify technical or biological replicates) underlying each data point,
- the nature of the bars and error bars (s.d., s.e.m.)
Discussion of statistical methodology can be reported in the materials and methods section, but figure legends should contain a basic description of n, P and the test applied.
- Please also include scale bars in all microscopy images.

10) Our journal encourages inclusion of *data citations in the reference list* to directly cite datasets that were re-used and obtained from public databases. Data citations in the article text are distinct from normal bibliographical citations and should directly link to the database records from which the data can be accessed. In the main text, data citations are formatted as follows: "Data ref: Smith et al, 2001" or "Data ref: NCBI Sequence Read Archive PRJNA342805, 2017". In the Reference list, data citations must be labeled with "[DATASET]". A data reference must provide the database name, accession number/identifiers and a resolvable link to the landing page from which the data can be accessed at the end of the reference. Further instructions are available at .

11) As part of the EMBO publication's Transparent Editorial Process, EMBO reports publishes online a Review Process File to accompany accepted manuscripts. This File will be published in conjunction with your paper and will include the referee reports, your point-by-point response and all pertinent correspondence relating to the manuscript.

You are able to opt out of this by letting the editorial office know (emboreports@embo.org). If you do opt out, the Review Process File link will point to the following statement: "No Review Process File is available with this article, as the authors have chosen not to make the review process public in this case."

We would also welcome the submission of cover suggestions, or motifs to be used by our Graphics Illustrator in designing a cover.

I look forward to seeing a revised version of your manuscript when it is ready. Please let me know if you have questions or comments regarding the revision.

Kind regards,

Martina

Martina Rembold, PhD

Senior Editor
EMBO reports

****

Referee #1:

The authors have investigated the presence of pSer65-ubiquitin in Drosophila. They found that basal levels of pSer65-ubiquitin in mitochondria were very low in young flies, but significantly increased in older flies. Exposure to paraquat induced a strong increase in the abundance of pSer65-ubiquitin in mitochondria. pSer65-ub formation was abolished in Pink1 -/- flies. Abundance of pSer65-ub was markedly increased in parkin -/- flies. Based on experiments in flies deficient in autophagy proteins Atg1, Atg5 and Atg8a, the authors conclude that he Pink1-parkin pathway promotes mitochondrial turnover independently of canonical autophagy in vivo.

The topic is interesting, but overall the manuscript does not provide novel insights into the role of the Pink1-parkin pathway. It is already well-known that the Pink1-parkin pathway promotes 3 different forms of mitochondrial degradation: 1/ proteasomal degradation of outer mitochondrial membrane proteins; 2/ lysosomal degradation of mitochondrial fragments via mitochondria-derived vesicles; and 3/ what the authors call 'canonical' autophagy. The finding that 'canonical' autophagy does not account for all of the Pink1-parkin-driven mitochondrial degradation is therefore not novel. The fact that Pink1-parkin do not only promote 'canonical' mitophagy but also other forms of mitochondrial degradation, has also already been shown in vivo (e.g. PMID: 23509287).

Other issues:
- In Fig. 1A, the authors show that basal pSer65-ub levels increase significantly during aging, indicating that aging is an important determinant. However, no information is provided on the age of 'mutator' and 'paraquat' flies in Fig. 1A. Crucial information about age of the flies is also missing in Fig. 1C, 1D, 1E, S1A, S2.
- Bar graph in Fig. 1B: it would be interesting to show this result not only for young flies but also for older flies. The single bar is not very informative.
- Fig. 1 D-E: data on total ubiquitin levels should be provided.
- Fig. 1E: the difference between heads and bodies that is reported in the text is not convincing without quantification. The COXIV bands are saturated.
- In the paraquat pulse chase assay (Fig. 2C and other figures), the authors attribute disappearance of the pSer65-ub signal to mitochondrial turnover. However, this could also be due to the action of ubiquitin phosphatases, several of which have been reported (PMID: 29934616, PMID: 31801089).
- Fig. 2C-D and S1A: data on total ubiquitin levels in the mitochondria-enriched fractions are missing.
- The authors downplay the effects of Atg5 depletion on pSer65-ub levels too much. I do not agree with their interpretation that 'canonical mitophagy contributes minimally to the turnover of pS65-Ub-positive structures' (lines 259-260, title and elsewhere). When comparing Fig. 2D with Fig. S3D (paraquat pulse-chase assay), it is clear that the effect of Atg5 depletion on clearance of pSer65-ub is at least as strong as the effect of parkin depletion. Fig. S3A also shows a strong increase in pSer65-ub levels in Atg5- flies.
- The authors detect a striking increase of pSer65-ub in whole cell lysates of parkin-deficient flies (Fig. 3), but not in mitochondria-enriched fractions of parkin-deficient flies (Fig. 2B and S1C). This would suggest that pSer65-ub in parkin-deficient flies accumulates mostly in non-mitochondrial fractions. This is the reverse of what one would expect. The authors do not offer an explanation for this.
- Fig. 5H: the authors should indicate whether the differences between Atg5- and wild type and between Atg8a- and wild type are significant or not.
- Fig. 1SD: the shown effect of USP2 is puzzling because USP2 is known to have very little hydrolyzing activity towards pS65-ub chains (PMID: 25527291).
- Lines 303-305: 'This goes some way to explain why there was such negligible impact of loss of Pink1 or parkin on mitophagy reporters'. This negligible impact has been reported by others to be due to proteasomal degradation of the mito-QC probe (PMID: 32437660, PMID: 33685343).
- Aging is the strongest known risk factor for PD. The link between paraquat exposure and PD is more tenuous. From a PD perspective, it would be more relevant to focus on the pSer65-ub increase observed during normal aging rather than on the paradigm of paraquat intoxication.

Referee #2:

This is an interesting report by Whitworth group that provided evidence that parkin promotes the turnover of phosphorylated S65 ubiquitin (pS65-Ub) without the need to engage classical autophagy. Using Drosophila as the model system, the authors demonstrated that parkin-deficient flies exhibit dramatically enhanced level of pS65-Ub that is over and above its counterparts that are depleted of regulators of canonical autophagy such as Atg1, Atg5 and Atg8a. Moreover, parkin overexpression results

in a reduction of pS65-Ub level in Atg5-deficient flies as well as in control flies treated with paraquat. These novel results certainly help to expand our knowledge on Parkin's function, especially in the context of its in vivo role related to the Parkin/PINK1 pathway.

1. Notwithstanding the above, the mechanism underlying Parkin's effects on pS65-Ub turnover remains unclear. Is the lysosome still required for clearance of pS65-Ub by Parkin in an atg-independent manner? An obvious alternative, as the authors have acknowledged in their discussion, is the proteasomal pathway. Further experiments are needed here to clarify this important question to achieve completeness of the manuscript.

2. Fig. 2: That the increase in pS65-Ub level in paraquat-treated flies is not significantly affected by parkin deficiency is not that surprising as defective mitochondrial clearance in the absence of parkin would mean that mitochondrial-localized full length PINK1 can continue to produce pS65-Ub that would result in an overall increase in pS65Ub level with time, which is consistent with the pulse chase result. Ideally, mass-spectrometry analysis should be performed for this pulse-chase experiment.

3. Fig. 3: It is known that harvested heads still contain a substantial amount of muscle components that could contribute to the weak head-associated pS65-Ub signal observed in Fig. 3C. Isolated brain or removing the proboscis from the fly head represent better approaches to exclude the contamination of muscle-specific signals.

4. Fig. 2B vs Fig. 3: The results in these two figures are a bit confusing. In Fig. 2B, untreated parkin null flies register a modest (non-significant) increase in pS65-Ub level over its untreated control counterparts. In Fig. 3A, the difference between the two groups is dramatic. Given the author's suggestion that "pS65-Ub accumulates on the OMM of dysfunctional mitochondria in the absence of parkin", how would the author reconcile this discrepancy?

5. Related to the above, the drastic difference in pS65-Ub levels between neurons and muscles observed in parkin null flies is both interesting and intriguing. The authors should discuss the significance of this finding as it implies that the brain could degrade pS65-Ub in a parkin-independent manner or that the phosphorylated ubiquitin is hardly generated in the brain of parkin-deficient flies. Notably, the lead author and others have reported dopaminergic neurodegeneration in parkin null flies. It would be interesting to examine whether pS65-Ub is accumulating in a subset of dopaminergic neurons in parkin null flies (via immunohistochemistry) that may not be apparently observed in a whole head lysate preparation.

Minor

- At what age were the animals treated with paraquat? Whenever appropriate, please state the age of the Drosophila in the figures.

- Suppl. Fig. 1C - Why are parkin -/-, parkin OE and mtGFP OE flies exhibiting an overt reduction in pS65-Ub level in the presence of PQ compared to control flies?

Referee #3:

In the present manuscript, Usher and co-workers use the previously developed method of Ub-clipping, combined with powerful fly genetics to gain mechanistic insights on the PINK1-Parkin pathway *in vivo*.
As expected, PINK1 is involved in the generation of Ser65-phosphorylated ubiquitin (pS65-Ub) under normal conditions and after administration of paraquat. Interestingly, Parkin deficient flies have elevated levels of pS65-Ub. In combination with the observation that flies lacking core proteins involved in autophagy (Atg1, Atg5 and Atg8a) do not accumulate pS65-Ub to a similar extent has several implications:
1. mitochondrial turnover *in vivo* is at least partially independent of mitophagy.
2. loss of Parkin causes disrupted flux of pS65-Ub generated by PINK1
3. OMM proteins might be degraded by the proteasome after being tagged by pS65.
In combination with the additional findings:
* synthetic lethality of park deficient flies in combination with an Atg5 or Atg8a deficiency
* presence of pS65-Ub-positive structures in the flight muscles of park-deficient flies
* absence of these in wild-type and Pink1-deficient flies...,
the present manuscripts adds valuable insights on the mitophagy independent functions of the PINK1-Parkin pathway. As this controversy is still not resolved, the data described here is interesting. Overall the manuscript is clearly written, required controls are included, data acquisition/interpretation seems sound and the overall structure is clear.

Mayor issues with the manuscript:
The Ub-clipping for data generation and interpretation thereof is key to understand the presented story. A brief explanation/summary (probably supported by cartoon) of the method might be helpful for readers not familiar with the technique. As EMBO Reports has a broad readership, such a more explanatory section of the method should be included.

Minor points:

Be consistent with writing concerning the PINK1-Parkin (or Pink1-parkin) pathway.
Figure 1 B is not referred in text, should be line 128.
I would remove the allele specification (example: Atg5- (Atg55cc5) from the main text. Specification in material/methods section is enough -> personal preference.
Line 262, "with only a few rare escapers" remove rare -> personal preference.

Editor:

Please address the concerns from all three referees, also the first point from referee #2, which I think is feasible and will give a bit more insight into the autophagy-independent mechanisms at play. Moreover, we note that the pS65-Ub antibody has been published before but has not been characterized in depth, as also pointed out by the advisor who commented on your study when it was editorially assessed. The advisor noted: "From a technical point of view, they present a great new tool, but this lacks characterization: there are a number of labeling experiments and westerns, but a thorough analysis of pS65-Ub labeled structures (eg by EM) is not there and thus we don't know what types of structures are detected. " We think that a more detailed characterization of the antibody along the lines suggested is essential for publication here.

-- We thank the reviewers for their constructive feedback and the Editors for inviting us to revise our manuscript accordingly. We have substantially added to the previous version to (i) add specific data requested by reviewers, (ii) address additional questions relating to the nature and degradation route of pS65-Ub-positive structures. We hope that the reviewers and Editors will appreciate the advancement that this study offers for our understanding of PINK1/Parkin biology *in vivo*.

Referee #1:

The authors have investigated the presence of pSer65-ubiquitin in Drosophila. They found that basal levels of pSer65-ubiquitin in mitochondria were very low in young flies, but significantly increased in older flies. Exposure to paraquat induced a strong increase in the abundance of pSer65-ubiquitin in mitochondria. pSer65-ub formation was abolished in Pink1-/- flies. Abundance of pSer65-ub was markedly increased in parkin-/- flies. Based on experiments in flies deficient in autophagy proteins Atg1, Atg5 and Atg8a, the authors conclude that he Pink1-parkin pathway promotes mitochondrial turnover independently of canonical autophagy in vivo.

The topic is interesting, but overall the manuscript does not provide novel insights into the role of the Pink1-parkin pathway. It is already well-known that the Pink1-parkin pathway promotes 3 different forms of mitochondrial degradation: 1/ proteasomal degradation of outer mitochondrial membrane proteins; 2/ lysosomal degradation of mitochondrial fragments via mitochondria-derived vesicles; and 3/ what the authors call 'canonical' autophagy. The finding that 'canonical' autophagy does not account for all of the Pink1-parkin-driven mitochondrial degradation is therefore not novel. The fact that Pink1-parkin do not only promote 'canonical' mitophagy but also other forms of mitochondrial degradation, has also already been shown in vivo (e.g. PMID: 23509287).

-- We thank the reviewer for their comments on our manuscript, but respectfully disagree with the suggestion that the work is not novel, especially with the inclusion of our revisions. Through our assessment of pS65-Ub levels ***in vivo*** we are able to interrogate the function of the Pink1-parkin pathway in a manner that is entirely orthogonal to previously described methods and not reliant on

the introduction of reporter constructs, which different labs have suggested give different results. We also provide the first *in vivo* evidence that PINK1 phosphorylates Ub specifically on disrupted mitochondria, which has always been assumed but never before confirmed *in vivo*. Further, we would argue that the description of methods to both detect and validate pS65-Ub *in vivo* (through ubiquitin-protease treatment, co-staining with total Ub and comparison with *Pink1* mutants) represent a valuable contribution to the field, for future investigations not just in *Drosophila* but also in other model systems due to the 100% conservation of the Ub sequence and S65 phosphorylation site in mammals.

Other issues:

1. In Fig. 1A, the authors show that basal pSer65-ub levels increase significantly during aging, indicating that aging is an important determinant. However, no information is provided on the age of 'mutator' and 'paraquat' flies in Fig. 1A. Crucial information about age of the flies is also missing in Fig. 1C, 1D, 1E, S1A, S2.

-- We thank the reviewer for raising this important issue and we apologise that this detail was not immediately clear in the original submission. We are very conscious of the impact and importance of age in these experiments and collect all the animals in a standardised and systematic way, paying close attention to their age. We have now added a clear statement on this in the Methods. In essence, unless specifically stated otherwise all animals were 2-3 days old.

2. Bar graph in Fig. 1B: it would be interesting to show this result not only for young flies but also for older flies. The single bar is not very informative.

-- We agree with the reviewer that Fig 1B does not provide the opportunity for a comparison of pS65-Ub amounts between treatments. However, the purpose of this figure was to demonstrate that pS65-Ub is present, although at very low abundance, in young wild-type flies. The experiment required very large sample amounts (in the order of thousands of flies), and could therefore not feasibly be performed on complex genotypes or aged flies, for which up to 50% of the flies may be lost during the ageing process. We therefore point the reviewer to Figure 1A for a comparison of pS65-Ub amounts between young and old flies.

3. Fig. 1D-E: data on total ubiquitin levels should be provided.

-- We appreciate that it can be interesting to know how the levels of total ubiquitin may change under different conditions but for the experiment performed in Fig. 1D-E (sub-cellular fractionation) the levels of total ubiquitin will vary greatly from one fraction to another and as such are not particularly informative. Moreover, we consider that it is the overall abundance of pS65-Ub, i.e., the absolute amount of pS65-Ub not the relative amount pS65-Ub to total Ub, that is relevant to indicate Pink1 activity. Nevertheless, these experiments have been repeated, with equivalent results, to provide data on total Ub.

4. Fig. 1E: the difference between heads and bodies that is reported in the text is not convincing without quantification. The COXIV bands are saturated.

-- As indicated above, these experiments have been repeated to provide new data on total ubiquitin and, in response to the comment here, improving the presentation of the loading control (COXIV), which yielded equivalent results. We intentionally made this a very 'soft' claim ("pS65-Ub levels *appeared* to be greater in heads compared with bodies") and upon replication consider this observation to be upheld. But since it is not sufficiently critical to the current study to warrant extensive quantification, we have softened this statement even further ("…with a possible enrichment in heads" – lines 134-135). If the reviewer feels strongly, we can remove this observation entirely, but we think it is noteworthy enough to retain.

5. In the paraquat pulse chase assay (Fig. 2C and other figures), the authors attribute disappearance of the pSer65-ub signal to mitochondrial turnover. However, this could also be due to the action of ubiquitin phosphatases, several of which have been reported (PMID: 29934616, PMID: 31801089).

-- We thank the reviewer for this interesting suggestion. Investigating the conservation of these phosphatases we found that while PPEF2 (PMID: 31801089) is conserved, PTEN-L (PMID: 29934616) is not. To our knowledge, no other specific phosphatases have been identified to counteract pS65-Ub. We have now evaluated the potential impact of PPEF2 on the turnover of pS65-Ub by performing the PQ pulse-chase assay upon knockdown of the PPEF2 homologue (called rdgC in flies), using two previously described independent transgenic RNAi lines. Results showed that loss of *rdgC/PPEF2* did not significantly affect pS65-Ub turnover. These data are presented in <u>new</u> supplementary Figure EV3 and mentioned in the text, lines 165-168.

6. Fig. 2C-D and S1A: data on total ubiquitin levels in the mitochondria-enriched fractions are missing.

-- Total ubiquitin immunoblots, as well as No-Stain™ for total protein, have now been added as <u>new</u> supplementary Figure EV1C and Figure EV1A, respectively.

7. The authors downplay the effects of Atg5 depletion on pSer65-ub levels too much. I do not agree with their interpretation that 'canonical mitophagy contributes minimally to the turnover of pS65-Ub-positive structures' (lines 259-260, title and elsewhere). When comparing Fig. 2D with Fig. S3D (paraquat pulse-chase assay), it is clear that the effect of Atg5 depletion on clearance of pSer65-ub is at least as strong as the effect of parkin depletion. Fig. S3A also shows a strong increase in pSer65-ub levels in Atg5- flies.

-- We agree with the reviewer, and mention in the text (lines 271-277), that autophagy does clearly contribute to mitochondrial turnover, especially in the context of paraquat treatment, which is likely

to induce a number of different mitochondrial quality control pathways including those that rely on canonical autophagy. The question we are addressing in this manuscript, and we believe is adequately reflected in the text, is whether this is the *major* pathway that is promoted by the Pink1-parkin pathway *in vivo*. The paraquat pulse-chase assays did indeed show a strong accumulation of pS65-Ub upon loss of Atg5, which could either be explained by (i) a requirement for Atg5 in the Pink1/parkin-dependent mitochondrial turnover, or (ii) that mitochondria that would otherwise be turned over by bulk autophagy are being pS65-ubiquitinated in the absence of Atg5 (a likely scenario given that paraquat is a non-selective oxidising agent). Considering the results presented in Figure 7, that parkin overexpression can promote pS65-Ub turnover in the absence of Atg5, we posit that parkin primarily degrades via a mechanism that does not involve Atg5, and the accumulation of pS65-Ub in *Atg5* mutants is a result of failed bulk autophagy. Nevertheless, to reflect the observation in revised supplementary Figure EV4C-D, and to give a more comprehensive assessment of the degradation routes, we have added a comment to the Discussion indicating some contribution from canonical bulk autophagy, lines 370-373.

8. The authors detect a striking increase of pSer65-ub in whole cell lysates of parkin-deficient flies (Fig. 3), but not in mitochondria-enriched fractions of parkin-deficient flies (Fig. 2B and S1C). This would suggest that pSer65-ub in parkin-deficient flies accumulates mostly in non-mitochondrial fractions. This is the reverse of what one would expect. The authors do not offer an explanation for this.

-- We were also intrigued by these observations so we have undertaken a more in-depth analysis of the distribution of pS65-Ub:

We agree that it seems counter-intuitive that the pS65-Ub did not appear to be enriched in “mitochondrial fractions” analysed by mass spectrometry. However, we note that our “mitochondrial fractions” are simply a somewhat crude biochemical fraction (the supernatant from a 1,000x g centrifugation step that was subsequently centrifuged at 10,000x g, with the pellet enriched for mitochondria). Whether a given structure will enrich in the “mitochondrial fraction” will therefore depend on its size and density. As can be observed in the present study (compare ATP5A staining in Fig. 3D vs Suppl. Fig. 2A), and has been previously reported many times (e.g., PMID: 12642658, 18799731, 32047033), mitochondrial morphology and integrity are grossly disturbed in *parkin* mutant flies. With hindsight, we are now mindful that this is likely to have some impact on their physical characteristics and therefore at which g-force the mitochondria would pellet. We show here that the pS65-Ub-positive structures are, in general, very large and have a heterogenous morphology (compared to the regular, kidney-bean shaped wild-type organelles). This change in morphology is already indicative of mitochondrial disruption, which is further supported by the consistently poor immunostaining for the critical bioenergetic component ATP5A. Although the loss of ATP5A staining was noted in the original submission, we have now formalised this observation with quantification across multiple biological replicates (new Figure 3E).

Furthermore, we have now gone beyond the limitation of light microscopy, analysing pS65-Ub distribution at the ultrastructural level by immuno-electron microscopy. As expected, wild type and *Pink1*$^{-}$ flight muscle showed very little background staining of pS65-Ub (further validating the antibody/immunostaining approach), however, *park*$^{-/-}$ flies showed abundant mitochondrial pS65-Ub immunostaining (new Figure 4). Interestingly, pS65-Ub was only observed on electrolucent mitochondria with no pS65-Ub staining detected on intact, electron-dense mitochondria (new Figure 4Cii and Cii). These experiments offer valuable new insights into pS65-Ub formation and distribution *in vivo* since, to our knowledge, there is only one report of pS65-Ub ultrastructural distribution (Hou *et al*, 2018) and none in animal models. Together, these data indicate that pS65-Ub accumulates on disrupted mitochondria in *park*$^{-/-}$ mutant flies. We previously considered the possibility that the pS65-Ub-positive structures might be non-mitochondrial, but, in light of this additional evidence, it now seems compelling that pS65-Ub is indeed found predominantly on mitochondria, at least until the process of degradation is initiated. This mechanism remains unresolved currently but is discussed in response to Reviewer 2's, point 1 below.

9. Fig. 5H: the authors should indicate whether the differences between Atg5- and wild type and between Atg8a- and wild type are significant or not.
-- These are significantly different and we have now indicated this on the figure (now Figure 6).

10. Fig. S1D: the shown effect of USP2 is puzzling because USP2 is known to have very little hydrolyzing activity towards pS65-ub chains (PMID: 25527291).
-- We recognise that USP2, along with many DUBs, has reduced activity towards pS65-Ub compared with unphosphorylated Ub. However, while Wauer et al. (PMID: 25527291) used a USP2 concentration of 190 nM, which is 10-fold less than the amount we use here, the authors state that USP2 "cleaved phosphoUb chains at later time points or at higher enzyme concentration" (this observation was discussed further by the authors in the Review Process file for that paper available from EMBO J). Hence, that we observed cleavage of pS65-Ub chains by USP2 at a concentration of 2 uM is consistent with literature and known function of USP2.

11. Lines 303-305: 'This goes some way to explain why there was such negligible impact of loss of Pink1 or parkin on mitophagy reporters'. This negligible impact has been reported by others to be due to proteasomal degradation of the mito-QC probe (PMID: 32437660, PMID: 33685343).
-- We acknowledge that there is still considerable debate over the various sensitivities of the different mitophagy reporters currently available. Since the current study does not provide further clarity on this, we have removed this statement.

12. Aging is the strongest known risk factor for PD. The link between paraquat exposure and PD is more tenuous. From a PD perspective, it would be more relevant to focus on the pSer65-ub increase observed during normal aging rather than on the paradigm of paraquat intoxication.

-- We agree with the reviewer that ageing is undoubtedly the strongest known risk factor for PD. While paraquat exposure clearly accounts for far fewer cases, the epidemiological evidence is compelling. Nevertheless, the current study was designed to be an exploratory study to determine conditions and protocols in which we can detect pS65-Ub, and to apply various manipulations to begin to address the modes of production and degradation, rather than to explore representative models for the PD population. While the paraquat treatment uncovered a particularly striking elevation of pS65-Ub and, importantly, allowed us to conduct the pulse-chase experiments to tease apart the effects of parkin on either production or turnover of pS65-Ub, it was not intended as a model for PD (and is only mentioned in passing as a parkinsonian toxin). The reviewer will appreciate that ageing studies brings with them a substantial time investment, beyond the scope of the current initial study but echoing the reviewer's comment, this will very much be the focus of future work.

Referee #2:

This is an interesting report by Whitworth group that provided evidence that parkin promotes the turnover of phosphorylated S65 ubiquitin (pS65-Ub) without the need to engage classical autophagy. Using Drosophila as the model system, the authors demonstrated that parkin-deficient flies exhibit dramatically enhanced level of pS65-Ub that is over and above its counterparts that are depleted of regulators of canonical autophagy such as Atg1, Atg5 and Atg8a. Moreover, parkin overexpression results in a reduction of pS65-Ub level in Atg5-deficient flies as well as in control flies treated with paraquat. These novel results certainly help to expand our knowledge on Parkin's function, especially in the context of its in vivo role related to the Parkin/PINK1 pathway.

We thank the reviewer for their appreciation of the novelty of this work, particularly in highlighting that the specific advance here is in elucidating the function of the Pink1-parkin pathway *in vivo*.

1. Notwithstanding the above, the mechanism underlying Parkin's effects on pS65-Ub turnover remains unclear. Is the lysosome still required for clearance of pS65-Ub by Parkin in an atg-independent manner? An obvious alternative, as the authors have acknowledged in their discussion, is the proteasomal pathway. Further experiments are needed here to clarify this important question to achieve completeness of the manuscript.

-- We appreciate that this is indeed an important unresolved aspect of the current work but is also a challenging question to gain a definitive answer to *in vivo* since substantially inhibiting either the proteasome or lysosome is lethal to *Drosophila*. Although such a major question would require its own focussed study to fully explore, we have approached this by testing the effects of classic

chemical inhibitors or the lysosome (choloroquine) and proteasome (MG132). Having first established workable conditions for lysosome/proteasome inhibition and applied these to the paraquat pulse-chase assay, we found that, somewhat surprisingly, inhibition of neither lysosome nor proteasome alone had any substantial effect on pS65-Ub degradation. However, their combined inhibition did noticeably interfere with degradation, indicating some partial or redundant contribution from both major turnover mechanisms. These data are presented in new supplementary Figure EV5 and discussed further in the text.

2. Fig. 2: That the increase in pS65-Ub level in paraquat-treated flies is not significantly affected by parkin deficiency is not that surprising as defective mitochondrial clearance in the absence of parkin would mean that mitochondrial-localized full length PINK1 can continue to produce pS65-Ub that would result in an overall increase in pS65Ub level with time, which is consistent with the pulse chase result. Ideally, mass-spectrometry analysis should be performed for this pulse-chase experiment.

-- We appreciate the reviewer's point here but it isn't clear how mass-spectrometry analysis [we assume the reviewer is thinking of pS65-Ub rather than Pink1 levels] would clarify their point. We agree that the failure to degrade the structures with mitochondria-localised Pink1 could well lead to further deposition of pS65-Ub, but we still thought this incongruous with the prevailing view that parkin deposits (p)Ub. To accommodate this view, we have now tempered our response to this observation.

3. Fig. 3: It is known that harvested heads still contain a substantial amount of muscle components that could contribute to the weak head-associated pS65-Ub signal observed in Fig. 3C. Isolated brain or removing the proboscis from the fly head represent better approaches to exclude the contamination of muscle-specific signals.

-- We appreciate this point and have repeated the experiment shown in Fig. 3C, this time removing the proboscis from the head samples as suggested. We observed equivalent results, with a major pS65-Ub signal in bodies and a minor, albeit appreciable, signal in heads (without proboscises). We have replaced the data on Fig 3C with these new data and added comments reflecting this additional step in the text.

4. Fig. 2B vs Fig. 3: The results in these two figures are a bit confusing. In Fig. 2B, untreated parkin null flies register a modest (non-significant) increase in pS65-Ub level over its untreated control counterparts. In Fig. 3A, the difference between the two groups is dramatic. Given the author's suggestion that "pS65-Ub accumulates on the OMM of dysfunctional mitochondria in the absence of parkin", how would the author reconcile this discrepancy?

-- Thanks for raising this point, also mentioned by Reviewer 1 (point 8), for us to clarify. To briefly reiterate: Our "mitochondrial fractions" are a simple biochemical fraction – the supernatant from a

1,000 xg centrifugation step that was subsequently centrifuged at 10,000 xg. This procedure works well to enriched normal, intact mitochondria in the pellet. However, mitochondrial morphology and integrity are grossly disturbed in *park*$^{-/-}$ mutant flies, and we show here that the pS65-Ub-positive structures are heterogeneous and exist in a range of sizes but are, in general, very large. Therefore, with hindsight, we believe that the pS65-Ub labelled mitochondria in *park*$^{-/-}$ mutants do not sediment like normal, intact mitochondria in the fractionation process that is analysed in Fig. 2B. In contrast, the data in Fig. 3 shows analysis of whole animal lysates in which we would expect to detect any and all pS65-Ub.

5. Related to the above, the drastic difference in pS65-Ub levels between neurons and muscles observed in parkin null flies is both interesting and intriguing. The authors should discuss the significance of this finding as it implies that the brain could degrade pS65-Ub in a parkin-independent manner or that the phosphorylated ubiquitin is hardly generated in the brain of parkin-deficient flies. Notably, the lead author and others have reported dopaminergic neurodegeneration in parkin null flies. It would be interesting to examine whether pS65-Ub is accumulating in a subset of dopaminergic neurons in parkin null flies (via immunohistochemistry) that may not be apparently observed in a whole head lysate preparation.

-- We surmised that the major signal coming from muscle likely reflects the greater proportion of the animal being flight muscle than neurons. Coupled with the fact that flight muscles have a very high mitochondrial content and are highly energetic, making it more prone to mitochondrial stress, this is consistent with a major requirement for Pink1-parkin in this tissue and reflected in the major phenotypes observed here. We have made a specific comment in the text to reflect this (lines 209-214). In response to the reviewer's comments, we did assess immunostaining of aged adult brains but in this context the immunohistochemistry was less selective, with substantial background staining even in *Pink1* mutants. This methodology will require considerable optimisation to be able to definitively assess Pink1 activity at a single-neuron level. Nevertheless, the varying results from different tissues do indeed raise the intriguing possibility of alternative degradation mechanisms in the brain which will be the subject of further investigation.

Minor

6. At what age were the animals treated with paraquat? Whenever appropriate, please state the age of the Drosophila in the figures.

-- This is an important point also raised by reviewer 1 and we apologise that this was not clear in the original submission. We have now added a clear statement on this in the Methods. In essence, unless specifically stated otherwise all animals were 2-3 days old.

7. Suppl. Fig. 1C - Why are parkin -/-, parkin OE and mtGFP OE flies exhibiting an overt reduction in pS65-Ub level in the presence of PQ compared to control flies?

-- We thank the reviewer for pointing out the potential for confusion here. The data shown in (old) Supp Fig 1C represented our initial foray into investigating the impact of parkin modulation on pS65-Ub production and turnover. However, these data illustrate only a single time-point (3 days of paraquat exposure). We subsequently rationalised that, since pS65-Ub levels will be affected by both production by Pink1 and possibly parkin (increasing pS65-Ub levels) as well as downstream turnover (decreasing pS65-Ub levels), a pulse-chase assay would give a greater clarity on the kinetics of production and turnover rather than a single time-point. As the results of the pulse-chase assay supersede this single time-point data, we realise that the data in Suppl. Fig. 1C serve no unique purpose so to avoid potential confusion we have decided to remove it.

Referee #3:

In the present manuscript, Usher and co-workers use the previously developed method of Ub-clipping, combined with powerful fly genetics to gain mechanistic insights on the PINK1-Parkin pathway in vivo.

As expected, PINK1 is involved in the generation of Ser65-phosphorylated ubiquitin (pS65-Ub) under normal conditions and after administration of paraquat. Interestingly, Parkin deficient flies have elevated levels of pS65-Ub. In combination with the observation that flies lacking core proteins involved in autophagy (Atg1, Atg5 and Atg8a) do not accumulate pS65-Ub to a similar extent has several implications:

1. mitochondrial turnover in vivo is at least partially independent of mitophagy.
2. loss of Parkin causes disrupted flux of pS65-Ub generated by PINK1
3. OMM proteins might be degraded by the proteasome after being tagged by pS65.

In combination with the additional findings:

* synthetic lethality of park deficient flies in combination with an Atg5 or Atg8a deficiency
* presence of pS65-Ub-positive structures in the flight muscles of park-deficient flies
* absence of these in wild-type and Pink1-deficient flies...,

the present manuscripts adds valuable insights on the mitophagy independent functions of the PINK1-Parkin pathway. As this controversy is still not resolved, the data described here is interesting. Overall the manuscript is clearly written, required controls are included, data acquisition/interpretation seems sound and the overall structure is clear.

-- We thank the reviewer for their positive comments on the work and appreciation of the novelty of these findings.

Mayor issues with the manuscript:

1. The Ub-clipping for data generation and interpretation thereof is key to understand the presented story. A brief explanation/summary (probably supported by cartoon) of the method might

be helpful for readers not familiar with the technique. As EMBO Reports has a broad readership, such a more explanatory section of the method should be included.

-- The Ub clipping method is indeed a very powerful approach for analysing higher-order Ub chain complexities, specifically chain branching, but in our early analyses we did not detect any branched chains, and so adapted the method for conventional analysis of diGly-modified Ub peptides rather than intact proteoforms. So, in fact, the Ub-clipping method is not central to the data interpretation in this instance, since the results presented could have been obtained with more conventional proteomic analysis, although the method did allow the simplification of sample processing. To avoid unnecessary confusion to the broad readership, we have removed specific mention of the Ub-clipping in the main text but retained the necessary details in Methods for those wishing to replicate this method.

Minor points:

2. Be consistent with writing concerning the PINK1-Parkin (or Pink1-parkin) pathway.

-- We apologise that this wasn't clear to the reader. We use "PINK1-Parkin" as the established nomenclature for human proteins and so specifically refers to the human proteins, whereas "Pink1-parkin" is the prescribed nomenclature for the fly counterparts. We prefer (and some journals insist) that the organism-specific nomenclature is adhered to, in order to retain clarity about precisely what is being referenced. This distinction is delineated in lines 41-42.

3. Figure 1B is not referred in text, should be line 128.

-- Figure 1B was cited in line 114 (now line 113).

4. I would remove the allele specification (example: Atg5- (Atg55cc5) from the main text. Specification in material/methods section is enough -> personal preference.

-- Thank you for this suggestion, but for full transparency we would prefer to keep this in the text as we feel it does not impinge on the readability of the text.

5. Line 262, "with only a few rare escapers" remove rare -> personal preference.

-- As above, we would prefer to retain this comment for full transparency in case others seek to replicate the result.

1st Revision - Editorial Decision 26th Aug 2022

Dear Alex,

Thank you for the submission of your revised manuscript to EMBO reports and thank you for providing feedback on the remaining concerns.

We note that the major concerns from referee #1 relate to novelty and the unclear degradation mechanism of pS65-Ub. The latter concern was shared by referee #2 but not seen as essential upon further discussion. We acknowledge that your data provides a more thorough and careful investigation of pS65-Ub production than the earlier study by Shiba-Fukushima et al and we also note the positive overall evaluation and support by referee #2 and former referee #3. On balance, we have therefore decided to proceed with publication. Please address all remaining concerns in a complete point-by-point response, please discuss and reference the earlier study by Shiba-Fukushima et al and please provide the modified Fig. EV5, showing different exposures to appreciate the kinetics of pS65-Ub decay upon lysosome and proteasome inhibition. You could also provide the data from the duplicate experiment as source data for this figure. Please also acknowledge the limitation, that the pathway of p-Ub turnover remains to elucidated.

In addition to these requests, there are also a number of things we need from the editorial side:

- Please provide up to 5 keywords.

- Please update the 'Conflict of interest' paragraph to our new 'Disclosure and competing interests statement'. For more information see
https://www.embopress.org/page/journal/14693178/authorguide#conflictsofinterest

- Regarding the Author Contributions, we now use CRediT to specify the contributions of each author in the journal submission system. CRediT replaces the author contribution section, please remove it from the manuscript text. Please use the free text box to provide more detailed descriptions.
See also guide to authors https://www.embopress.org/page/journal/14693178/authorguide#authorshipguidelines

- Figure callouts should follow a numerical/alphabetical order. Please rearrange figure panels, if possible:
- Fig EV2A is called out before EV1B.
- Fig EV3 is called out before EV1C.

- The file 'Supplement_MSdata_R1' (Supplementary table 1) appears to be a mix of source data related to some of the figures and of the mass spectrometry data. Is this correct? If so, please submit those parts that are source data as "Source data" and here we need individual files per each figure. Those sheets that report mass spectrometry datasets should be supplied as .xls file called "Dataset EV1" with this name and a legend in the first tab.

- Please add a heading 'Figure Legends'.

- Please supply unmodified source data for Figure 6 A-G.

- I attach to this email a related manuscript file with comments by our data editors. Please address all comments and upload a revised file with tracked changes with your final manuscript submission.

- Finally, EMBO reports papers are accompanied online by A) a short (1-2 sentences) summary of the findings and their significance, B) 2-3 bullet points highlighting key results and C) a synopsis image that is 550x200-600 pixels large (width x height) in .png format. You can either show a model or key data in the synopsis image. Please note that the size is rather small and that text needs to be readable at the final size. Please send us this information along with the revised manuscript.

We look forward to seeing a final version of your manuscript as soon as possible.

With kind regards,

Martina

Martina Rembold, PhD
Senior Editor
EMBO reports

****

Referee #1:

1. The novelty of the data is very limited. Shiba-Fukushima et al. already reported in 2017 (in a publication that is not cited in this manuscript) that young parkin knockout flies in basal conditions abundantly accumulate phospho-ubiquitin (Shiba-Fukushima et al. Evidence that phosphorylated ubiquitin signaling is involved in the etiology of Parkinson's disease. Hum Mol Genet 2017;26:3172-3185). The 'surprising finding' (line 340) of striking increase in pS65 Ub in parkin knockout flies in the absence of exogenous toxins was in fact already reported 5 years ago.
Importantly, the Shiba-Fukushima et al. study also assessed phospho-ubiquitin in brains and neurons from human PD patients with parkin mutations and found marked differences with Drosophila. In contrast to Drosophila, iPSC-derived human parkin-deficient neurons did not show any basal phospho-ubiquitin signal and, after mitochondrial depolarization, accumulated less phospho-ubiquitin than control neurons. This indicates that phospho-ubiquitin metabolism differs between Drosophila and humans and reduces the value of Drosophila models for investigation of phospho-ubiquitin metabolism from a human disease perspective.

2. The identity of the 'non-canonical' pS65-Ub turnover pathway that is proposed by the authors is completely obscure. The data in Fig. EV5 are uninterpretable, because there is no quantification and the western blots in EV5B, EV5C and especially EV5D are heavily overexposed. Without proper quantification of signals in the linear range of detection nothing can be concluded.
If neither lysosomal inhibitors nor proteasome inhibitors inhibit the disappearance of the p-Ub signal in the paraquat pulse-chase assay, the gradual disappearance of p-Ub is likely due to the action of an unidentified ubiquitin phosphatase in Drosophila.

Referee #2:

The point by point response provided by the author did address the majority of my comments. I am also pleased to note the author's efforts in carrying out additional experiments and adding new figures to support their response. Overall, I am satisfied with their response and the revisions done to their original manuscript.

1. The novelty of the data is very limited. Shiba-Fukushima et al. already reported in 2017 (in a publication that is not cited in this manuscript) that young parkin knockout flies in basal conditions abundantly accumulate phospho-ubiquitin (Shiba-Fukushima et al. Evidence that phosphorylated ubiquitin signaling is involved in the etiology of Parkinson's disease. Hum Mol Genet 2017;26:3172-3185). The 'surprising finding' (line 340) of striking increase in pS65 Ub in parkin knockout flies in the absence of exogenous toxins was in fact already reported 5 years ago.

Indeed, Shiba-Fukushima et al. reported that pS65-Ub accumulates in *parkin* mutant flies and we have now included appropriate citation and discussion of this work in our manuscript and adjusted the description of our own result. However, it should be made clear that this was a single set of observations (Shiba-Fukushima et al., Fig. 1B, C) with no further insights into its induction or degradation (the focus of our work). We have now gone on to characterise the production and degradation of pS65-Ub in much greater detail, using multiple orthogonal approaches.

It is worth noting that the antibody used by Shiba-Fukushima et al has not been widely used in the field, nor well validated. The immunostaining of fly brains they performed (mentioned in critique below) are extremely questionable, as the authors themselves note that equivalent signal was observed in *Pink1* null flies (essentially proving that it is non-specific). We maintain that our well-validated, thorough characterisation of the reagents and protocols described in our study provide a valuable resource to move the field forward. Overall, we do not consider that this single observation made by Shiba-Fukushima et al. substantively diminishes the novelty of the sum of our work.

Importantly, the Shiba-Fukushima et al. study also assessed phospho-ubiquitin in brains and neurons from human PD patients with parkin mutations and found marked differences with Drosophila. In contrast to Drosophila, iPSC-derived human parkin-deficient neurons did not show any basal phospho-ubiquitin signal and, after mitochondrial depolarization, accumulated less phospho-ubiquitin than control neurons. This indicates that phospho-ubiquitin metabolism differs between Drosophila and humans and reduces the value of Drosophila models for investigation of phospho-ubiquitin metabolism from a human disease perspective.

It is surprising that we still have to make these arguments nearly 20 years after first describing the *parkin* mutant fly model. It barely needs stating overtly that flies are different to humans, and one shouldn't expect their molecular cell biology to behave identically under all conditions. It should also now hardly need reiterating that the *Drosophila* models have delivered fundamental insights into the basic biology of Pink1 and parkin. Indeed, it was initially heavily criticised that the major tissues affected (and thus analysed) in *parkin* (and later *Pink1*) mutant flies were flight muscle and spermatids – seemingly utterly irrelevant for the human disease, yet this has proven highly informative for our understanding of disease aetiology. Thus, it is not revolutionary to learn that the kinetics of induction and/or turnover of pS65-Ub may differ between humans and an insect. Indeed, it is highly likely that different cell types or tissues will have different mechanisms of regulation for their quality control processes, and it is inappropriate to imply that iPSC-derived neurons are a 'gold-standard' model for the human disease condition. Nevertheless, as a model system, hypotheses can be formulated and tested that ultimately resolve the commonality between these organisms. Furthermore, it is for the field to resolve what experimental data are or are not going to be valuable in the broader context beyond the specific study. Something that often takes time (and replication) to become clear.

2. The identity of the 'non-canonical' pS65-Ub turnover pathway that is proposed by the authors is

completely obscure. The data in Fig. EV5 are uninterpretable, because there is no quantification and the western blots in EV5B, EV5C and especially EV5D are heavily overexposed. Without proper quantification of signals in the linear range of detection nothing can be concluded. If neither lysosomal inhibitors nor proteasome inhibitors inhibit the disappearance of the p-Ub signal in the paraquat pulse-chase assay, the gradual disappearance of p-Ub is likely due to the action of an unidentified ubiquitin phosphatase in Drosophila.

We entirely agree that the mechanism of turnover is not yet resolved and took care to make this interpretation clear in our writing. We also agree that some of the blots in V2 are on the heavy side of exposure. As can be appreciated, we frequently (but not always) found that 2 or 3 discrete bands dominated the sample view, but here it is important to appreciate the many proteins that are phospho-ubiquitinated. The presented exposure was a rationalised compromise in order for the reader to have a better appreciation of the 'smear' of fainter bands across the whole lane. We have now re-constructed the figure to show lower and higher exposure images to allow a fuller appreciation of the dynamic range (new Figure EV6). Nevertheless, we disagree that the blots are uninterpretable as, to us, the effect is quite obvious from the presented images - even more so from the improved presentation. There is clearly still a decline in pUb with either inhibitor alone that is lost with the combination of both. The reviewer seems to want to diminish these observations by positing some alternative (less interesting?) explanation.

In the end, this avenue of investigation took a considerable effort to work-up the protocols for inhibitor treatment *in vivo* in combination with toxification (not trivial), compounded by challenges of retrieval of sufficient sample after subcellular fractionation for immunoblotting. After nearly a year of revision work, it was apparent that a definitive answer on the degradation route would take considerably more time and concerted effort – something that the field collectively has not yet been able to resolve and is still an open question – and would be the focus of future work. We believe we described this result with an appropriate level of equivocation since it was clear to us that the current technical approach is not yet sufficient to be definitive. We have made additional comments in the text to highlight that the mechanism remains to be elucidated.

Dr. Alexander Whitworth
University of Cambridge
MRC Mitochondrial Biology Unit
Hills Road
Cambridge CB2 0XY
United Kingdom

Dear Alex,

We have now completed all revision checks and I am very pleased to accept your manuscript for publication in the next available issue of EMBO reports. Thank you for your contribution to our journal.

At the end of this email I include important information about how to proceed. Please ensure that you take the time to read the information and complete and return the necessary forms to allow us to publish your manuscript as quickly as possible.

As part of the EMBO publication's Transparent Editorial Process, EMBO reports publishes online a Review Process File to accompany accepted manuscripts. As you are aware, this File will be published in conjunction with your paper and will include the referee reports, your point-by-point response and all pertinent correspondence relating to the manuscript.

If you do NOT want this File to be published, please inform the editorial office within 2 days, if you have not done so already, otherwise the File will be published by default [contact: emboreports@embo.org]. If you do opt out, the Review Process File link will point to the following statement: "No Review Process File is available with this article, as the authors have chosen not to make the review process public in this case." Please note that the author checklist will still be published even if you opt out of the transparent process.

Thank you again for your contribution to EMBO reports and congratulations on a successful publication. Please consider us again in the future for your most exciting work.

Kind regards,

Martina

Martina Rembold, PhD
Senior Editor
EMBO reports

****

THINGS TO DO NOW:

Please note that you will be contacted by Wiley Author Services to complete licensing and payment information. The required 'Page Charges Authorization Form' is available here: https://www.embopress.org/pb-assets/embo-site/er_apc.pdf - please download and complete the form and return to embopressproduction@wiley.com

EMBO Press participates in many Publish and Read agreements that allow authors to publish Open Access with reduced/no publication charges. Check your eligibility: https://authorservices.wiley.com/author-resources/Journal-Authors/open-access/affiliation-policies-payments/index.html

You will receive proofs by e-mail approximately 2-3 weeks after all relevant files have been sent to our Production Office; you should return your corrections within 2 days of receiving the proofs.

Please inform us if there is likely to be any difficulty in reaching you at the above address at that time. Failure to meet our deadlines may result in a delay of publication, or publication without your corrections.

All further communications concerning your paper should quote reference number EMBOR-2021-53552V3 and be addressed to emboreports@wiley.com.

Should you be planning a Press Release on your article, please get in contact with emboreports@wiley.com as early as

possible, in order to coordinate publication and release dates.

EMBO PRESS

YOU MUST COMPLETE ALL CELLS WITH A PINK BACKGROUND ➔

PLEASE NOTE THAT THIS CHECKLIST WILL BE PUBLISHED ALONGSIDE YOUR PAPER

Corresponding Author Name: Alexander J. Whitworth
Journal Submitted to: EMBO Reports
Manuscript Number: #EMBOR-2021-53552

Reporting Checklist For Life Sciences Articles (Rev. June 2017)

This checklist is used to ensure good reporting standards and to improve the reproducibility of published results. These guidelines are consistent with the Principles and Guidelines for Reporting Preclinical Research issued by the NIH in 2014. Please follow the journal's authorship guidelines in preparing your manuscript.

A- Figures
1. Data
The data shown in figures should satisfy the following conditions:

- the data were obtained and processed according to the field's best practice and are presented to reflect the results of the experiments in an accurate and unbiased manner.
- figure panels include only data points, measurements or observations that can be compared to each other in a scientifically meaningful way.
- graphs include clearly labeled error bars for independent experiments and sample sizes. Unless justified, error bars should not be shown for technical replicates.
- if n< 5, the individual data points from each experiment should be plotted and any statistical test employed should be justified
- Source Data should be included to report the data underlying graphs. Please follow the guidelines set out in the author ship guidelines on Data Presentation.

2. Captions

Each figure caption should contain the following information, for each panel where they are relevant:

- a specification of the experimental system investigated (eg cell line, species name).
- the assay(s) and method(s) used to carry out the reported observations and measurements
- an explicit mention of the biological and chemical entity(ies) that are being measured.
- an explicit mention of the biological and chemical entity(ies) that are altered/varied/perturbed in a controlled manner.

- the exact sample size (n) for each experimental group/condition, given as a number, not a range;
- a description of the sample collection allowing the reader to understand whether the samples represent technical or biological replicates (including how many animals, litters, cultures, etc.).
- a statement of how many times the experiment shown was independently replicated in the laboratory.
- definitions of statistical methods and measures:
  - common tests, such as t-test (please specify whether paired vs. unpaired), simple χ2 tests, Wilcoxon and Mann-Whitney tests, can be unambiguously identified by name only, but more complex techniques should be described in the methods section;
  - are tests one-sided or two-sided?
  - are there adjustments for multiple comparisons?
  - exact statistical test results, e.g., P values = x but not P values < x;
  - definition of 'center values' as median or average;
  - definition of error bars as s.d. or s.e.m.

Any descriptions too long for the figure legend should be included in the methods section and/or with the source data.

In the pink boxes below, please ensure that the answers to the following questions are reported in the manuscript itself. Every question should be answered. If the question is not relevant to your research, please write NA (non applicable). We encourage you to include a specific subsection in the methods section for statistics, reagents, animal models and human subjects.

USEFUL LINKS FOR COMPLETING THIS FORM

http://www.antibodypedia.com
http://1degreebio.org
http://www.equator-network.org/reporting-guidelines/improving-bioscience-research-report

http://grants.nih.gov/grants/olaw/olaw.htm
http://www.mrc.ac.uk/Ourresearch/Ethicsresearchguidance/Useofanimals/index.htm
http://ClinicalTrials.gov
http://www.consort-statement.org
http://www.consort-statement.org/checklists/view/32-consort/66-title
http://www.equator-network.org/reporting-guidelines/reporting-recommendations-for-tumo
http://datadryad.org
http://figshare.com
http://www.ncbi.nlm.nih.gov/gap
http://www.ebi.ac.uk/ega
http://biomodels.net/
http://biomodels.net/miriam/
http://jjj.biochem.sun.ac.za
https://osp.od.nih.gov/biosafety-biosecurity-and-emerging-biotechnology/
http://www.selectagents.gov/

B- Statistics and general methods

Please fill out these boxes ➔ (Do not worry if you cannot see all your text once you press return)

1.a. How was the sample size chosen to ensure adequate power to detect a pre-specified effect size?	Effect size calculations were not performed but sample sizes were guided by previous experiences. Due to the large amount of sample required per replicate for mass spectrometry experiments, it was typically only feasible to perform 3 replicates. The only exception was the analysis of 60-day-old flies, for which four replicates were prepared due to the time taken to obtain the flies, to account for the risk that one or more replicate may not yield sufficient material for analysis. We acknowledge in the results that this may cause 'real' differences with small effect sizes to fail to reach statistical significance.
1.b. For animal studies, include a statement about sample size estimate even if no statistical methods were used.	For western blotting experiments, three replicate experiments were performed using different batches of animals. These experiments typically gave highly consistent results, and therefore high confidence in the results obtained when three replicates were performed. For microscopy experiments, 4 or 5 independent replicates were performed per genotype, to account for higher variability in the microscopy data, and for the possibility that some replicates may need to be excluded as noted below due to technical reasons.
2. Describe inclusion/exclusion criteria if samples or animals were excluded from the analysis. Were the criteria pre-established?	No samples were specifically included or excluded except in instances of 'normal' experimental failure such as tissue disruption during dissection or poor antibody labelling.
3. Were any steps taken to minimize the effects of subjective bias when allocating animals/samples to treatment (e.g. randomization procedure)? If yes, please describe.	NA
For animal studies, include a statement about randomization even if no randomization was used.	No specific measures for randomisation wer used, as animals were typically all treated in the same way. However, animals of the appropriate genotype/condition were assumed to be identical and randomly selected from the progeny/cohort.
4.a. Were any steps taken to minimize the effects of subjective bias during group allocation or/and when assessing results (e.g. blinding of the investigator)? If yes please describe.	As below.
4.b. For animal studies, include a statement about blinding even if no blinding was done	For the analysis of microscopy data, investigator bias was minimised by using a computational approach with ImageJ. The parameters (background subtraction, thresholding) were determined using a small subset of data (approximately one third of the complete dataset, comprising a mixture of genotypes), then the same settings were applied to the remaining images without further adjustment.
5. For every figure, are statistical tests justified as appropriate?	Yes, as indicated. Statistical tests were not performed for the mass spectrometry analysis of pS65-Ub levels in different conditions, as this was an exploratory study not suited to statistical analysis.
Do the data meet the assumptions of the tests (e.g., normal distribution)? Describe any methods used to assess it.	Number of replicates was often too small to assess whether the spread was normal. A normal distribution was therefore assumed.

Is there an estimate of variation within each group of data?	This was not estimated a priori as no preexisting data for similar approaches were available prior to our study.
Is the variance similar between the groups that are being statistically compared?	Not always - in cases in which there were a large range of data points, including zero values for some genotypes (eg. pS65-Ub leves in Pink1- compared with park-/- animals), this resulted in heteroscedasticity of the data. In these cases, data were log-transformed prior to statistical analysis as indicated.

C- Reagents

6. To show that antibodies were profiled for use in the system under study (assay and species), provide a citation, catalog number and/or clone number, supplementary information or reference to an antibody validation profile. e.g., Antibodypedia (see link list at top right), 1DegreeBio (see link list at top right).	Catalog numbers and suppliers are quoted for all antibodies in the materials and methods. For the pS65-Ub antibody, we cite an earlier paper validating the antibody, in addition to providing our own validation in the results.
7. Identify the source of cell lines and report if they were recently authenticated (e.g., by STR profiling) and tested for mycoplasma contamination.	NA

* for all hyperlinks, please see the table at the top right of the document

D- Animal Models

8. Report species, strain, gender, age of animals and genetic modification status where applicable. Please detail housing and husbandry conditions and the source of animals.	This information is stated in the methods
9. For experiments involving live vertebrates, include a statement of compliance with ethical regulations and identify the committee(s) approving the experiments.	NA
10. We recommend consulting the ARRIVE guidelines (see link list at top right) (PLoS Biol. 8(6), e1000412, 2010) to ensure that other relevant aspects of animal studies are adequately reported. See author guidelines, under 'Reporting Guidelines'. See also: NIH (see link list at top right) and MRC (see link list at top right) recommendations. Please confirm compliance.	We have read and understood the ARRIVE 2.0 guidelines.

E- Human Subjects

11. Identify the committee(s) approving the study protocol.	NA
12. Include a statement confirming that informed consent was obtained from all subjects and that the experiments conformed to the principles set out in the WMA Declaration of Helsinki and the Department of Health and Human Services Belmont Report.	NA
13. For publication of patient photos, include a statement confirming that consent to publish was obtained.	NA
14. Report any restrictions on the availability (and/or on the use) of human data or samples.	NA
15. Report the clinical trial registration number (at ClinicalTrials.gov or equivalent), where applicable.	NA
16. For phase II and III randomized controlled trials, please refer to the CONSORT flow diagram (see link list at top right) and submit the CONSORT checklist (see link list at top right) with your submission. See author guidelines, under 'Reporting Guidelines'. Please confirm you have submitted this list.	NA
17. For tumor marker prognostic studies, we recommend that you follow the REMARK reporting guidelines (see link list at top right). See author guidelines, under 'Reporting Guidelines'. Please confirm you have followed these guidelines.	NA

F- Data Accessibility

18: Provide a "Data Availability" section at the end of the Materials & Methods, listing the accession codes for data generated in this study and deposited in a public database (e.g. RNA-Seq data: Gene Expression Omnibus GSE39462, Proteomics data: PRIDE PXD000208 etc.) Please refer to our author guidelines for 'Data Deposition'.<br><br>Data deposition in a public repository is mandatory for:<br>a. Protein, DNA and RNA sequences<br>b. Macromolecular structures<br>c. Crystallographic data for small molecules<br>d. Functional genomics data<br>e. Proteomics and molecular interactions	Our mass spectrometry data is a targeted study and is not in a suitable format for submission to a proteomics data repository. We have instead included the relevant data in the supplementary table, and will make the raw data available upon request.
19. Deposition is strongly recommended for any datasets that are central and integral to the study; please consider the journal's data policy. If no structured public repository exists for a given data type, we encourage the provision of datasets in the manuscript as a Supplementary Document (see author guidelines under 'Expanded View' or in unstructured repositories such as Dryad (see link list at top right) or Figshare (see link list at top right).	See above
20. Access to human clinical and genomic datasets should be provided with as few restrictions as possible while respecting ethical obligations to the patients and relevant medical and legal issues. If practically possible and compatible with the individual consent agreement used in the study, such data should be deposited in one of the major public access-controlled repositories such as dbGAP (see link list at top right) or EGA (see link list at top right).	NA
21. Computational models that are central and integral to a study should be shared without restrictions and provided in a machine-readable form. The relevant accession numbers or links should be provided. When possible, standardized format (SBML, CellML) should be used instead of scripts (e.g. MATLAB). Authors are strongly encouraged to follow the MIRIAM guidelines (see link list at top right) and deposit their model in a public database such as Biomodels (see link list at top right) or JWS Online (see link list at top right). If computer source code is provided with the paper, it should be deposited in a public repository or included in supplementary information.	NA

G- Dual use research of concern

22. Could your study fall under dual use research restrictions? Please check biosecurity documents (see link list at top right) and list of select agents and toxins (APHIS/CDC) (see link list at top right). According to our biosecurity guidelines, provide a statement only if it could.	
